# Novel non-ATP competitive small molecules targeting the CK2 α/β interface

**DOI:** 10.1016/j.bmc.2018.05.011

**Published:** 2018-07-15

**Authors:** Paul Brear, Andrew North, Jessica Iegre, Kathy Hadje Georgiou, Alexandra Lubin, Laura Carro, William Green, Hannah F. Sore, Marko Hyvönen, David R. Spring

**Affiliations:** a Department of Biochemistry, University of Cambridge, Sanger Building, 80 Tennis Court Road, Old Addenbrooke’s Site, Cambridge CB2 1GA, UK; b Department of Chemistry, University of Cambridge, Lensfield Road, Cambridge CB2 1EW, UK

**Keywords:** CK2, Protein-protein interaction, Fragment based drug discovery

## Abstract

Increased CK2 levels are prevalent in many cancers. Combined with the critical role CK2 plays in many cell-signaling pathways, this makes it a prime target for down regulation to fight tumour growth. Herein, we report a fragment-based approach to inhibiting the interaction between CK2α and CK2β at the α-β interface of the holoenzyme. A fragment, CAM187, with an IC_50_ of 44 μM and a molecular weight of only 257 gmol^−1^ has been identified as the most promising compound. Importantly, the lead fragment only bound at the interface and was not observed in the ATP binding site of the protein when co-crystallised with CK2α. The fragment-like molecules discovered in this study represent unique scaffolds to CK2 inhibition and leave room for further optimisation.

## Graphical abstract

**Figure:**
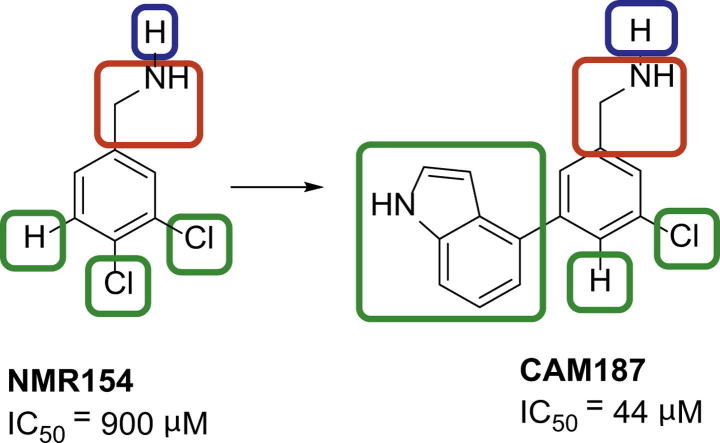

## 1 Introduction

### 1.1 CK2 as a potential target

CK2 is a ubiquitous protein kinase and is overexpressed in a large range of cancers.^1^ As a protein kinase, CK2 phosphorylates many substrates involved in cell growth.^2^ Unnaturally high quantities of CK2 and its associated activity have been observed in many tumours.^3^ In healthy cells, CK2 is diffused in the various cellular compartments and is up regulated during cell proliferation, but readily returns to normal levels. However, in tumour cells there are high levels of CK2 about the nucleus, and during cell proliferation, CK2 finds homoeostasis at its new high level.^4^, ^5^ CK2, as well as being involved in cell proliferation and growth, also suppresses apoptosis, linking CK2 to the cancer phenotype.^6^

CK2 inhibition may be a particularly effective form of treatment as it has a pivotal and indispensable role in the cells. If inhibited below the high critical threshold, the cancerous cell would no longer be malignant as there are no alternative signaling pathways which could by-pass the need for CK2.^7^ Due to the role of CK2 in cell growth, proliferation, and apoptosis, its down regulation is catastrophic to cancer cells. Its validation as an anti-cancer target is shown in the fact CK2 inhibitors have entered clinical trials.^8^

### 1.2 CK2 inhibition strategies

Previous research into CK2 inhibition has focused on small molecules that inhibit the kinase activity at the ATP-binding site.^9^, ^10^, ^11^ Strategies that focus on the ATP-binding site for inhibition often suffer from off-target action due to the highly conserved nature of the ATP pocket across the genome.^12^, ^13^ Indeed, the current clinical candidate, CX4945, inhibits CK2 at the ATP pocket and shows off-target activity against 13 proteins with nanomolar IC_50_. Additionally, in one study CX4945 was shown to inhibit CLK2 more potently than CK2 (3.8 nM compared to 14.7 nM).^14^

Alternative strategies to selectively inhibit CK2 have recently been explored; these exploit other aspects of the protein unique to CK2. The αD pocket, which is unique to CK2, was discovered near the ATP site. It has been exploited by linking a low affinity ATP site fragment to an elaborated fragment in the αD site. This led to a novel and selective CK2 inhibitor.^14^, ^15^

**Fig. 1:**
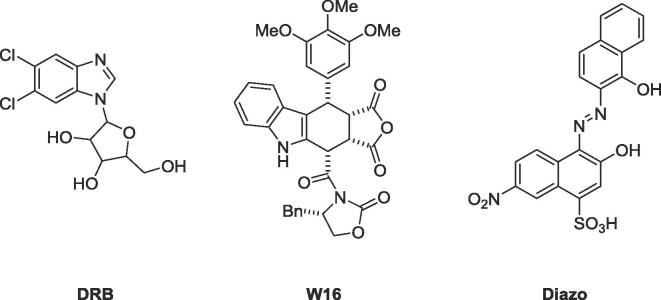
Three previous inhibitors of the CK2 interface: DRB, W16, and the diazodinaphthalene compound.

One of the other areas to probe for non-ATP site inhibition is the protein-protein interaction (PPI) interface between the catalytic and regulatory subunits of CK2: CK2α and CK2β. To date three small molecules and a peptide have been shown to bind in the interface pocket on CK2α. The fragment, DRB, binds at both the ATP and the α/β interface site (Fig. 1).^16^ W16 and a diazodinaphthalene compound are believed to bind at the interface site although no crystal structures have been published to validated this. Additionally, W16 and the diazodinaphthalene compound are large molecules with some undesirable physicochemical properties, therefore do not make ideal lead candidates for further drug discovery (Fig. 1).^17^, ^18^ Finally, a cyclic peptide, *Pc*, was found to inhibit the interaction between CK2α and CK2β.^19^

### 1.3 Ideal chemical fragment

The fragment-based approach to drug discovery utilises small compounds (molecular weight ≤300 gmol^−1^) to identify starting points against any target of interest.^20^ Although fragments may bind with weak affinity they tend to have high intrinsic binding energy, high ligand efficiency, and readily optimizable physiochemical properties. These fragments can then be expanded to increase the potency whist maintaining the compound’s drug-like properties.^21^

Due to the usefulness of fragments as a tool for drug discovery, work has been done to explore the idea of the ideal chemical fragment. Studies have suggested that a ‘Rule of Three’^22^ (mirroring Lipinski’s Rule of Five^23^) is a useful method for measuring how ‘fragment-like’ molecules are.

Herein, we report the discovery of a novel fragment which binds to the CK2α/CK2β interface pocket. This lead fragment could be modified to give new CK2 inhibitors that show limited off-target activity.

## 2 Material and methods

### 2.1 Chemistry

#### 2.1.1 Experimental procedures

**Scheme 1:**
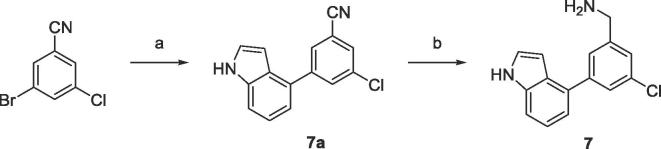
(a) CH_2_Cl_2_, Py, Tf_2_O, (b) ArB(OH)_2_, K_2_CO_3_, DCE, Pd(PPh_3_)_4_, (c) LiAlH_4_, Et_2_O, AlCl_3_. Full procedures are detailed in the Supporting Information.

Compounds **2**, **4**, and **5** were synthesised from commercially available 3-chloro-4-bromobenzonitrile. This was subjected to a Suzuki-Miyaura cross-coupling with the relevant aryl boronic acid to produce a biaryl nitrile. This nitrile was reduced to give the final compounds. Compound **3** was synthesised using a similar procedure; however, starting from commercially available 3-chloro-4-hydroxybenzonitrile. This was triflated and then subjected to the above reactions as detailed in Scheme 1.

**Scheme 2:**
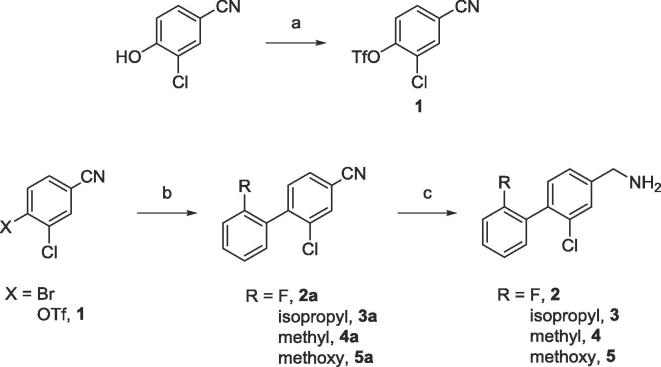
(a) Indole-4-boronic acid, K_3_PO_4_, PCy_3_, Pd_2_(dba)_3_, 1,4-dioxane, (b) LiAlH_4_, AlCl_3_, Et_2_O.

Compound **7** was synthesised from commercially available 3-chloro-5-bromobenzonitrile and indole-4-boronic acid *via* Suzuki-Miyaura cross-coupling followed by nitrile reduction as shown in Scheme 2.

#### 2.1.2 Detailed procedures and characterization

Detailed procedures and characterisation of all compounds reported in this paper and their precursors can be found in the Supporting Information.

### 2.2 Biological assays

Protein expression and purification, X-ray crystallography, and FP assays can be found in the Supporting Information of this paper and our recently published papers.^14^, ^15^

## 3 Results and discussion

### 3.1 Initial hits of NMR154

**Fig. 2:**
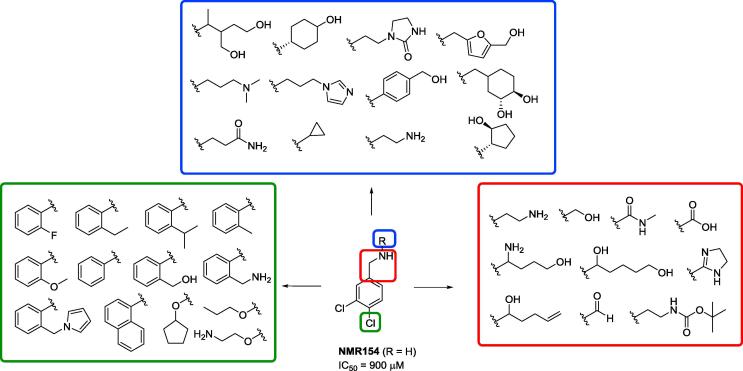
A selection of the analogues of NMR154 synthesised. The analogues in the blue box maintained all the features of NMR154 but added additional functionality onto the nitrogen. The red box shows analogues which kept the dichlorobenzene moiety but substituted the methylamine for the groups shown. Finally, the green box shows substitutions made to the chlorine highlighted in green.

The Abell Research Group (at Cambridge University) conducted initial high throughput screening of fragments from their fragment library against CK2 using an FP (fluorescence polarization) assay. This library consisted of over 400 compounds from the Abell Group. The most promising fragment from this screen was NMR154 (Fig. 2).^24^ This compound was found to bind in both the ATP pocket and the α-β interface site.

**Fig. 3:**
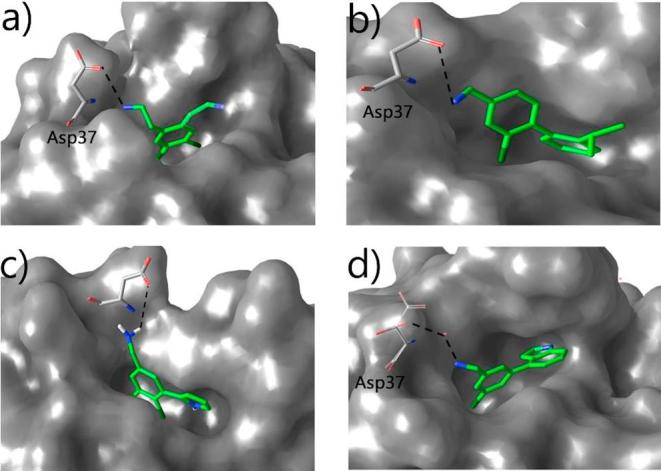
A side-by-side depiction of five fragments binding in the interface site of CK2α. a) NMR154L binds so that the dichloro moiety anchors the fragment into the hydrophobic pocket (pdb: 5CLP). The ethylamino group juts out of the pocket to form an H-bond with Asp37 outside the pocket. b) Compound **3** maintains the H-bond with Asp37. The second aromatic ring provides a better hydrophobic interaction in the pocket as it occupies more of the space. c) Compound **6** shows some promise as an interface binder. The interaction with Asp37 is maintained as well as the biaryl core occupying the width of the pocket. It is clear from the image there is an unoccupied area of the pocket beneath the pyrrole ring. This is a potential area for growth. d) The lead fragment **CAM187 (7)**, like its predecessors, forms an H-bond with Asp37. This time with the aid of a bridging water molecule. In indole moiety provides the best hydrophobic interactions out of the molecules tested. It provides both the with to fill the pocket as well as the depth to fill it.

A crystal structure was obtained of NMR154L, the ethyl analogue of NMR154, (Fig. 3a) showed a molecule each binding in the interface pocket as well as the ATP site and the αD site. This showed the dichlorobenzene part of the molecule anchoring in the pocket while the ethylamino moiety protruded from the pocket to interact with an aspartate residue of the protein (Asp37 is on the edge of the α-β interface site and forms a salt bridge with Arg186 from CK2β).

With this crystal structure in hand, it was hoped that the binding could be improved by modifying NMR154 along a series of vectors, utilizing anchoring in the pocket and increasing the interactions outside the pocket with the protein residues (Fig. 2).

### 3.2 Fragment development

The first iteration explored was substituting the methylamine group (the red box in Fig. 2) to enhance or pick up new interactions outside the pocket. An additional 12 compounds were synthesised with a variety of functionality coupled with alkyl chains of various lengths. Functionality explored were amines, alcohols, amides, carboxylic acids, dihydroimidazole, aldehyde, carbamate, as well as compounds with bifunctionality like an amino alcohol and a diol. Disappointingly, only one of the methylamino variants (ethylamino, NMR154L) showed minimal improvement compared to NMR154 (900 μM vs 700 μM). As no significant improvement in binding affinity was observed with the ethylene analogue, the second iteration of compounds continued to use the methylene linkage. In addition, these analogues were easier to synthesise as they required only a reduction of the nitrile to obtain the final compounds.

Following exploration of the methylene linkage, substitution on the amine was explored next (the blue box in Fig. 2). In this iteration 26 compounds were made. It was hoped that elaborating off this position would exploit any new H-bond interactions, in addition to maintaining the interaction with Asp37. Thus, a variety of alcohols were chosen as well as a carbamide, amine, imidazole, amide, and morpholine. Again, none of the nitrogen substituted analogues showed an improvement upon NMR154 (details given in Supporting Information).

Having investigated variations of the methylene linkage and amino group substitution, further studies were made into varying other parts of the benzene ring (the vectors highlighted in green in Fig. 2). From the crystal structures we expected that modification of the substituents and the aryl group itself would result in a better fit in the pocket and therefore increased binding affinity. At total of 16 compounds were synthesised. A selection of aromatic and aliphatic groups were chosen including a variety of substituted benzene rings, a naphthalene, and a pyrrole. A range of ethers were also tested with alkyl groups and an amine.

Pleasingly, this substitution showed the greatest improvement in inhibition with all the dibenzene compounds showing an IC_50_ of <500 μM (see Supplementary Information). Unfortunately, the pyrrole and aliphatic compounds showed no improvement. This would suggest that the greatest improvement to be made to NMR154 would be to more firmly anchor it into the pocket rather than to utilising bonding interactions with residues outside the pocket.

A crystal structure of **3** binding in the pocket was obtained (Fig. 3b). This clearly showed that the electrostatic interaction between the amino moiety and Asp37 was maintained. In addition, the 2-isopropylbenzene moiety is occupying the pocket both in terms of depth and width. However, for the biaryl compounds, electron density was also observed in the αD site.

### 3.3 Improving selectivity for α/β interface binding only

The αD pocket being deep and narrow favours the *para* disposition of the aryl ring and the methylamino.^15^ In order to improve selectivity so that the fragments only bound at the α/β interface a series a *meta* disposed compound was synthesised and tested.

This hypothesis was shown to be correct, and the *meta* biaryl compound tested bound exclusively at the interface (entry **6** in Table 1).

**Table 1:** Summary of compounds with improved binding.

Compound	Structure	ClogP^a^	IC_50_ (μM)^b^
NMR154		2.60	900
**2**		3.49	150
**3**		4.60	205
**4**		3.86	208
**5**		3.19	493
**6**		2.91	3000
**7, CAM187**		3.45	44

a Calculated of partition coefficient.

b Half maximal inhibitory concentration.

Although having a high IC_50_ of 3 mM examination of the crystal structure of compound **6** showed the biaryl core lying on its side taking up the width of the pocket with the amine interacting with Asp37 (Fig. 3c). It was theorized that increasing the size of the pyrrole to an indole would occupy more of the depth of the pocket increasing the potency of the fragment. In addition, a chlorine on the benzene ring was removed to decrease the clogP (calculated logarithm of the partition coefficient).

Pleasingly, these judgements were correct, and the new fragment, **CAM187**, gave the best IC_50_ of 44 μM. A crystal structure of the fragment using the CK2α construct *CK2a_KA* showed it only bound in the interface pocket. The Cl fills the phenyl pocket and the amine interacts with Asp37 as with the original fragment. Whereas, the indole ring sits under the β4β5 loop and the nitrogen of the indole interacts with the OH group of THR108. (Fig. 3d).

**CAM187** has advantages over previously discovered interface inhibitors because of its fragment-like nature (see Table 2). W16 (IC_50_ ≈ 20 μM)^17^ and the diazo compound (IC_50_ ≈ 0.4 μM)^18^ are already at the top end for molecular weight and leave little room for elaboration to improve their potency and drug-like properties. likely exhibit undesired biological properties.^16^ Further analysis of CAM187 showed that it did not inhibit the phosphorylation activity of CK2α at 100 μM. (% inhibition 18% at 100 μM). This confirms that CAM187 does not significantly bind in the ATP site of CK2 at these concentrations.

**Table 2:** Comparison of the fragment-like properties of CAM187 and DRB alongside the drug-like properties of W16 and Diazo.^25^

^a^MW: molecular weight in gmol^−1^, clogP: calculated log of the partition coefficient, PSA: polar surface area, HBA: hydrogen-bond acceptors, HBD: hydrogen-bond donors, RBC: rotatable bond count, CC: chiral centres, ATP: does the compound bind in the ATP pocket of CK2?

^b^Ideal range from Congreve et al.^22^ Green is within ideal range, amber is within 15% of ideal range, red is over 15% from ideal range.

^c^Lipinski’s ‘Rule of Five’.^25^

## 4 Conclusion

By screening the NMR compound library an initial hit was found for an inhibitor of the PPI between the α-β subunits of CK2 with an IC_50_ of 900 μM. This compound was modified and elaborated through several vectors. Through five iterations, 63 compounds were synthesised and tested against CK2. Through the first three iterations (elaborating at each of the vectors highlighted in Fig. 2), it was found that a series of biaryl compounds were the best inhibitors of the PPI with the lead compound at that stage (**2**) giving an IC_50_ of 150 μM. Realising this trend additional biaryl compounds were screened against CK2 to give the lead compound, **CAM187** (**7**). These compounds affect their binding by the biaryl part of the molecule filling the PPI pocket on the protein and the pendent ammonium group forming an H-bond with an Asp37 above the pocket.

**CAM187** is the first small molecule fragment to inhibit the α-β interface of CK2 without also binding in the ATP site. Due to its fragment-like properties, **CAM187** leaves room for further development. These developments could lead to drug-like molecules with high potency and selectivity against CK2 as well as deeper understanding between the structure activity relationship.

## References

[1] Guerra B., Issinger O.-G. (2016). Curr Med Chem.

[2] Guerra B., Issinger O.-G. (1999). Electrophoresis.

[3] Chua M., Ortega C., Sheikh A. (2017). Pharmaceuticals.

[4] Faust R.A., Niehans G., Gapany M. (1999). Int J Biochem Cell Biol.

[5] Ahmad K.A., Wang G., Unger G., Slaton J., Ahmed K. (2008). Adv Enzyme Regul.

[6] Ahmed K., Gerber D.A., Cochet C. (2002). Trends Cell Biol.

[7] Trembley J.H., Chen Z., Unger G. (2010). BioFactors.

[8] Chon H.J., Bae K.J., Lee Y., Kim J. (2015). Front Pharmacol.

[9] Prudent R., Cochet C. (2009). Chem Biol.

[10] Sarno S., de Moliner E., Ruzzene M. (2003). Biochem J.

[11] Siddiqui-Jain A., Drygin D., Streiner N. (2010). Cancer Res.

[12] Battistutta R. (2009). Cell Mol Life Sci.

[13] Fabian M.A., Biggs W.H., Treiber D.K. (2005). Nat Biotechnol.

[14] Brear P., De Fusco C., Hadje Georgiou K. (2016). Chem Sci.

[15] De Fusco C., Brear P., Iegre J. (2017). Bioorg Med Chem.

[16] Raaf J., Brunstein E., Issinger O.-G., Niefind K. (2008). Chem Biol.

[17] Laudet B., Moucadel V., Prudent R. (2008). Mol Cell Biochem.

[18] Moucadel V., Prudent R., Sautel C.F. (2011). Oncotarget.

[19] Zhou Y., Zhang N., Chen W., Zhao L., Zhong R. (2016). Phys Chem Chem Phys.

[20] Johnson C.N., Erlanson D.A., Murray C.W., Rees D.C. (2017). J Med Chem.

[21] Ciulli A., Abell C. (2007). Curr Opin Biotechnol.

[22] Congreve M., Carr R., Murray C., Jhoti H. (2003). Drug Discov Today.

[23] Lipinski C.A., Lombardo F., Dominy B.W., Feeney P.J. (2001). Adv Drug Deliv Rev.

[24] Seetoh, W., Stubbs, C., Dickson, C., Hyvönen, M., Matak-Vinković, D., & Abell, C. Research Data Supporting “Achieving small–molecule disruption of the CK2α/CK2β protein–protein interaction within the protein kinase CK2 heterotetramer” https://doi.org/10.17863/CAM.189.

[25] Veber D.F., Johnson S.R., Cheng H.-Y., Smith B.R., Ward K.W., Kopple K.D. (2002). J Med Chem.

