# Supplementary material for: Novel non-ATP competitive small molecules targeting the CK2 α/β interface
Source: Bioorg Med Chem. 2018 Jul 15;26(11):3016–20. doi: 10.1016/j.bmc.2018.05.011 (PMC6562204; doi:10.1016/j.bmc.2018.05.011)
Supplement: Supplementary data [file mmc1.docx]

Supporting Information

Novel non-ATP competitive small molecules targeting the CK2 α/β interface

Paul Brear, Andrew North, Jessica Iegre, Kathy Hadje Georgiou, Alexandra Lubin, Laura Carro Santos, William Green, Hannah Sore, Marko Hyvönen and David Spring

Contents

SI_1 General experimental methods 03

SI_2 Detailed experimental procedures and compound characterisation 05

SI_3 List of fragments screened 33

SI_4 Selected NMR Spectra 39

SI_5 Materials and Methods 64

SI_6 Crystallographic data 66

SI_1 General experimental methods

**Reactions** For reactions requiring anhydrous conditions, experiments were carried out in oven-dried glassware. Unless otherwise stated, all reactions were carried out under nitrogen atmosphere. Room temperature (RT) refers to ambient temperature. All temperatures below 0 °Care that of the external bath. Temperatures of 0 °C were maintained using an ice-water bath. Temperatures of –15 °C were maintained using a salt and ice-water bath. Temperatures of –78 °C were maintained using an acetone-cardice bath.

**Solvents and reagents** Solvents and commercially available reagents were dried and purified before use, where appropriate using standard procedures. Toluene, hexane, diethyl ether, ethyl acetate, methanol, THF, and dichloromethane were dried and distilled using standard methods from oxygen free from solvent dispenser units under an argon atmosphere.

**Chromatography** Analytical thin layer chromatography (TLC) was performed using pre-coated Merck glass backed silica gel plates (Silica gel 60 F254). Flash column chromatography was undertaken on Fluka or Material Harvest silica gel (230–400 mesh) under a positive pressure of nitrogen unless otherwise stated. Visualization was achieved using ultraviolet light (254 nm) and chemical staining with basic potassium permanganate solution as appropriate. Retention factors (R_f_) are quoted to 0.01.

**Infrared spectra** Infrared (IR) spectra were recorded on a Perkin Elmer 1FT-IR Spectrometer fitted with an ATR sampling accessory as either solids or neat films, either through direct application or deposited in CDCl_3_. Absorption maxima (*ν*_max_) are reported in wavenumbers (cm^–1^) with the following abbreviations: w, weak; m, medium; s, strong; br, broad.

**NMR spectra** Magnetic resonance spectra were processed using TopSpin v. 3.5 (Bruker). Measured coupling constants (*J*) are reported for mutually coupled signals; coupling constants are labelled apparent in the absence of an observed mutual coupling, or multiplet when none can be determined.

Proton magnetic resonance spectra were recorded using an internal deuterium lock (at 298 K unless stated otherwise) on Bruker DPX (400 MHz; 1H-13C DUL probe), Bruker Avance III HD (400 MHz; Smart probe), Bruker Avance III HD (500 MHz; Smart probe) and Bruker Avance III HD (500 MHz; DCH Cryoprobe) spectrometers. Chemical shifts (δ_H_) are quoted in ppm to the nearest 0.01 ppm and are referenced to the residual non-deuterated solvent peak. Discernible coupling constants for mutually coupled protons are reported as measured values in Hertz, rounded to the nearest 0.5 Hz. Data are reported as: chemical shift, multiplicity (br = broad; s = singlet; d = doublet; t = triplet; q = quartet; qn = quintet; sp = septet; m = multiplet; or a combination thereof), coupling constants, number of nuclei, and assignment. Diastereotopic protons are assigned as Xa and Xb, where Xb designates the lower-field proton.

Carbon magnetic resonance spectra were recorded using an internal deuterium lock (at 298 K unless stated otherwise) on Bruker DPX (101 MHz), Bruker Avance III HD (101 MHz) and Bruker Avance III HD (126 MHz) spectrometers with broadband proton decoupling. Carbon spectra assignments are supported by DEPT editing, ^1^H–^13^C HSQC or ^1^H–^13^C HMBC spectra, or by analogy. Chemical shifts (δ_C_) are quoted in ppm to the nearest 0.1 ppm and are referenced to the deuterated solvent peak. Data are reported as: chemical shift, number of nuclei (if not one), multiplicity (if not a singlet), and coupling constants

**Mass spectra** High-resolution mass spectra (HRMS) were measured on a Micromass LCT Premier spectrometer using electron spray ionization (ESI) techniques. Masses are quoted within the 5 ppm error limit.

**Melting points** Melting points were obtained on a Buchi B-545 melting point apparatus and are uncorrected.

SI_2 Detailed experimental procedures and compound characterisation

2-chloro-2'-fluoro-[1,1'-biphenyl]-4-carbonitrile, **2a**

3-chloro-4-bromobenzonitrile (300 mg, 1.39 mmol 1.0 equiv), 2-fluorophenylboronic acid (194 mg, 1.39 mmol 1.0 equiv), Pd(PPh_3_)_4_ (0.05 equiv) and K_2_CO_3_ (2.0 equiv) were weighed into a microwave tube and solvated with DME (3.0 mL) and H_2_O (1.0 mL). The reaction mixture was degassed by bubbling nitrogen through the solution for 5 minutes and then heated to 100 °C under microwave irradiation for 2 hours. The reaction was allowed to cool to room temperature, filtered through celite washing with Et_2_O and the solvent removed under reduced pressure. The residue was dissolved in CH_2_Cl_2_/H_2_O and extracted three times with CH_2_Cl_2_. The combined organic extracts were washed with a saturated aqueous solution of NaCl, dried over MgSO_4_, filtered and concentrated *in vacuo*. The crude product was purified by flash column chromatography (silica gel, 1:9 EtOAc:pet ether) to provide the title compound as a white solid (58 mg, 0.25 mmol, 18%):

**R*_f_*** 0.50 (1:9 EtOAc:pet ether); **m.p.** 46-48 °C; **IR** υ_max_ 1474, 1206, 1073, 768, 604; **^1^H NMR** (400 MHz, CDCl_3_) δ 7.73 (d, *J* = 1.5 Hz, 1H), 7.57 (dd, *J* = 7.9, 1.6 Hz, 1H), 7.44 – 7.35 (m, 2H), 7.28 – 7.17 (m, 2H), 7.17 – 7.09 (m, 1H); **^13^C NMR** (101 MHz, CDCl_3_) δ 159.3 (d, *J* = 248.8 Hz, 1C), 140.1, 134.9, 133.1, 132.6 (d, *J* = 1.1 Hz, 1C), 131.1, 131.0 (d, *J* = 12.7 Hz, 1C), 130.3, 125.4 (d, *J* = 15.5 Hz, 1C), 124.3 (d, *J* = 3.7 Hz, 1C), 117.5, 116.1 (d, *J* = 21.7 Hz, 1C), 113.4; **HRMS** (ESI) calcd for [C_13_H_7_^35^Cl^19^FN + H]^+^: 232.0329, found: 232.0322.

2-Chloro-2'-fluoro-[1,1'-biphenyl]-4-carbonitrile, 2

3-chloro-4-bromobenzonitrile (300 mg, 1.39 mmol, 1.0 equiv), 2-fluorophenylboronic acid (194 mg, 1.39 mmol, 1.0 equiv), Pd(PPh_3_)_4_ ((80 mg, 0.07 mmol, 0.05 equiv) and K_2_CO_3_ (383 mg, 2.77 mmol, 2.0 equiv) were weighed into a microwave tube and solvated with DME (3.0 mL) and H_2_O (1.0 mL). The reaction mixture was degassed by bubbling nitrogen through the solution for 5 minutes and then heated to 100 °C under microwave irradiation for 2 hours. The reaction was allowed to cool to room temperature, filtered through celite washing with Et_2_O and the solvent removed under reduced pressure. The residue was dissolved in CH_2_Cl_2_/H_2_O and extracted three times with CH_2_Cl_2_. The combined organic extracts were washed with a saturated aqueous solution of NaCl, dried over MgSO_4_, filtered and concentrated *in vacuo*. The crude product was purified by flash column chromatography (silica gel, 1:9 EtOAc:pet ether) to provide the title compound as a white solid (58 mg, 0.25 mmol, 18%):

**R*_f_*** 0.50 (1:9 EtOAc:pet ether); **m.p.** 46-48 °C; **IR** υ_max_ 1474, 1206, 1073, 768, 604; **^1^H NMR** (400 MHz, CDCl_3_) δ 7.73 (d, *J* = 1.5 Hz, 1H), 7.57 (dd, *J* = 7.9, 1.6 Hz, 1H), 7.44 – 7.35 (m, 2H), 7.28 – 7.17 (m, 2H), 7.17 – 7.09 (m, 1H); **^13^C NMR** (101 MHz, CDCl_3_) δ 159.3 (d, *J* = 248.8 Hz, 1C), 140.1, 134.9, 133.1, 132.6 (d, *J* = 1.1 Hz, 1C), 131.1, 131.0 (d, *J* = 12.7 Hz, 1C), 130.3, 125.4 (d, *J* = 15.5 Hz, 1C), 124.3 (d, *J* = 3.7 Hz, 1C), 117.5, 116.1 (d, *J* = 21.7 Hz, 1C), 113.4; **HRMS** (ESI) calcd for [C_13_H_7_^35^Cl^19^FN + H]^+^: 232.0329, found: 232.0322.

2-Chloro-4-cyanophenyl trifluoromethanesulfonate, 1

To a solution of 3-chloro-4-hydroxybenzonitrile (2.00 g, 13.0 mmol 1.0 equiv) in anhydrous dichloromethane (40.0 mL, 3.00 mM) was added pyridine (3.20 mL, 39.6 mmol 1.6 equiv). The solution was cooled to 0 °C and trifluoromethanesulfonic anhydride (2.84 mL, 16.9 mmol, 1.4 equiv) was added dropwise over 30 minutes. The reaction was allowed to warm to room temperature and stirred overnight. Dichloromethane and excess trifluoroacetic acid were removed under reduced pressure and the residue was diluted with water and extracted with ethyl acetate. The organic layer was washed successively with 10% aqueous hydrochloric acid, 5% aqueous sodium bicarbonate and brine, then dried (MgSO4) and concentrated under reduced pressure. The crude residue was purified by column chromatography on silica gel (5% EtOAc/hexane) to yield the product **4** as a white crystalline solid (3.37 g, 11.8 mmol, 91%).

**R*f*** = 0.68 (20 % EtOAc/hexane); **Mp** = 61-62 °C; **δH** (400 MHz, CDCl3): 7.86 (1H, d, *J* = 2.0 Hz,), 7.68 (1H, dd, *J* = 8.6, 2.0 Hz), 7.51 (1H, d, *J* = 8.6 Hz); **δC** (101 MHz, CDCl3): 148.5, 134.9, 132.2, 128.9, 124.2, 120.1, 116.9, 114.9 (d, *J* = 227.3 Hz); **δF** (376 MHz, CDCl3): -74.0; **νmax**: 2245 (C≡N, m), 1575 (C=C, w), 1478 (C=C, m); **LCMS** Rt = 1.56 min, [M-H]- 284.0.

2-chloro-2’-isopropyl-[1,1’-biphenyl]-4-carbonitrile, 3a

To a solution of aryl triflate (1.6 equiv) and the (2-isopropylphenyl)-boronic acid (50.0 mg, 230 μmol, 1.0 equiv) in anhydrous 1,2-dimethoxyethane (0.16 M) was added 2 M aqueous sodium carbonate (300 μL, 530 μmol, 1.6 equiv). The reaction mixture was degassed by bubbling nitrogen through the solution for 15 minutes before the addition of tetrakis(triphenylphosphine)palladium(0) Pd(PPh3)4 (10.5 mg, 10.0 μmol). The solution was refluxed for 3 hours. The reaction was allowed to cool to room temperature and then diluted with EtOAc and H2O. The aqueous phase was extracted with EtOAc (3×). The combined organic extracts were washed with brine, dried (MgSO4), concentrated under reduced pressure and purified by column chromatography on silica gel (20% EtOAc/hexane) to yieldproduct **6** as a colourless oil (40.0 mg, 150 μmol, 85%).

**R*f*** = 0.25 (20% EtOAc/hexane); **δH** (400 MHz, CD3OD): 7.96 (1H, dd, *J* = 1.6 and 0.3 Hz), 7.76 (1H, dd, *J* = 7.9 and 1.6 Hz), 7.47-7.41 (3H, m), 7.28-7.25 (1H, m,), 7.05 (1H, ddd, *J* = 7.2, 1.8 and 0.7 Hz,), 2.60 (1H, sept, *J* = 6.8 Hz,), 1.22 (3H, d, *J* = 6.7 Hz), 1.09 (3H, d, *J* = 6.7 Hz); **δC** (101 MHz, CD3OD): 147.5, 147.3, 137.9, 135.9, 133.9, 133.7, 131.6, 130.2, 129.9, 126.8, 126.6, 118.4, 113.9, 31.6, 24.7, 23.4; **νmax**: 2962 (=C-H, m), 2326 (C≡N, m), 1473 (CH3, m), 1383 (CH3, m), 832 and 758 (=C-H, s); **HRMS** (ESI+) m/z found [M+H]+ 256.0893, C16H14NCl+ required 256.0893 (Δ 0.0 ppm).

(2-chloro-2’-isopropyl-[1,1’-biphenyl]-4-yl)methanaminium chloride, 3

To a suspension of LiAlH4 (11.4 mg, 300 μmol, 2.0 equiv) in Et2O (3.00 mL, 0.27 M) at 0°C was added AlCl3 (20.0 mg, 150 μmol, 1.0 equiv) and the mixture stirred for 10 minutes. Compound **6** (40.0 mg, 150 μmol, 1.0 equiv) was added and the reaction mixture stirred at room temperature for 30 minutes and then refluxed overnight. The mixture was cooled to 0 °C, diluted with EtOAc and a saturated aqueous solution of potassium sodium tartrate tetrahydrate was added. The suspension was stirred for 1 hour and then poured into a 2 M aqueous solution of Na2CO3. The aqueous phase was extracted with EtOAc (3x). The combined organic phases were washed with brine and dried (MgSO4). The amine (35.0 mg, 130 μmol, 87%, 1.0 equiv) was dissolved in the minimum amount of anhydrous dichloromethane and HCl (0.70 mL, 2 M in 1,4-dioxane, 10 equiv) was added dropwise. The reaction mixture was stirred at room temperature for 1 hour. to yield the hydrochloride salt as a white solid (35.0 mg, 120 μmol, 77%).

**Mp** 218-220°C; **δH** (500 MHz, CD3OD): 7.68 (1H, d, *J* = 1.5 Hz), 7.49 (1H, dd, *J* = 7.8 and 1.5 Hz), 7.44-7.38 (2H, m), 7.35 (1H, d, *J* = 7.8 Hz), 7.24 (1H, td, *J* = 6.9 and 1.7 Hz), 7.03 (1H, dd, *J* = 7.0 and 1.6 Hz), 4.30 (2H, s), 2.65 (1H, sept, *J* = 6.7 Hz), 1.19 (3H, d, *J* = 7.4 Hz), 1.06 (3H, d, *J* = 7.4 Hz); **δC** (126 MHz, CD3OD): 146.3, 141.5, 137.4, 134.1, 133.9, 131.9, 129.6, 128.9, 128.4, 127.1, 125.2, 125.0, 42.1, 30.1, 22.0, 23.4; **νmax**: 2960 (N-H, br), 1478 (CH3, m), 1403 (CH3, m), 834 and 754 (=C-H, s); **HRMS** (ESI+) m/z found [M+H]+ 260.1196 C16H19N2Cl+ required 260.1206 (Δ -3.8 ppm).

2-Chloro-2'-methyl-[1,1'-biphenyl]-4-carbonitrile, 4a

3-chloro-4-bromobenzonitrile (300 mg, 1.39 mmol, 1.0 equiv), *o*-tolylboronic acid (189 mg, 1.39 mmol, 1.0 equiv), Pd(PPh_3_)_4_ (80 mg, 0.07 mmol, 0.05 equiv) and K_2_CO_3_ (383 mg, 2.77 mmol, 2.0 equiv) were weighed into a microwave tube and solvated with DME (3.0 mL) and H_2_O (1.0 mL). The reaction mixture was degassed by bubbling nitrogen through the solution for 5 minutes and then heated to 100 °C under microwave irradiation for 2 hours. The reaction was allowed to cool to room temperature, filtered through celite washing with Et_2_O and the solvent removed under reduced pressure. The residue was dissolved in CH_2_Cl_2_/H_2_O and extracted three times with CH_2_Cl_2_. The combined organic extracts were washed with a saturated aqueous solution of NaCl, dried over MgSO_4_, filtered and concentrated *in vacuo*. The crude product was purified by flash column chromatography (silica gel, 1:9 EtOAc:pet ether) to provide the title compound as a clear oil (300 mg, 1.32 mmol, 95%):

**R*_f_*** 0.56 (2:3 CH_2_Cl_2_:pet ether); **IR** υ_max_ 1473, 1384, 1073, 836, 761, 726, 609; **^1^H NMR** (400 MHz, CDCl_3_) δ 7.77 (d, *J* = 1.5 Hz, 1H), 7.63 (dd, *J* = 7.9, 1.6 Hz, 1H), 7.41 – 7.27 (m, 4H), 7.14 (d, *J* = 7.4 Hz, 1H), 2.16 (s, 3H); **^13^C NMR** (101 MHz, CDCl_3_) δ 145.6, 137.5, 135.5, 134.4, 132.6, 131.8, 130.2, 130.0, 128.7, 128.6, 125.7, 117.3, 112.5, 19.6; **HRMS** (ESI) calcd for [C_14_H_10_^35^ClN + H]^+^: 228.0580, found: 228.0575.

(2-Chloro-2'-methyl-[1,1'-biphenyl]-4-yl)methanamine HCl salt, 4

To a stirred suspension of LiAlH_4_ (77 mg, 2.02 mmol, 2.0 eq) in Et_2_O (3.4 mL) was added AlCl_3_ (269 mg, 2.02 mmol, 2 equiv) and the reaction mixture cooled to 0 °C for 10 minutes. The reaction was allowed to warm to room temperature and the nitrile **4a** (230 mg, 1.01 mmol 1.0 equiv) was added portionwise. The reaction was stirred at room temperature for 30 minutes and then heated at 50 °C overnight. After cooling to room temperature, a saturated aqueous solution of potassium sodium tartrate tetrahydrate and Et_2_O were added and the mixture stirred for 1 hour. The reaction mixture was diluted with 2M aqueous NaCO_3_ and extracted three times with Et_2_O. The combined organic extracts were washed with a saturated aqueous solution of NaCl, dried over MgSO_4_, filtered and concentrated *in vacuo*. The crude product was purified by flash column chromatography (silica gel, 1:5:14 NH_3_:MeOH:CH_2_Cl_2_) to provide free amine. The free amine was then dissolved in CH_2_Cl_2_ (0.5 mL) and HCl (2M in Et_2_O) (5.05 mL, 10.1 mmol) added dropwise. The reaction was stirred for 1 hour before the precipitate was filtered, washed with cold Et_2_O and dried to provide the title compound as a yellow oil (53 mg, 0.20 mmol, 20%):

**HPLC** *t_r_* = 9.24 mins (5-100% B); **IR** υ_max_ 2945, 2832, 1021; **^1^H NMR** (500 MHz, DMSO-d_6_) δ 8.61 (s, 3H), 7.77 (d, *J* = 1.6 Hz, 1H), 7.55 (dd, *J* = 7.8, 1.7 Hz, 1H), 7.37 – 7.30 (m, 3H), 7.29 – 7.24 (m, 1H), 7.08 (d, *J* = 7.3 Hz, 1H), 4.09 (s, 2H), 2.05 (s, 3H); **^13^C NMR** (126 MHz, DMSO-d_6_) δ 139.9, 138.3, 135.4, 135.4, 132.2, 131.3, 129.9, 129.7, 129.1, 128.1, 127.9, 125.8, 41.3, 19.4; **HRMS** (ESI) calcd for [C_14_H_15_N^35^Cl + H]^+^: 232.0893, found: 232.0846.

2-Chloro-2'-methoxy-[1,1'-biphenyl]-4-carbonitrile, 5a

3-chloro-4-bromobenzonitrile (300 mg, 1.39 mmol 1.0 equiv), 2-methoxyphenylboronic acid (211 mg, 1.39 mmol 1.0 equiv), Pd(PPh_3_)_4_ (0.05 equiv) and K_2_CO_3_ (2.0 equiv) were weighed into a microwave tube and solvated with DME (2 mL) and H_2_O (0.6 mL). The reaction mixture was degassed by bubbling nitrogen through the solution for 5 minutes and then heated to 100 °C under microwave irradiation for 2 hours. The reaction was allowed to cool to room temperature, filtered through celite washing with Et_2_O and the solvent removed under reduced pressure. The residue was dissolved in CH_2_Cl_2_/H_2_O and extracted three times with CH_2_Cl_2_. The combined organic extracts were washed with a saturated aqueous solution of NaCl, dried over MgSO_4_, filtered and concentrated *in vacuo*. The crude product was purified by flash column chromatography (silica gel, 1:9 EtOAc:pet ether) to provide the title compound as a white solid (228 mg, 0.94 mmol, 67%):

**R*_f_*** 0.55 (1:4 EtOAc:pet ether); **m.p.** 71.8–72.2 °C; **IR** υ_max_ 2938, 2837, 2359, 2232, 1604, 1503, 1475, 1435, 1384, 1275, 1255, 1234, 1195, 1181, 1163, 1122, 1075, 1050, 1025, 1004, 884, 865, 835, 793, 754; **^1^H NMR** (400 MHz, CDCl_3_) δ 7.75 (d, *J* = 1.6 Hz, 1H), 7.59 (dd, *J* = 7.9, 1.6 Hz, 1H), 7.45 – 7.39 (m, 2H), 7.16 (dd, *J* = 7.5, 1.8 Hz, 1H), 7.05 (td, *J* = 7.5, 1.0 Hz, 1H), 7.00 (d, *J* = 8.3 Hz, 1H), 3.79 (s, 3H); **^13^C NMR** (101 MHz, CDCl_3_) δ 156.5, 143.2, 135.2, 132.8, 132.7, 130.6, 130.5, 130.1, 126.9, 120.7, 117.9, 112.5, 111.2, 55.7; **HRMS** (ESI) calcd for [C_14_H_10_NO^35^Cl + H]^+^: 244.0529, found: 244.0525.

(2-Chloro-2'-methoxy-[1,1'-biphenyl]-4-yl)methanamine HCl salt, 5

To a stirred suspension of LiAlH_4_ (68 mg, 1.8 mmol, 2.0 eq) in Et_2_O (3.0 mL) was added AlCl_3_ (240 mg, 1.8 mmol, 2eq) and the reaction mixture cooled to 0 °C for 10 minutes. The reaction was allowed to warm to room temperature and the nitrile **5a** (220 mg, 0.9 mmol, 1.0 equiv) was added portionwise. The reaction was stirred at room temperature for 30 minutes and then heated at 50 °C overnight. After cooling to room temperature, a saturated aqueous solution of potassium sodium tartrate tetrahydrate and Et_2_O were added and the mixture stirred for 1 hour. The reaction mixture was diluted with 2M aqueous NaCO_3_ and extracted three times with Et_2_O. The combined organic extracts were washed with a saturated aqueous solution of NaCl, dried over MgSO_4_, filtered and concentrated *in vacuo*. The crude product was purified by flash column chromatography (silica gel, 1:5:14 NH_3_:MeOH:CH_2_Cl_2_) to provide free amine. The free amine was then dissolved in CH_2_Cl_2_ (0.5 mL) and HCl (2M in Et_2_O) (4.5 mL, 9.0 mmol) added dropwise. The reaction was stirred for 1 hour before the precipitate was filtered, washed with cold Et_2_O and dried to provide the title compound as a white solid (65 mg, 0.23 mmol, 26%):

**R*_f_*** 0.15 (1:10 MeOH:CH_2_Cl_2_); **m.p.** 194-196 °C; **IR** υ_max_ 3300 – 2300, 2890, 2591, 1599, 1582, 1505, 1479, 1463, 1434, 1397, 1378, 1275, 1254, 1232, 1214, 1121, 1075, 1049, 1026, 1003; **^1^H NMR** (500 MHz, DMSO-d_6_) δ 8.37 (s, 3H), 7.68 (d, *J* = 1.6 Hz, 1H), 7.47 (dd, *J* = 7.8, 1.7 Hz, 1H), 7.41 (ddd, *J* = 8.3, 7.4, 1.8 Hz, 1H), 7.35 (d, *J* = 7.8 Hz, 1H), 7.12 (dd, *J* = 7.5, 1.8 Hz, 2H), 7.03 (td, *J* = 7.4, 1.0 Hz, 1H), 4.08 (s, 2H), 3.72 (s, 3H); **^13^C NMR** (126 MHz, DMSO-d_6_) δ 156.3, 137.5, 135.1, 132.9, 131.9, 130.4, 129.8, 129.5, 127.6, 127.2, 120.3, 111.4, 55.4, 41.5; **HRMS** (ESI) calcd for [C_14_H_14_NO^35^Cl + H]^+^: 248.0842, found: 248.0834.

3-chloro-5-(1H-indol-4-yl)benzonitrile, 7a

To a solution of the appropriate boronic acid (250 mg, 1.55 mmol, 1.5 equiv) and the appropriate aryl bromide (167 mg, 1.03 mmol, 1.0 equiv) in 1,4-dioxane (3.50 mL) was added 1.75 M aqueous K3PO4 solution (1.00 mL, 1.7 equiv). The mixture was degassed by bubbling nitrogen for 10 minutes before PCy3 (7.00 mg, 20.0 μmol, 0.024 equiv) and Pd2(dba)3 (9.50 mg, 10.0 μmol, 0.010 equiv) were added. The mixture was refluxed for 6 hours. The crude mixture was diluted with EtOAc and filtered through Celite. The organic phase was washed with water and then dried (MgSO4), the solvent evaporated and the product purified by column chromatography on aluminium oxide gel with (0 to 20% EtOAc/hexane). The reaction yielded **24** as white solid (155 mg, 570 μmol, 55%).

**R*f*** = 0.24 (20% EtOAc/hexane); **Mp** = 134-137 °C; **δH** (500 MHz, CDCl3): 8.42 (1H, br s, NH), 7.92 (1H, t, *J =* 1.6 Hz, H3), 7.87 (1H, t, *J =* 1.5 Hz, H5), 7.63 (1H, t, *J =* 1.6 Hz, H1), 7.48 (1H, d, *J =* 8.2 Hz, H15), 7.33-7.24 (2H, m, H11, 14), 7.15 (1H, d, *J =* 8.2 Hz, H13), 6.32-6.28 (1H, m, H10); **δC** (125 MHz, CDCl3): 144.4 (C2), 136.3 (C12), 135.3 (C4), 133.2 (C3), 130.5 (C9), 130.4 (C5), 129.9 (C1), 125.7 (C8), 125.5 (C14), 122.4 (C11), 120.0 (C15), 117.8 (C7), 113.9 (C6), 111.9 (C13), 101.2 (C10); **νmax:** 3429 (N-H, m), 2236 (C≡N, w), 1566 (C=C, m), 879, 795, 752, (=C-H, s); **HRMS** (ESI+) m/z found [M+H]+ 253.0524, C15H10N2Cl+ required 253.0527 (Δ -1.0 ppm).

(3-chloro-5-(1H-indol-4-yl)phenyl)methanamine, **7**

To a suspension of LiAlH4 (22.0 mg, 570 μmol, 2.0 equiv) in Et2O (5.25 mL, 0.27 M) at 0°C was added AlCl3 (38.0 mg, 285 μmol, 1.0 equiv) and the mixture stirred for 10 minutes. Compound **24** (77.5 mg, 280 μmol 1.0 equiv) was added and the reaction mixture stirred at room temperature for 30 minutes and then refluxed overnight. The mixture was cooled to 0 °C, diluted with EtOAc and a saturated aqueous solution of potassium sodium tartrate tetrahydrate was added. The suspension was stirred for 1 hour and then poured into a 2 M aqueous solution of Na2CO3. The aqueous phase was extracted with EtOAc (3x). The combined organic phases were washed with brine and dried (MgSO4). The crude mixture was purified by flash column chromatography on aluminium oxide (hexane: EtOAc: NH3 (80:18:2)). The desired product was obtained as a white film (57.0 mg, 220 μmol, 79%).

**R*f*** = 0.40 [Hexane:EtOAc:NH3 (7:8.2:0.2)]. **δH** (500 MHz, CD3OD): 7.58-7.53 (2H, m, H3, 5), 7.44 (1H, td, *J =* 8.1 and 0.8 Hz, H15), 7.36 (1H, t, *J =* 1.7 Hz, H1), 7.31 (1H, d, *J* = 3.2 Hz, H11), 7.21-7.17 (1H, m, H14), 7.10 (1H, dd, *J =* 7.4 and 0.8 Hz, H13), 6.60 (1H, dd, *J =* 3.2 and 0.8 Hz, H10), 3.81 (2H, s, H7); **δC** (126 MHz, CD3OD): 144.5 (C6), 143.9 (C2), 136.8 (C12), 133.9 (C4), 132.1 (C9), 126.9 (C3), 125.9 (C5), 125.8 (C1), 125.3 (C14), 125.0 (C11), 121.2 (C15), 118.6 (C13), 110.7 (C8), 99.8 (C10), 44.6 (C7); **νmax:** 3437 (N-H, m), 679 (=C-H, s). **HRMS** (ESI+) m/z found [M+H]+ 257.0831, C15H13N2Cl+ required 257.0840 (Δ -3.4 ppm).

All compounds found in SI_3

1-(3,4-dichlorophenyl)but-3-en-1-amine

A solution of 3,4-dichlorobenzonitrile (250 mg, 1.45 mmol) in diethyl ether (5 cm^3^) was cooled to 0°C and treated with lithium triethylborohydride in tetrahydrofuran (1M, 1.45 mL, 1.45 mmol) The solution was stirred for 2 hr before methanol (1 L) was added and the mixture stirred for 30 min. The solvent was removed *in vacuo* and the residue co-evaporated with pentane (2 x 30 mL). The solid residue was dissolved in diethyl ether (6 mL) and the solution was cooled to -78°C. Allylmagnesium bromide diethyl ether (1M, 2.25 mL, 2.25 mmol) was added. The reaction was stirred at -78°C for 21 hr and then warmed to rt. Methanol (1 mL) and NaOH (3M, 3 mL) were added sequentially and the reaction mixture was extracted with diethyl ether (30 mL) and NaOH (3M, 30 mL). The aqueous layer was further extracted with diethyl ether (2 x 30 mL) and the combined organic layers were dried over magnesium sulfate and concentrated *in vacuo*. The crude product was purified by flash column chromatography eluting with a gradient of ethyl acetate: hexane (20:80 → 40:60) to yield the product as a pale yellow oil (187 mg, 60%).

**Rf** 0.36 (SiO_2_; 1:1 ethyl acetate: hexane); **1H NMR** (500MHz, d_6_-DMSO): *δ* = 7.59 (1H, d, *J* = 2.0 Hz), 7.52 (1H, d, *J* = 8.3 Hz), 7.30 (1H, dd, *J* = 8.3, 2.0 Hz), 5.74-5.65 (1H, m), 4.98-4.93 (2H, m), 3.87 (1H, t, *J* = 6.8 Hz), 2.26 (2H, t, *J* = 6.7 Hz), 3.31 (1H, s); **13C NMR** (126MHz, d6-DMSO): *δ* = 148.4, 135.7, 130.7, 130.1, 128.6, 128.6, 127.1, 117.2, 54.3, 43.8; **IR** υ*max* /cm-1 = 3079 (w, N-H), 2979 (w, C-H), 2907 (w, C-H), 1639 (m, N-H), 1466 (s, C=C); **HRMS** (ESI): m/z found [M+H]+ 216.0347, C_10_H_12_NCl_2_ requires 216.0347.

4-amino-4-(3,4-dichlorophenyl)butan-1-ol

A solution of 3,4-dichlorobenzonitrile (516 mg, 3.00 mmol) in diethyl ether (6 mL) was cooled to 0°C. The solution was treated with DIBAL-H in hexane (1M, 3.15 mL, 3.15 mmol) and stirred for 1 hr. The solution was cooled to -78°C and allylmagnesium bromide in diethyl ether (1M, 4.5 mL, 4.50 mmol) was added. The reaction was stirred at -78°C for 21 hr and then warmed to 0°C. Borane dimethyl sulfide complex (10M, 0.45 mL, 4.50 mmol) and tetrahydrofuran (6 mL) were added and the mixture stirred at rt for 24 hr. The solution was cooled to 0°C and methanol (0.6 mL), NaOH (3M, 3 mL) and hydrogen peroxide (30%, 3 mL) were added sequentially. The reaction mixture was extracted with diethyl ether (30 mL) and NaOH (3M, 30 mL). The aqueous layer was further extracted with diethyl ether (2 x 30 mL) and the combined organic layers were dried over magnesium sulfate and concentrated *in vacuo*. The crude product was purified by flash column chromatography eluting with a gradient of dichloromethane: methanol (95:5 → 85:15) to yield the product as a white solid (115 mg, 16%).

**R_f_** 0.33 (SiO2; 85:15 dichloromethane: methanol); **m.p.** 61.7-64.0°C; **1H NMR** (400MHz, CDCl3): *δ* = 7.39-7.37 (2H, m), 7.13 (1H, dd, *J* = 8.3, 2.0 Hz), 3.90-3.87 (1H, m), 3.68-3.59 (2H, m), 1.86-1.58 (4H, m); **13C NMR** (126MHz, d6-DMSO): *δ* = 148.6, 130.7, 130.2, 128.7, 128.6, 127.1, 60.8, 54.5, 35.7, 29.4; **IR** υ*max* /cm^-1^ = 3331 (m, N-H), 3278 (m, N-H), 3085 (m, br, O-H), 2923 (m, C-H), 2857 (m, C-H), 1613 (m, N-H), 1469 (m, C=C); **HRMS** (ESI): m/z found [M+H]+ 234.0457, C10H14NOCl2 requires 234.0452.

1-(3,4-dichlorophenyl)pentane-1,5-diol

A solution of 1-(3,4-dichlorophenyl)pent-4-en-1-ol (400 mg, 1.73 mmol) in tetrahydrofuran (8 mL) was cooled to 0°C and treated with borane-tetrahydrofuran complex (1M, 0.90 mL, 0.90 mmol). The solution was stirred for 4 hr at rt and then cooled to 0°C. The reaction was quenched with a few drops of water, followed by the sequential addition of NaOH (3M, 1 mL) and hydrogen peroxide (30%, 1 mL). The mixture was refluxed for 1 hr, and then cooled to rt. Water (20 mL) was added and the reaction mixture extracted with diethyl ether (20 mL). The aqueous layer was further extracted with diethyl ether (2 x 30 mL) and the combined organic layers were dried over magnesium sulfate and concentrated *in vacuo*. The crude product was purified by flash column chromatography eluting with dichloromethane: methanol (96:4) to yield the product as a clear oil (173 mg, 40%).

**R_f_** 0.54 (SiO2; 80:10 dichloromethane: methanol); **^1^H NMR** (500MHz, CDCl3): *δ* = 7.45 (1H, d, *J* = 2.0 Hz), 7.41 (1H, d, *J* = 8.3 Hz), 7.17 (1H, dd, *J* = 8.3, 2.0 H), 4.67 (1H, dd, *J* = 7.6, 5.4 Hz), 3.65 (2H, t, *J* = 6.4 Hz), 2.07-1.96 (4H, m), 1.63-1.57 (2H, m); **13C NMR** (126MHz, d6-DMSO): *δ* = 147.9, 130.7, 130.2, 128.9, 127.9, 126.3, 71.1, 60.7 39.0, 32.5, 21.8; **IR** υ*max* /cm-1 = 3319 (s, br, O-H), 2938 (m, C-H), 2861 (m, C-H), 1467 (s, C=C); **HRMS** (ESI): m/z found [M+K]+ 287.0000, C_11_H_14_O_2_Cl_2_K requires 287.0013.

2-(3,4-dichlorophenyl)-4,5-dihydro-1H-imidazole

A solution of 3,4-dichlorobenzaldehyde (400 mg, 2.29 mmol) in anhydrous dichloromethane (20 mL) was treated with ethylenediamine (145 mg, 2.40 mmol) at 0°C and the mixture stirred for 30 min. *N*-bromosuccinimide (427mg, 2.40mmol) was added, and the solution was left to stir for 18 hr at rt. The reaction mixture was quenched using 10% NaOH (30 mL) and extracted with dichloromethane (30 mL). The aqueous layer was further extracted with dichloromethane (2 x 30 mL) and the combined organic layers were dried over sodium sulfate and concentrated *in vacuo*. The crude product was purified by flash column chromatography eluting with a gradient of dichloromethane: methanol (95:5 → 85:15) to yield the product as a white solid (409 mg, 83%).

**R_f_** 0.60 (SiO2; 85:15 dichloromethane: methanol); **m.p.** 129.0-130.7°C; **^1^H NMR** (400MHz, CDCl3): *δ* = 7.87 (1H, d, *J* = 2.0 Hz), 7.59 (1H, dd, *J* = 8.3, 2.0 Hz), 7.46 (1H, d, *J* = 8.3 Hz), 3.78 (4H, s); **13C NMR** (101MHz, CDCl3): *δ* = 162.7, 134.8, 132.8, 130.5, 130.4, 129.0, 126.1, 77.2; **IR** υ*max* /cm^-1^ = 3191 (w, N-H), 2850 (w, C-H), 1606 (w, C=C), 1552 (m, C=C); **HRMS** (ESI): m/z found [M+H]+ 215.0143, C9H9N2Cl2 requires 215.0151.

1-(3,4-dichlorophenyl)pent-4-en-1-ol

A solution of 4-bromo-but-1-ene (527 mg, 3.90 mmol) in diethyl ether (2 mL) was added dropwise to a mixture of magnesium turnings (219 mg, 9.01 mmol) in diethyl ether (3 mL). The solution was stirred for 1 hr at rt and then heated to reflux for 30 min. A solution of 3,4-dichlorobenzaldehyde (600 mg, 3.43 mmol) in diethyl ether (3 mL) was added dropwise and the solution left to reflux for 2 hr before being cooled to rt. Ice (10 ml) was added and the mixture dissolved in HCl (2M, 10 mL) and the organic layer extracted with diethyl ether. The aqueous layer was further extracted with diethyl ether (2 x 30 mL) and the combined organic layers were dried over magnesium sulfate and concentrated *in vacuo*. The crude product was purified by flash column chromatography eluting with dichloromethane: methanol (95:5) to yield the product as a yellow oil (602 mg, 76%).

**R_f_** 0.79 (SiO2; 1:1 ethyl acetate: hexane); **^1^H NMR** (500MHz, d_6_-DMSO): *δ* = 7.56-7.53 (2H, m), 7.30-7.28 (1H, m), 5.84-5.75 (1H, m), (1H, s), 5.00-4.90 (2H, m), 4.54 (1H, dd, *J* = 6.2, 10.9 Hz), 2.07-1.96 (2H, m), 1.66-1.59 (2H, m); **^13^C NMR** (126MHz, d_6_-DMSO): *δ* = 147.6, 138.4, 130.8, 130.3, 129.0, 127.8, 126.2, 114.9, 70.5, 38.1, 29.4; **IR** υ*max* /cm-1 = 3361 (m, br, O-H), 2978 (w, C-H), 2934 (m, C-H), 1468 (s, C=C); **HRMS** (ESI): m/z found [M+H]+ 231.0343, C11H13OCl2 requires 231.0338.

2-(1-((3,4-Dichlorobenzyl)amino)ethyl)butane-1,4-diol

α-Acetylbutyrolactone (475 mg, 3.71 mmol) in dichloroethane (10 mL) was treated with 3,4-dichlorobenzylamine (981 mg, 5.57 mmol), and sodium triacetoxyborohydride (1.10 g, 5.19 mmol). The residue was dissolved in diethyl ether (20 mL) and added dropwise to a solution of lithium aluminium hydride (550 mg, 14.5 mmol) in diethyl ether (5 mL). The reaction mixture was stirred for 18 hr at rt and then quenched with slow dropwise addition of water (10 mL), followed by NaOH (1M, 10 mL). The organic layer was extracted and then the aqueous layer further extracted with diethyl ether (2 x 30 mL). The combined organic layers were dried over magnesium sulfate and concentrated *in vacuo*. The crude product was purified by flash column chromatography eluting with dichloromethane: methanol (98:2) to yield the product as a clear oil (247 mg, 23%).

**R_f_** 0.45 (SiO_2_; 90:10 dichloromethane: methanol); **^1^H NMR** (500 MHz, DMSO): *δ* = 7.59 (1H, s), 7.52 (1H, d, *J* = 8.2 Hz), 7.29 (1H, d, *J* = 8.2 Hz), 5.42 (1H, q, *J* = 6.8 Hz), 3.79 (2H, s), 3.68 (2H, d, *J* = 4.9 Hz), 3.38 (2H, t, *J* = 7.4 Hz), 2.17 (2H, t, *J* = 7.4 Hz), 1.56 (2H, d, *J* = 6,8 Hz), 1.01-0.97 (1H, m); **^13^C NMR** (126MHz, DMSO): *δ* = 145.8, 130.8, 130.2, 129.0, 128.5, 127.4, 119.7, 65.5, 59.7, 51.5, 31.7, 15.1, 12.8; **IR** υ*_max_* /cm^-1^ = 3281 (m, O-H), 3046 (m, br, O-H), 2890 (m, C-H), 1621 (m, N-H), 1559 (m, C=C), 1468 (m, C=C); **HRMS** (ESI): m/z found [M+H]^+^ 292.0862, C_13_H_20_NO_2_Cl_2_ requires 292.0866.

1-(3,4-dichlorophenyl)-N-methylmethanamine

3,4-dichlorobenzaldehyde (600 mg, 3.43 mmol, 1.0 equiv) and the methylamine (0.64 ml, 5.14 mmol, 8.03 M in ethanol, 1.5 equiv) were combined in anhydrous 1,2-dichloroethane (12 ml) under an atmosphere of nitrogen and stirred for 3 hours. sodium triacetoxyborohydride (1.02 g, 4.81 mmol, 1.4 equiv) was added and the reaction was stirred at room temperature for 18 hours. The reaction mixture was poured into 2M aqueous sodium carbonate and extracted with ethyl acetate (3×10mL). The combined organic extracts were dried (MgSO4), concentrated under reduced pressure and purified by column chromatography on silica eluting with a stepped gradient of 7N NH3 in methanol: methanol: dichloromethane (1:5:94 → 1:10:89 → 1:15:84) to yield the product as a pale yellow oil (455 mg, 70%).

**R*_f_*** = 0.21 [7N NH3 in methanol: methanol: dichloromethane (1:5:94)]. **δ_H_ /ppm** (500 MHz, CDCl3): 7.43 (1H, d, *J*=2.0 Hz), 7.38 (1H, d, *J*=8.2 Hz), 7.16 (1H, dd, *J*=8.2 and 2.0 Hz), 3.71 (2H, s), 2.44 (3H, s). **δ_C_ /ppm** (125 MHz, CDCl3): 140.6, 132.5, 130.9, 130.4, 130.2, 127.6, 55.0, 36.1. **ν_max_ /cm^-1^:** 3297 (w, N-H), 2793 (m, C-H), 1564 (w, C=C), 1470 (vs, C=C). **HRMS** (ESI+) *m/z* found [M+H]+ 190.0177, C_8_H_10_NCl_2_+ required 190.0185 (Δ -4.2 ppm).

(1s,4s)-4-((3,4-dichlorobenzyl)amino)cyclohexan-1-ol

3,4-dichlorobenzaldehyde (400 mg, 2.29 mmol, 1 eq) in methanol (8 mL) was treated with the *cis*-4-aminocyclohexanol hydrochloride (522 mg, 3.44 mmol, 1.5 eq) and triethylamine (0.5 cm3, 3.58 mmol, 2 eq), and the mixture stirred at rt for 3 hr. Sodium triacetoxyborohydride (680 mg, 3.21 mmol 1.4 eq) was then divided into two portions and added to the solution at half-hour intervals, and the solution was left to stir for 18 hr. The reaction mixture was quenched using NaOH (2M, 30 mL) and extracted with dichloromethane (30 mL). The aqueous layer was further extracted with dichloromethane (2 x 30 mL) and the combined organic layers were dried over magnesium sulfate, concentrated *in vacuo* and purified by column chromatography eluting with dichloromethane: methanol (95:5) to yield the product as an off-white solid (366 mg, 58%).

**R_f_** 0.76 (SiO2; 85:15 dichloromethane: methanol); **m.p.** 64.1-65.9°C; **^1^H NMR** (500MHz, d_6_-DMSO): *δ* = 7.58 (1H, d, *J* = 1.9 Hz), 7.52 (1H, d, *J* = 8.2 Hz), 7.31 (1H, dd, *J* = 8.2, 1.9 Hz), 4.26 (1H, d, *J* = 3.6 Hz), 3.67 (2H, s), 3.59-3.55 (1H, m), 2.40-2.35 (2H, m), 2.02 (1H, s), 1.58-1.52 (2H, m), 1.49-1.39 (4H, m), 1.38-1.32 (2H, m); **^13^C NMR** (126MHz, d_6_-DMSO): *δ* = 143.4, 130.7, 130.2, 129.7, 128.7, 128.2, 65.7, 53.3, 48.8, 30.9, 27.5; **IR** υ*max* /cm^-1^ = 3323 (w, N-H), 3258 (m, br, O-H), 2928 (m, C-H), 2862 (w, C-H), 1472 (m, C=C); **HRMS** (ESI): m/z found [M+H]+ 274.0753, C_13_H_18_NOCl_2_ requires 274.0760.

1-(2-((3,4-dichlorobenzyl)amino)ethyl)imidazolidin-2-one

A solution of the 3,4-dichlorobenzaldehyde (400 mg, 2.29 mmol, 1 eq) in 1,2-dichloroethane (8 cm3) was treated with the 1-(2-aminoethyl)imidazoline-2-one (444 mg, 3.44 mmol, 1.5 eq) and the mixture stirred at rt for 3 hr. Sodium triacetoxyborohydride (680 mg, 3.21 mmol, 1.4 equivalents) was then divided into two portions and added to the solution at half-hour intervals, and the solution was left to stir for 18 hr. The reaction mixture was quenched using saturated sodium bicarbonate solution (30 cm3) and extracted with ethyl acetate (30 cm3). The aqueous layer was further extracted with ethyl acetate (2 x 30 cm3) and the combined organic layers were dried over magnesium sulfate, concentrated *in vacuo* and purified by column chromatography on aluminium oxide eluting with dichloromethane: methanol (98:2) to yield the product as a white solid (287 mg, 43%).

**R_f_** 0.37 (Al2O3; 90:10 dichloromethane: methanol); **m.p.** 78.3-79.3°C; ^1^**H NMR** (500MHz, d6-DMSO): *δ* = 7.57 (1H, d, *J* = 1.9 Hz), 7.54 (1H, d, *J* = 8.3 Hz), 7.29 (1H, dd, *J* = 8.3, 1.9 Hz), 6.22 (1H, s) 3.67 (2H, s), 3.30-3.28 (2H, m), 3.19-3.16 (2H, m), 3.09 (2H, t, *J* = 6.5 Hz), 2.54 (2H, t, *J* = 6.5 Hz); ^13^**C NMR** (126MHz, d6-DMSO): *δ* = 162.4, 142.5, 130.8, 130.3, 129.8, 128.9, 128.3, 51.2, 46.5, 45.0, 43.0, 37.5; **IR** υ*max* /cm^-1^ = 3319 (w, N-H), 3242 (m, N-H), 2873 (m, C-H), 2824 (m, C-H), 1687 (s, C=O), 1490 (m, C=C); **HRMS** (ESI): m/z found [M+H]+ 288.0655, C12H16N3OCl2 requires 288.0655.

(5-(((3,4-dichlorobenzyl)amino)methyl)furan-2-yl)methanol

A solution of 5-(hydroxymethyl)furfural (300 mg, 2.38 mmol) in methanol (10 mL) was treated with 3,4-dichlorobenzylamine (539 mg, 3.06 mmol), and the mixture stirred at rt for 5 hr. Sodium borohydride (158 mg, 4.18 mmol) was then added, and the solution was left to stir for 1 hr. The methanol was removed *in vacuo* and the residue dissolved in water (30 mL) extracted with dichloromethane (30 mL). The aqueous layer was further extracted with dichloromethane (2 x 30 mL) and the combined organic layers were dried over magnesium sulfate and concentrated *in vacuo*. The crude product was purified by flash column chromatography on aluminium oxide eluting with dichloromethane: methanol (98:2) to yield the product as an orange oil (635 mg, 93%).

**Rf** 0.70 (Al2O3; 95:5 dichloromethane: methanol); ^1^**H NMR** (500MHz, d6-DMSO): *δ* = 7.58-7.51 (2H, m), 7.29 (1H, d, *J* = 8.3 Hz), 6.15 (1H, d, *J* = 2.9 Hz), 6.13 (1H, d, *J* = 2.9 Hz), 5.11 (1H, s), 4.32 (1H, s), 3.66 (2H, s). 3.58 (2H, s); ^13^**C NMR** (126MHz, d6-DMSO): *δ* = 154.4, 153.4, 145.8, 130.8, 130.3, 130.2, 129.8, 128.3, 107.5, 107.4, 55.8, 50.7, 44.9); **IR** υ*max* /cm-1 = 3289 (m, br, O-H), 2831 (m, C-H), 1563 (w, C=C), 1470 (m, C=C); **HRMS** (ESI): m/z found [M+H]+ 286.0387, C13H14NO2Cl2 requires 286.0396.

N^1^-(3,4-dichlorobenzyl)-N^3^,N^3^-dimethylpropane-1,3-diamine

3,4-dichlorobenzaldehyde (300 mg, 1.71 mmol 1.0 eq) and the (dimethylamino)-1-propylamine (0.323 ml, 2.57 mmol 1.5 eq) were combined in anhydrous 1,2-dichloroethane (6 ml) under an atmosphere of nitrogen and stirred for 3 hours. Sodium triacetoxyborohydride (509 mg, 2.40 mmol, 1.4 eq) was added and the reaction was stirred at room temperature for 18 hours. The reaction mixture was poured into 2M aqueous sodium carbonate and extracted with ethyl acetate (3 × 10mL). The combined organic extracts were dried (MgSO4), concentrated under reduced pressure and purified by column chromatography on silica eluting with 2N NH_3_ in methanol: methanol: dichloromethane (1:15:84) to yield the product **165** as a clear oil (317 mg, 71%).

**R*_f_*** = 0.18 [methanol: dichloromethane (50:50)]. **δ_H_ /ppm** (400 MHz, CDCl_3_): 7.43 (1H, d, *J*=2.0 Hz), 7.37 (1H, d, *J*=8.2 Hz), 7.15 (1H, dd, *J* = 8.2 and 2.0 Hz), 3.74 (2H, s), 2.64 (2H, t, *J*=6.9 Hz), 2.36-2.28 (2H, m), 2.22 (6H, s), 1.73-1.62 (2H, m).mmm **δ_C_ /ppm** (101 MHz, CDCl_3_): 141.1, 132.5, 130.8, 130.4, 130.1, 127.5, 58.2, 53.0, 48.0, 45.7, 28.1. **ν_max_ /cm^-1^:** 3277 (w, N-H), 2943 (s, C-H), 2816 (s, C-H), 1565 (w, C=C), 1468 (vs, C=C). **HRMS** (ESI+) *m/z* found [M+H]+ 261.0913, C_12_H_19_N_2_Cl_2_+ required 261.0920 (Δ -2.5 ppm).

N-(3,4-dichlorobenzyl)propan-1-amine

3,4-dichlorobenzaldehyde (300 mg, 1.71 mmol 1.0 eq) and the propylamine (0.21 mL, 2.6 mmol 1.5 eq) were combined in anhydrous 1,2-dichloroethane (6 mL) under an atmosphere of nitrogen and stirred for 3 hours. Sodium triacetoxyborohydride (509 mg, 2.40 mmol 1.4 eq) was added and the reaction was stirred at room temperature for 18 hours. The reaction mixture was poured into 2M aqueous sodium carbonate and extracted with ethyl acetate (3 × 10mL). The combined organic extracts were dried (MgSO_4_), concentrated under reduced pressure and purified by column chromatography on silica eluting with a stepped gradient of 2N NH_3_ in methanol: ethyl acetate: hexane (1:10:89 → 1:30:69 → 1:50:49) to yield the product as a pale yellow oil (268 mg, 72%).

**R*_f_*** = 0.44 [2N NH_3_ in methanol: ethyl acetate: hexane (1:50:49)]. **δ_H_ /ppm** (400 MHz, *d*_6_- DMSO): 7.58 (1H, d, *J*=1.9 Hz), 7.55 (1H, d, *J*=8.2 Hz), 7.31 (1H, dd, *J*=8.2 and 2.0 Hz), 3.66 (2H, s), 2.40 (2H, t, *J*=7.1 Hz), 1.51-1.27 (2H, m), 0.85 (3H, t, *J*=7.4 Hz). **δ_C_ /ppm** (101 MHz, *d*6-DMSO): 142.8, 130.7, 130.1, 129.6, 128.7, 128.1, 51.6, 50.5, 22.6, 11.8. **ν_max_ /cm^-1^:** 3289 (w, N-H), 2960 (s, C-H), 1563 (w, C=C), 1470 (vs, C=C). **HRMS** (ESI+) *m/z* found [M+H]+ 218.0491, C_10_H_14_NCl_2_+ required 218.0498 (Δ -2.9 ppm).

N-(3,4-dichlorobenzyl)-3-(1H-imidazol-1-yl)propan-1-amine

3,4-dichlorobenzaldehyde (400 mg, 2.29 mmol 1 eq) in 1,2-dichloroethane (8 mL) was treated with the 1-(3- aminopropyl)imidazole (431 mg, 3.44 mmol, 1.5 eq) and the mixture stirred at rt for 3 hr. Sodium triacetoxyborohydride (680 mg, 3.21 mmol, 1.4 eq) was then divided into two portions and added to the solution at half-hour intervals, and the solution was left to stir for 18 hr. The reaction mixture was quenched using saturated sodium bicarbonate solution (30 mL) and extracted with ethyl acetate (30 mL). The aqueous layer was further extracted with ethyl acetate (2 x 30 mL) and the combined organic layers were dried over magnesium sulfate, concentrated *in vacuo* and purified by column chromatography on aluminium oxide eluting with dichloromethane: methanol (98:2) to yield the product as a pale yellow oil (306 mg, 47%).

**R_f_** 0.74 (Al_2_O_3_; 90:10 dichloromethane: methanol); **^1^H NMR** (400MHz, CDCl_3_): *δ* = 7.95 (1H, s), 7.42 (1H, d, *J* = 1.8 Hz), 7.38 (1H, d, *J* = 8.2 Hz), 7.14 (1H, dd, *J* = 8.2, 1.8 Hz), 7.07 (1H, s), 6.94 (1H, s), 4.11-4.03 (2H, m), 3.71 (2H, s), 3.55 (1H, t, *J* = 6.7 Hz), 2.59 (1H, t, *J* = 6.7 Hz), 2.16 (1H, t, *J* = 6.7 Hz), 1.95 (1H, t, *J* = 6.7 Hz); **^13^C NMR** (101MHz, CDCl_3_): *δ* = 134.4, 134.0, 131.0, 130.6, 130.2, 129.9, 128.1, 127.4, 113.8, 47.5, 40.4, 39.6, 26.7; **IR** υ*max* /cm^-1^ = 2937 (w, C-H), 2841 (w, C-H), 1706 (m, C=N), 1646 (m, C=C), 1508 (m, C=C); **HRMS** (ESI): m/z found [M+H]+ 284.0721, C_13_H_16_N_3_Cl_2_ requires 284.0709.

3-((3,4-dichlorobenzyl)amino)propan-1-ol

3,4-dichlorobenzaldehyde (300 mg, 1.71 mmol 1.0 eq) and the 3-amino-1-propanol (0.196 ml, 2.57 mmol 1.5 eq) were combined in anhydrous 1,2-dichloroethane (6 mL) under an atmosphere of nitrogen and stirred for 3 hours. Sodium triacetoxyborohydride (509 mg, 2.40 mmol, 1.4 eq) was added and the reaction was stirred at room temperature for 18 hours. The reaction mixture was poured into 2M aqueous sodium carbonate and extracted with ethyl acetate (3 × 10 mL). The combined organic extracts were dried (MgSO_4_), concentrated under reduced pressure and purified by column chromatography on silica eluting with a stepped gradient of 2N NH_3_ in methanol: methanol: dichloromethane (1:5:94 → 1:10:89) to yield the product as a clear oil (329 mg, 82%).

**R*_f_*** = 0.20 [2N NH_3_ in methanol: methanol: dichloromethane (1:5:94)]. **δ_H_ /ppm** (400 MHz, CDCl_3_): 7.41-7.37 (2H, m), 7.15 (1H, dd, *J*=8.2 and 2.0 Hz), 3.82-3.78 (2H, m), 3.75 (2H, s), 2.89-2.83 (2H, m), 2.40 (2H, br s), 1.77-1.69 (2H, m). **δ_C_ /ppm** (101 MHz, CDCl_3_): 140.0, 132.6, 131.3, 130.6, 130.2, 127.6, 64.2, 53.0, 49.3, 31.1. **ν_max_ /cm^-1^:** 3298 (s, O-H), 2839 (s, C-H), 1563 (w, C=C), 1470 (vs C=C). **HRMS** (ESI+) *m/z* found [M+H]+ 234.0441, C_10_H_14_NOCl_2_+ required 234.0447 (Δ -2.8 ppm).

4-((3,4-dichlorobenzyl)amino)butan-1-ol

A solution of the 3,4-dichlorobenzaldehyde (400 mg, 2.29 mmol 1 eq) in 1,2-dichloroethane (8 mL) was treated with the 4- amino-1-butanol (307 mg, 3.44 mmol 1.5 eq) and the mixture stirred at rt for 3 hr. Sodium triacetoxyborohydride (680 mg, 3.21 mmol 1.4 eq) was then divided into two portions and added to the solution at half-hour intervals, and the solution was left to stir for 18 hr. The reaction mixture was quenched using saturated sodium bicarbonate solution (30 mL) and extracted with ethyl acetate (30 mL). The aqueous layer was further extracted with ethyl acetate (2 x 30 mL) and the combined organic layers were dried over magnesium sulfate, concentrated *in vacuo* and purified by column chromatography eluting with a gradient of dichloromethane: methanol (95:5 → 85:15) to yield the product as a pale yellow oil (334 mg, 59%).

**R_f_** 0.34 (SiO_2_; 95:5 dichloromethane: methanol); **^1^H NMR** (400 MHz, CDCl3): *δ* = 7.40-7.38 (2H, m), 7.17 (1H, dd, *J* = 8.2, 2.0 Hz), 3.74 (2H, s), 3.59 (2H, t, *J* = 5.6 Hz), 2.68 (2H, t, *J* = 5.6Hz), 1.69-1.62 (4H, m); **^13^C NMR** (101MHz, CDCl3): *δ* = 139.4, 132.5, 131.3, 130.6, 130.3, 127.7, 62.7, 52.7, 49.2, 32.0, 28.2; **IR** υ*max* /cm^-1^ = 3308 (m, br, O-H), 2933 (m, C-H), 2858 (m, C-H), 1561 (w, C=C), 1470 (s, C=C); **HRMS** (ESI): m/z found [M+H]+ 248.0609, C_11_H_16_NOCl_2_ requires 248.0609.

(4-((3,4-dichlorobenzyl)amino)phenyl)methanol

A solution of the 3,4-dichlorobenzaldehyde (400 mg, 2.29 mmol, 1 eq) in 1,2-dichloroethane (8 mL) was treated with the 4-aminobenzyl alcohol (424 mg, 3.44 mmol, 1.5 eq) and the mixture stirred at rt for 3 hr. Sodium triacetoxyborohydride (680 mg, 3.21 mmol, 1.4 eq) was then divided into two portions and added to the solution at half-hour intervals, and the solution was left to stir for 18 hr. The reaction mixture was quenched using saturated sodium bicarbonate solution (30 mL) and extracted with ethyl acetate (30 mL). The aqueous layer was further extracted with ethyl acetate (2 x 30 mL) and the combined organic layers were dried over magnesium sulfate, concentrated *in vacuo* and purified by column chromatography on aluminium oxide eluting with a gradient of ethyl acetate: hexane (20:80 → 50:50) to yield the product as a pale yellow oil (470 mg, 73%).

**R_f_** 0.44 (Al_2_O_3_; 80:20 ethyl acetate: hexane); **^1^H NMR** (400MHz, d_6_-DMSO): *δ* = 7.57-7.53 (2H, m), 7.33 (1H, dd, *J* = 8.2, 1.9 Hz), 6.99 (2H, d, *J* = 8.4 Hz), 6.50 (2H, d, *J* = 8.4 Hz), 6.27 (1H, t, *J* = 6.2 Hz), 4.80 (1H, t, *J* = 5.6 Hz), 4.29-4.23 (4H, m); **^13^C NMR** (101MHz, d_6_-DMSO): *δ* = 147.1, 142.2, 131.0, 130.5, 130.2, 129.0, 129.0, 128.1, 127.5, 112.1, 63.1, 45.4; **IR** υ*max* /cm^-1^ = 3372 (m, br, O-H), 3022 (m, C-H), 2870 (m, C-H), 1612 (s, C=C), 1519 (s, C=C), 1468 (m, C=C); **HRMS** (ESI): m/z found [M+H]+ 282.0452, C_14_H_14_NOCl_2_ requires 282.0450.

1-(cyclohex-3-en-1-yl)-N-(3,4-dichlorobenzyl)methanamine

A solution of the 3-cyclohexene-1-carboxaldehyde (375 mg, 3.40 mmol, 1 eq) in 1,2-dichloroethane (8 mL) was treated with the 3,4-dichlorobenzylamine (898 mg, 5.10 mmol, 1.5 eq) and the mixture stirred at rt for 3 hr. Sodium triacetoxyborohydride (1.01 g, 4.77 mmol, 1.4 eq) was then divided into two portions and added to the solution at half-hour intervals, and the solution was left to stir for 18 hr. The reaction mixture was quenched using saturated sodium bicarbonate solution (30 mL) and extracted with ethyl acetate (30 mL). The aqueous layer was further extracted with ethyl acetate (2 x 30 mL) and the combined organic layers were dried over magnesium sulfate, concentrated *in vacuo* and purified by column chromatography eluting with dichloromethane: methanol (98:2) to yield the product as a pale yellow oil (543 mg, 59%).

**R_f_** 0.55 (SiO_2_; 90:10 dichloromethane: methanol); **^1^H NMR** (500MHz, d_6_-DMSO): *δ* = 7.57 (1H, d, *J* = 1.9 Hz), 7.53 (1H, d, *J* = 8.2 Hz), 7.30 (1H, dd, *J* = 8.2, 1.9 Hz), 5.60 (2H, d, *J* = 2.1 Hz), 3.65 (2H, s), 2.34 (2H, d, *J* = 6.2 Hz), 2.20 (1H, s), 2.07-2.03 (1H, m), 1.97- 1.94 (2H, m), 1.77-1.72 (1H, m), 1.65-1.58 (2H, m), 1.16-1.10 (1H, m); **^13^C NMR** (126MHz, d_6_- DMSO): *δ* = 142.9, 130.8, 130.2, 129.7, 128.8, 128.2, 126.9, 126.9, 54.5, 51.9, 33.5, 29.7, 26.6, 24.4; **IR** υ*max* /cm^-1^ = 3021 (w, N-H), 2907 (m, C-H), 2835 (w, C-H), 1470 (m, C=C); **HRMS** (ESI): m/z found [M+H]+ 270.0811, C_14_H_18_NCl_2_ requires 270.0816.

(1R,2R)-4-(((3,4-dichlorobenzyl)amino)methyl)cyclohexane-1,2-diol

1-(cyclohex-3-en-1-yl)-N-(3,4-dichlorobenzyl)methanamine (300 mg, 1.11 mmol) was added to a mixture of hydrogen peroxide (30%, 2 mL) and formic acid (5 mL) at 40 °C. The solution was stirred at 40 °C for 1 hr before being cooled to rt and stirred for a further 3hr.The solvent was removed *in vacuo* and NaOH (8M, 4 mL) was added dropwise at 0 °C. The reaction was stirred at rt for 18 hr before water (20 mL) was added and the mixture extracted with ethyl acetate (30 mL). The aqueous layer was further extracted with ethyl acetate (2 x 30 mL) and the combined organic layers were dried over magnesium sulfate and concentrated *in vacuo*. The crude product was purified by flash column chromatography eluting with dichloromethane: methanol (95:5) to yield the product as a clear oil (103 mg, 31%).

**R_f_** 0.23 (SiO_2_; 85:15 dichloromethane: methanol); **^1^H NMR** (400MHz, CDCl_3_): *δ* = 7.47 (1H, d, *J* = 1.8 Hz), 7.39 (1H, d, *J* = 8.2 Hz), 7.19 (1H, dd, *J* = 8.2, 1.8 Hz), 3.77 (2H, s), 3.61-3.56 (1H, m), 3.51-3.45 (1H, m), 2.57 (2H, d, *J* = 7.4 Hz), 2.30 (2H, s), 2.00-1.79 (3H, m), 1.77-1.72 (4H, m); **^13^C NMR** (101MHz, CDCl_3_): *δ* = 139.8, 132.4, 131.1, 130.4, 130.1, 127.5, 74.1, 71.1, 52.5, 51.8, 34.1, 32.9, 27.9, 25.5; **IR** υ*max* /cm^-1^ = 3260 (s, br, O-H), 2921 (s, C-H), 2864 (m, C-H), 1472 (m, C=C); **HRMS** (ESI): m/z found [M+H]+ 304.0859, C_14_H_20_NO_2_Cl_2_ requires 304.0871.

N-(3,4-dichlorobenzyl)-2-methylpropan-1-amine

The benzaldehyde 3,4-dichlorobenzaldehyde (200 mg, 1.14 mmol, 1.0 eq) and the isobutylamine (0.17 ml, 1.7 mmol, 1.5 eq) were combined in anhydrous 1,2-dichloroethane (4 ml) under an atmosphere of nitrogen and stirred for 3 hours. Sodium triacetoxyborohydride (340 mg, 1.60 mmol, 1.4 eq) was added and the reaction was stirred at room temperature for 18 hours. The reaction mixture was poured into 2M aqueous sodium carbonate and extracted with ethyl acetate (3 × 10 mL). The combined organic extracts were dried (MgSO_4_), concentrated under reduced pressure and purified by column chromatography on silica eluting with a stepped gradient of 2N NH_3_ in methanol: ethyl acetate: hexane (1:5:94 → 1:20:79 → 1:50:49) to yield the product as a pale yellow oil (187 mg, 71%).

**R*_f_*** = 0.26 [ethyl acetate: hexane (50:50)]. **δ_H_ /ppm** (400 MHz, *d*_6_-DMSO): 7.58 (1H, d, *J*=1.9 Hz), 7.55 (1H, d, *J*=8.2 Hz), 7.31 (1H, dd, *J*=8.2 and 1.9 Hz), 3.66 (2H, s), 2.24 (2H, d, *J*=6.7 Hz), 2.16 (1H, br s), 1.70-1.60 (1H, m), 0.85 (6H, d, *J*=6.7 Hz). **δ_C_ /ppm** (101 MHz, *d*_6_-DMSO): 142.8, 130.7, 130.2, 129.6, 128.7, 128.1, 56.6, 51.7, 28.0, 20.7. **ν_max_ /cm^-1^:** 3330 (w, N-H), 2955 (s, C-H), 1564 (w, C=C), 1469 (vs, C=C). **HRMS** (ESI+) *m/z* found [M+H]+ 232.0648, C_11_H_16_NCl_2_^+^ required 232.0654 (Δ -2.8 ppm).

1-(3,4-dichlorophenyl)-N,N-dimethylmethanamine

The benzaldehyde 3,4-dichlorobenzaldehyde (600 mg, 3.43 mmol, 1.0 eq) and the dimethylamine (2.57 ml, 5.14 mmol, 2.0 M in tetrahydrofuran, 1.5 eq) were combined in anhydrous 1,2-dichloroethane (12 mL) under an atmosphere of nitrogen and stirred for 3 hours. Sodium triacetoxyborohydride (1.02 g, 4.81 mmol, 1.4 eq) was added and the reaction was stirred at room temperature for 18 hours. The reaction mixture was poured into 2M aqueous sodium carbonate and extracted with ethyl acetate (3 × 10 mL). The combined organic extracts were dried (MgSO_4_), concentrated under reduced pressure and purified by column chromatography on silica eluting with 2N NH_3_ in methanol: methanol: dichloromethane (1:5:94) to yield the product as a pale yellow oil (435 mg, 62%).

**R*_f_*** = 0.36 [2N NH_3_ in methanol: methanol: dichloromethane (1:5:94)]. **δ_H_ /ppm** (500 MHz, CDCl_3_): 7.42 (1H, d, *J*=2.0 Hz), 7.38 (1H, d, *J*=8.2 Hz), 7.14 (1H, dd, *J*=8.2 and 2.0 Hz), 3.36 (2H, s), 2.23 (6H, s). **δ_C_ /ppm** (125 MHz, CDCl_3_): 139.6, 132.4, 131.0, 130.9, 130.3, 128.3, 63.3, 45.5. **ν_max_ /cm^-1^:** 2773 (m, C-H), 1563 (w, C=C), 1470 (s, C=C). **HRMS** (ESI+) *m/z* found [M+H]^+^ 204.0335, C_9_H_12_NCl_2_^+^ required 204.0341 (Δ -2.9 ppm).

N-(3,4-dichlorobenzyl)cyclopropanamine

3,4-dichlorobenzaldehyde (300 mg, 1.71 mmol, 1.0 eq) and the cyclopropylamine (0.178 ml, 2.57 mmol, 1.5 eq) were combined in anhydrous 1,2-dichloroethane (6 ml) under an atmosphere of nitrogen and stirred for 3 hours. Sodium triacetoxyborohydride (509 mg, 2.40 mmol, 1.4 eq) was added and the reaction was stirred at room temperature for 18 hours. The reaction mixture was poured into 2M aqueous sodium carbonate and extracted with ethyl acetate (3 × 10mL). The combined organic extracts were dried (MgSO_4_), concentrated under reduced pressure and purified by column chromatography on silica eluting with a stepped gradient of 2N NH3 in methanol: ethyl acetate: hexane (1:10:89 → 1:30:69 → 1:50:49) to yield the product as a pale yellow oil (227 mg, 62%).

**R*_f_*** = 0.40 [2N NH_3_ in methanol: ethyl acetate: hexane (1:20:79)]. **δ_H_ /ppm** (400 MHz, CDCl_3_): 7.58 (1H, d, *J*=2.0 Hz), 7.37 (1H, d, *J*=8.2 Hz), 7.14 (1H, dd, *J*=8.2 and 2.0 Hz), 3.79 (2H, s), 2.16-2.09 (1H, m), 0.50-0.24 (4H, m). **δ_C_ /ppm** (101 MHz, CDCl_3_): 142.8, 132.4, 130.8, 130.4, 130.2, 127.7, 52.8, 30.2, 6.7. **ν_max_ /cm^-1^:** 3087 (w, N-H), 2930 (m, C-H), 1654 (m, C=C), 1564 (m, C=C), 1471 (vs, C=C). **HRMS** (ESI+) *m/z* found [M+H]+ 216.0338, C_10_H_12_NCl_2_^+^ required 216.0341 (Δ -1.7 ppm).

5-((3,4-dichlorobenzyl)amino)pentan-1-ol

3,4-dichlorobenzaldehyde (400 mg, 2.29 mmol, 1 eq) in 1,2-dichloroethane (8 mL) was treated with the 5- amino-1-pentanol (355 mg, 3.44 mmol, 1.5 eq) and the mixture stirred at rt for 3 hr. Sodium triacetoxyborohydride (680 mg, 3.21 mmol, 1.4 eq) was then divided into two portions and added to the solution at half-hour intervals, and the solution was left to stir for 18 hr. The reaction mixture was quenched using saturated sodium bicarbonate solution (30 mL) and extracted with ethyl acetate (30 mL). The aqueous layer was further extracted with ethyl acetate (2 x 30 mL) and the combined organic layers were dried over magnesium sulfate, concentrated *in vacuo* and purified by column chromatography eluting with dichloromethane: methanol (95:5) to yield the product as a clear oil (394 mg, 66%).

**R_f_** 0.37 (SiO_2_; 90:10 dichloromethane: methanol); **^1^H NMR** (500 MHz, CDCl_3_): *δ* = 7.44 (1H, d, *J* = 1.9), 7.39 (1H, d, *J* = 8.2 Hz), 7.17 (1H, dd, *J* = 8.2, 1.9 Hz), 3.75 (2H, s), 3.64 (2H, t, *J* = 6.5 Hz), 2.63 (2H, t, *J* = 7.0Hz), 1.80 (1H, s), 1.61-1.52 (4H, m), 1.45-1.40 (2H, m); **^13^C NMR** (126MHz, d_6_-DMSO): *δ* = 142.8, 130.8 , 130.2, 129.7, 128.8, 128.2, 60.8, 51.7, 48.7, 32.6, 29.4, 23.4; **IR** υ*max* /cm^-1^ = 3289 (m, br, O-H), 2932 (m, C-H), 2858 (m, C-H), 1564 (w, C=C), 1470 (s, C=C); **HRMS** (ESI): m/z found [M+H]+ 262.0765, C_12_H_18_NOCl_2_ requires 262.0764.

3-((3,4-dichlorobenzyl)amino)pentane-1,5-diol

A solution of dimethyl-1,3-acetonedicarboxylate (1.00 g, 5.74 mmol, 1 eq) in 1,2-dichloroethane (15 mL) was treated with 3,4-dichlorobenzylamine (1.51 g, 8.58 mmol, 1.5 eq) and the mixture stirred at rt for 3 hr. Sodium triacetoxyborohydride (1.78 g, 8.4 mmol, 1.4 eq) was then divided into two portions and added to the solution at half-hour intervals, and the solution was left to stir for 18 hr. The reaction mixture was quenched using saturated sodium bicarbonate solution (30 mL) and extracted with ethyl acetate (30 mL). The aqueous layer was further extracted with ethyl acetate (2 x 30 mL) and the combined organic layers were dried over magnesium sulfate, concentrated *in vacuo* and purified by column chromatography eluting with a gradient of dichloromethane: methanol (95:5 → 80:10) to yield the product as a pale yellow oil (297 mg, 19%).

**Rf** 0.31 (SiO2; 90:10 dichloromethane: methanol); **1H NMR** (500 MHz, d6-DMSO): *δ* = 7.56 (1H, d, *J* = 2.0 Hz, Ar CClCHCCH2), 7.53 (1H, d, *J* = 8.2 Hz, Ar CHCHCCH2), 7.29 (1H, dd, *J* = 8.2, 2.0 Hz, Ar CHCHCCH2), 3.67 (2H, s, CH2NH), 3.51-3.42 (4H, m, CH2OH), 2.68-2.62 (1H, m, NHCH), 1.57-1.47 (4H, m, CH2CH2OH); **13C NMR** (126MHz, d6-DMSO): *δ* = 143.1 (Ar CCH2), 130.7 (Ar CClCHCCH2), 130.3 (Ar CHCHCCH2), 129.8 (Ar CClCHCCH2), 128.8 (Ar CClCHCH), 128.3 (Ar CHCHCCH2), 58.8 (CH2OH), 52.5 (NHCH), 48.6 (CH2N), 36.6 (CH2CH2OH); **IR** υ*max* /cm-1 = 3288 (s, br, O-H), 2934 (m, C-H), 2866 (m, C-H), 1563 (w, N-H), 1470 (m, C=C); **HRMS** (ESI): m/z found [M+H]+ 278.0715, C12H18NO2Cl2 requires 278.0715.

2-((3,4-dichlorobenzyl)amino)butane-1,4-diol

A solution of ,4-dichlorobenzaldehyde (400 mg, 2.29 mmol, 1 eq) in 1,2-dichloroethane (8 mL) was treated with the amine α-amino-γ-butyrolactone hydrobromide (626 mg, 3.44 mmol, 1.5 eq) and the mixture stirred at rt for 3 hr. Sodium triacetoxyborohydride (680 mg, 3.21 mmol, 1.4 eq) was then divided into two portions and added to the solution at half-hour intervals, and the solution was left to stir for 18 hr. The reaction mixture was quenched using saturated sodium bicarbonate solution (30 mL) and extracted with ethyl acetate (30 mL). The aqueous layer was further extracted with ethyl acetate (2 x 30 mL) and the combined organic layers were dried over magnesium sulfate, concentrated *in vacuo* and purified by column chromatography eluting with dichloromethane: methanol (98:2) to yield the product as a white solid (203 mg, 34%).

**R_f_** 0.45 (SiO_2_; 90:10 dichloromethane: methanol); **m.p.** 87.1-89.7°C; **^1^H NMR** (500 MHz, CDCl_3_): *δ* = 7.46 (1H, d, *J* = 2.0 Hz), 7.40 (1H, d, *J* = 8.2 Hz), 7.21 (1H, dd, *J* = 8.2, 2.0 Hz), 3.85 (2H, d, *J* = 4.9 Hz), 3.82-3.75 (3H, m), 3.85 (1H, dd, *J* = 11.1, 5.2 Hz), 2.98-2.93 (1H, m), 1.83-1.70 (2H, m); **^13^C NMR** (126MHz, CDCl3): *δ* = 139.0, 132.6, 131.5, 130.6, 130.3, 127.7, 62.8, 61.1, 58.2, 49.7, 32.6; **IR** υ*max* /cm^-1^ = 3289 (m, br, O-H), 3046 (m, br, O-H), 2915 (m, C-H), 2869 (m, C-H), 1562 (w, N-H), 1467 (m, C=C); **HRMS** (ESI): m/z found [M+H]+ 264.0547, C_11_H_16_NO_2_Cl_2_ requires 264.0558.

3-((3,4-dichlorobenzyl)amino)propanamide

A mixture of 3,4-dichlorobenzylamine (440 mg, 2.5 mmol) and acrylimide (185 mg, 2.6 mmol) was treated with silicon tetrachloride (6 μl, 0.05 mmol) under solvent-free conditions at 0 °C. The mixture was heated to 60 °C and stirred for 4 hr. The reaction was allowed to cool to rt and ethyl acetate (20 mL) and water (20 mL) were added. The layers were separated and the aqueous layer was further extracted with ethyl acetate (2 x 20 mL) and the combined organic layers were dried over sodium sulfate and concentrated *in vacuo*. The crude product was purified by flash column chromatography eluting with a gradient of dichloromethane: methanol (95:5 → 85:15) to yield the product as a white solid (378 mg, 61%).

**R_f_** 0.62 (SiO_2_; 85:15 dichloromethane: methanol); **m.p.** 53.9-55.8 °C; **^1^H NMR** (400MHz, CDCl_3_): *δ* = 7.38-7.36 (2H, m), 7.12 (1H, dd, *J* = 8.2, 2.0 Hz), 6.85 (1H, s), 5.46 (2H, s), 3.74 (2H, s), 2.87 (2H, dd, *J* = 6.5, 5.4 Hz), 2.39 (2H, dd, *J* = 6.5, 5.4 Hz); **^13^C NMR** (101MHz, CDCl_3_): *δ* = 174.6, 140.0, 132.5, 131.1, 130.4, 130.0, 127.4, 52.6, 44.8, 35.4; **IR** υ*max* /cm^-1^ = 3320 (m, N-H), 3193 (m, N-H), 2837 (w, C-H), 1659 (s, C=O), 1470 (m, C=C); **HRMS** (ESI): m/z found [M+H]+ 247.0412, C_10_H_13_N_2_OCl_2_ requires 247.0405.

4-(3,4-dichlorobenzyl)morpholine

A solution of 3,4-dichlorobenzaldehyde (400 mg, 2.29 mmol, 1 eq) in 1,2-dichloroethane (8 cm3) was treated with the morpholine (300 mg, 3.44 mmol, 1.5 eq) and the mixture stirred at rt for 3 hr. Sodium triacetoxyborohydride (680 mg, 3.21 mmol, 1.4 eq) was then divided into two portions and added to the solution at half-hour intervals, and the solution was left to stir for 18 hr. The reaction mixture was quenched using saturated sodium bicarbonate solution (30 cm3) and extracted with ethyl acetate (30 cm3). The aqueous layer was further extracted with ethyl acetate (2 x 30 cm3) and the combined organic layers were dried over magnesium sulfate, concentrated *in vacuo* and purified by column chromatography eluting with a gradient of ethyl acetate: hexane (10:90 → 50:50) to yield the product as a pale yellow oil (389 mg, 69%).

**R_f_** 0.50 (SiO_2_; 1:1 ethyl acetate: hexane); **^1^H NMR** (500MHz, CDCl_3_): *δ* = 7.44 (1H, d, *J* = 1.9 Hz), 7.38 (1H, d, *J* = 8.2 Hz), 7.16 (1H, dd, *J* = 8.2, 1.9 Hz), 3.73-3.68 (4H, m), 3.43 (2H, s), 2.43-2.48 (2H, m); **^13^C NMR** (126MHz, CDCl_3_): *δ* = 138.4, 132.4, 131.0, 130.8, 130.2, 128.3, 66.9, 62.1, 53.5; **IR** υ*max* /cm^-1^ = 2854 (w, C-H), 2812 (w, C-H), 1471 (m, C=C); **HRMS** (ESI): m/z found [M+H]+ 246.0438, C_11_H_14_NOCl_2_ requires 246.0447.

N^1^-(3,4-dichlorobenzyl)propane-1,3-diamine

To a solution of tert-butyl (3-((3,4-dichlorobenzyl)amino)propyl)carbamate (260 mg, 0.780 mmol) in anhydrous dichloromethane (2.2 mL) at 0 °C, under nitrogen, was slowly added trifluoroacetic acid (0.54 ml, 7.0 mmol). The reaction was allowed to warm to room temperature and stirred for 6 hours. Dichloromethane and excess trifluoroacetic acid were removed under reduced pressure and the residue was diluted with 2M aqueous sodium carbonate and extracted with ethyl acetate (3 × 5 mL). The combined organic extracts were dried (MgSO_4_) and concentrated under reduced pressure. The crude residue was purified by column chromatography on silica eluting with a stepped gradient of 2N NH_3_ in methanol: methanol: dichloromethane (1:10:89 → 1:15:84) to yield the product as a beige solid (115 mg, 63%).

**R*_f_*** = 0.05 [methanol: dichloromethane (50:50)]. **Mp** = 98-103 °C. **δ_H_ /ppm** (500 MHz, CDCl_3_): 7.43 (1H, d, *J*=2.0 Hz), 7.38 (1H, d, *J*=8.2 Hz), 7.16 (1H, dd, *J*=8.2 and 2.0 Hz), 3.74 (2H, s), 2.79 (2H, t, *J*=6.7 Hz), 2.68 (2H, t, *J*=6.8 Hz), 1.73- 1.62 (2H, m). **δ_C_ /ppm** (125 MHz, CDCl_3_): 140.9, 132.5, 130.9, 130.4, 130.1, 127.5, 53.0, 47.5, 40.7, 33.5. **ν_max_ /cm^-1^:** 3519 (m, N-H), 2925 (s, C-H), 1624 (m, C=C), 1521 (s, C=C). **HRMS** (ESI+) *m/z* found [M+H]+ 233.0621, C_10_H_15_N_2_Cl_2_^+^ required 233.0612 (Δ 3.9 ppm).

N^1^-(3,4-dichlorobenzyl)ethane-1,2-diamine

To a solution of *tert*-butyl (2-((3,4-dichlorobenzyl)amino)ethyl)carbamate (286 mg, 0.896 mmol) in anhydrous dichloromethane (2.5 ml) at 0 °C, under nitrogen, was slowly added trifluoroacetic acid (0.62 ml, 8.1 mmol). The reaction was allowed to warm to room temperature and stirred for 6 hours. Dichloromethane and excess trifluoroacetic acid were removed under reduced pressure and the residue was diluted with 2M aqueous sodium carbonate and extracted with ethyl acetate (3 × 5 mL). The combined organic extracts were dried (MgSO_4_) and concentrated under reduced pressure. The crude residue was purified by column chromatography on silica eluting with a stepped gradient of 2N NH_3_ in methanol: methanol: dichloromethane (1:10:89 → 1:15:84) to yield the product as a pale orange solid (197 mg, 100%).

**R*_f_*** = 0.20 [2N NH_3_ in methanol: methanol: dichloromethane (1:15:84)]. **Mp** = 78-79 °C. **δ_H_ /ppm** (400 MHz, *d*_6_-DMSO): 7.65 (1H, d, *J*=1.5 Hz), 7.57 (1H, d, *J*=8.2 Hz), 7.34 (1H, dd, *J*=8.2 and 1.5 Hz), 3.69 (2H, s), 2.82 (2H, t, *J*=6.0 Hz), 2.62 (2H, t, *J*=6.0 Hz), **δ_C_ /ppm** (101 MHz, CDCl_3_): 140.1, 132.6, 131.2, 130.6, 130.1, 127.6, 52.4, 49.4, 40.6. **ν_max_ /cm^-1^:** 3303 (w, N-H), 2933 (m, C-H), 1667 (s, C=C), 1544 (m, C=C). **HRMS** (ESI+) *m/z* found [M+H]+ 219.0454, C_9_H_13_N_2_C_l2_^+^ required 219.0456 (Δ -0.9 ppm).

(1-(3,4-dichlorobenzyl)piperidin-3-yl)methanol

A solution of 3,4-dichlorobenzaldehyde (400 mg, 2.29 mmol, 1 eq) in 1,2-dichloroethane (8 mL) was treated with piperidin-3-ylmethanol (396 mg, 3.44 mmol,1.5 eq) and the mixture stirred at rt for 3 hr. Sodium triacetoxyborohydride (680 mg, 3.21 mmol, 1.4 eq) was then divided into two portions and added to the solution at half-hour intervals, and the solution was left to stir for 18 hr. The reaction mixture was quenched using saturated sodium bicarbonate solution (30 mL) and extracted with ethyl acetate (30 mL). The aqueous layer was further extracted with ethyl acetate (2 x 30 mL) and the combined organic layers were dried over magnesium sulfate, concentrated *in vacuo* and purified by column chromatography eluting with dichloromethane: methanol (95:5) to yield the product as an orange oil (540 mg, 86%).

**R_f_** 0.56 (SiO_2_; 85:15 dichloromethane: methanol); **^1^H NMR** (500MHz, d_6_-DMSO): *δ* = 7.55 (1H, d, *J* = 8.2 Hz), 7.50 (1H, d, *J* = 1.9 Hz), 7.27 (1H, dd, *J* = 8.2, 1.9 Hz), 4.37 (1H, t, *J* = 5.3 Hz), 3.40 (2H, d, *J* = 10.2 Hz), 3.28-3.23 (1H, m), 3.17-3.12 (1H, m), 2.77 (1H, d, *J* = 9.0 Hz), 2.65 (1H, d, *J* = 11.0 Hz), 1.88 (1H, dt, *J* = 2.3, 11.0 Hz), 1.65-1.55 (4H, m), 1.46-1.37 (1H, m), 0.89-0.81 (1H, m); **^13^C NMR** (126MHz, d_6_-DMSO): *δ* = 140.3, 130.8, 130.4, 130.4, 129.3, 129.0, 64.3, 61.2, 57.0, 53.9, 38.9, 27.0, 24.7; **IR** υ*_max_* /cm^-1^ = 3288 (m, br, O-H), 2930 (m, C-H), 2853 (w, C-H), 2808 (w, C-H), 1469 (s, C=C); **HRMS** (ESI): m/z found [M+H]+ 274.0765, C_13_H_18_NOCl_2_ requires 274.0758.

(1S,2S)-2-((3,4-dichlorobenzyl)amino)cyclopentan-1-ol

A solution of 3,4-dichlorobenzaldehyde (400 mg, 2.29 mmol, 1 eq) in methanol (8 mL) was treated with aminocyclopentanol hydrochloride (473 mg, 3.44 mmol, 1.5 eq) and triethylamine (0.5 cm3, 3.58 mmol, 1.5 eq), and the mixture stirred at rt for 3 hr. Sodium triacetoxyborohydride (680 mg, 3.21 mmol, 1.4 eq) was then divided into two portions and added to the solution at half-hour intervals, and the solution was left to stir for 18 hr. The reaction mixture was quenched using NaOH (2M, 30 mL) and extracted with dichloromethane (30 mL). The aqueous layer was further extracted with dichloromethane (2 x 30 mL) and the combined organic layers were dried over magnesium sulfate, concentrated *in vacuo* and purified by column chromatography eluting with dichloromethane: methanol (95:5) to yield the product as a pale pink solid (368 mg, 65%).

**R_f_** 0.60 (SiO_2_; 85:15 dichloromethane: methanol); **m.p.** 84.6-85.3°C; **^1^H NMR** (500MHz, d_6_-DMSO): *δ* = 7.57 (1H, d, *J* = 1.9 Hz), 7.53 (1H, d, *J* = 8.2 Hz), 7.30 (1H, dd, *J* = 8.2, 1.9 Hz,), 4.47 (1H, d, *J* = 4.2 Hz), 3.75-3.71 (1H, m), 3.67 (1H, d, *J* = 3.0 Hz), 2.69-2.66 (1H, m,), 2.15 (1H, s), 1.81-1.74 (2H, m), 1.57-1.50 (2H, m), 1.40-1.33 (1H, m), 1.27-1.20 (1H, m); **^13^C NMR** (126MHz, d_6_-DMSO): *δ* = 143.0, 130.7, 130.2, 129.8, 128.8, 128.3, 76.7, 65.46, 50.1, 32.8, 30.0, 20.8; **IR** υ*_max_* /cm^-1^ = 3259 (m, N-H), 3050 (m, br, O-H), 2955 (m, C-H), 2862 (m, C-H), 1468 (s, C=C); **HRMS** (ESI): m/z found [M+H]+ 260.0603 C_12_H_16_NOCl_2_ requires 260.0609.

(2-chloro-2'-ethyl-[1,1'-biphenyl]-4-yl)methanamine

To a stirred suspension of LiAlH_4_ (42 mg, 1.1 mmol, 2.0 eq) in Et_2_O (5.0 mL) was added AlCl_3_ (147 mg, 1.1 mmol, 2.0 eq) and the reaction mixture cooled to 0 °C for 10 minutes. The reaction was allowed to warm to room temperature and the 2-chloro-2'-ethyl-[1,1'-biphenyl]-4-carbonitrile (132 mg, 0.55 mmol, 1.0 eq) was added portionwise. The reaction was stirred at room temperature for 30 minutes and then heated at 50 °C overnight. After cooling to room temperature, a saturated aqueous solution of potassium sodium tartrate tetrahydrate and Et_2_O were added and the mixture stirred for 1 hour. The reaction mixture was diluted with 2M aqueous NaCO_3_ and extracted three times with Et_2_O. The combined organic extracts were washed with a saturated aqueous solution of NaCl, dried over MgSO_4_, filtered and concentrated *in vacuo*. The crude product was then purified by flash column chromatography (silica gel, 1:5:14 NH_3_:MeOH:CH_2_Cl_2_) to provide free amine. The free amine was then dissolved in CH_2_Cl_2_ (0.5 mL) and HCl (2M in Et_2_O) (2.75 mL, 5.5 mmol) added dropwise. The reaction was stirred for 1 hour before the precipitate was filtered, washed with cold Et_2_O and dried to provide the title compound as a white solid (65 mg, 0.23 mmol, 43%):

**R*_f_*** 0.18 (1:10 MeOH:CH_2_Cl_2_); **m.p.** 181-183 °C; **IR** υ_max_ 3300 – 2300, 2963, 2871, 2612, 1601,1504, 1476, 1446, 1401, 1378, 1214, 1076, 1006; **^1^H NMR** (500 MHz, DMSO-d_6_) δ 8.63 (s, 3H), 7.78 (d, *J* = 1.6 Hz, 1H), 7.55 (dd, *J* = 7.8, 1.7 Hz, 1H), 7.39 – 7.33 (m, 3H), 7.28 – 7.23 (m, 1H), 7.04 (d, *J* = 7.3 Hz, 1H), 4.09 (s, 2H), 2.46 – 2.24 (m, 2H), 0.98 (t, *J* = 7.6 Hz, 3H); **^13^C NMR** (126 MHz, DMSO-d_6_) δ 141.4, 139.7, 137.7, 135.4, 132.3, 131.5, 129.7, 129.3, 128.3 (br s, 2C), 127.8, 125.7, 41.3, 25.7, 15.1; **HRMS** (ESI) calcd for [C_15_H_16_N^35^Cl + H]^+^: 246.1049, found: 458.0961.

(2-chloro-[1,1'-biphenyl]-4-yl)methanamine

To a suspension of lithium aluminium hydride (11 mg, 0.28 mmol, 2.0 eq) in anhydrous diethyl ether (1 ml), under nitrogen, was added aluminium chloride (37 mg, 0.28 mmol, 2.0 eq). The reaction mixture was cooled to 0 °C and stirred for 10 minutes. The reaction was allowed to warm to room temperature and the 2-chloro-[1,1'-biphenyl]-4-carbonitrile (30 mg, 0.14 mmol, 1.0 eq) was added portionwise. The reaction was stirred at room temperature for 30 minutes and then heated to 40 °C for 18 hours. The resultant suspension was allowed to cool to room temperature and ethyl acetate and saturated aqueous potassium sodium tartrate tetrahydrate were added and the mixture stirred for 1 hour. The reaction mixture was poured into 2M aqueous sodium carbonate and extracted with ethyl acetate (3×). The combined organic extracts were dried (MgSO4) and concentrated under reduced pressure to yield the desired product. The crude amine (24 mg, 0.11 mmol) was converted to the hydrochloride salt using hydrochloric acid (0.60 ml, 1.2 mmol, 2 M in diethyl ether) according to general method 9 to yield the product as a white crystalline solid (17.0 mg, 48%).

**Mp** = 270-273 °C. **δ_H_ /ppm** (500 MHz, *d*_6_-DMSO): 8.45 (3H, br s), 7.75 (1H, d, *J*=1.7 Hz), 7.54 (1H, dd, *J*=7.9 and 1.7 Hz), 7.51-7.40 (6H, m), 4.09 (2H, s). **δ_C_ /ppm** (125 MHz, *d*_6_-DMSO): 139.8, 138.2, 135.3, 131.6, 131.2, 130.3, 129.1, 128.3, 128.1, 128.0, 41.3. **ν_max_ /cm^-1^:** 3420 (w, N-H), 2913 (vs, C-H), 1607 (m, C=C), 1514 (m, C=C). **HRMS** (ESI+) *m/z* found [M-Cl]+ 218.0729, C_13_H_13_NCl+ required 218.0737 (Δ -3.7 ppm).

(3-chloro-4-(naphthalen-1-yl)phenyl)methanaminium

To a stirred suspension of LiAlH_4_ (9 mg, 0.24 mmol, 2.0 eq) in Et_2_O (0.5 mL) was added AlCl_3_ (32 mg, 0.24 mmol, 2.0 eq) and the reaction mixture cooled to 0 °C for 10 minutes. The reaction was allowed to warm to room temperature and 3-Chloro-4-(naphthalen-1-yl)benzonitrile (31 mg, 0.12 mmol, 1.0 eq) was added portionwise. The reaction was stirred at room temperature for 30 minutes and then heated at 50 °C overnight. After cooling to room temperature, a saturated aqueous solution of potassium sodium tartrate tetrahydrate and Et_2_O were added and the mixture stirred for 1 hour. The reaction mixture was diluted with 2M aqueous Na_2_CO_3_ and extracted three times with Et_2_O. The combined organic extracts were washed with a saturated aqueous solution of NaCl, dried over MgSO_4_, filtered and concentrated *in vacuo*. The crude amine was dissolved in CH_2_Cl_2_ (0.5 mL) and HCl (2M in Et_2_O) (0.6 mL, 1.2 mmol) added dropwise. The reaction was stirred for 1 hour before the precipitate was filtered, washed with cold Et_2_O and dried to provide the title compound as a yellow solid (14 mg, 0.05 mmol, 38%):

**HPLC** *t_r_* = 9.40 mins (5-95% B); **m.p.** 246.3–246.7 °C; **IR** υ_max_ 3377, 2901, 1592, 1493, 1395, 1377, 1248, 1215, 1185, 1123, 1065, 1051, 1017, 964, 887, 833, 802, 774, 688, 650; **^1^H NMR** (400 MHz, DMSO-d_6_) δ 8.56 (s, 3H), 8.06 – 7.99 (m, 2H), 7.84 (d, *J* = 1.5 Hz, 1H), 7.65 – 7.59 (m, 2H), 7.58 – 7.53 (m, 1H), 7.52 – 7.44 (m, 2H), 7.38 (dd, *J* = 7.0, 1.0 Hz, 1H), 7.30 (d, *J* = 8.3 Hz, 1H), 4.16 (d, *J* = 3.9 Hz, 2H); **^13^C NMR** (101 MHz, DMSO-d_6_) δ 138.6, 136.2, 135.9, 133.1, 132.9, 132.3, 130.9, 129.9, 128.4 (br s, 2C), 128.0, 127.1, 126.6, 126.1, 125.5, 124.9, 41.4; **HRMS** (ESI) calcd for [C_17_H_14_N^35^Cl]: 267.0815, found: 267.0811.

(4'-(aminomethyl)-2'-chloro-[1,1'-biphenyl]-2-yl)methanol

To a suspension of LiAlH_4_ (15.0 mg, 400 μmol 2.0 equiv) in Et_2_O (3.70 mL) at 0°C was added AlCl_3_ (27.0 mg, 200 μmol, 1.0 equiv) and the mixture stirred for 10 minutes. 2-Chloro-[2'-(hydroxymethyl)- 1,1'-biphenyl]-4-carbonitrile (50.0 mg, 200 μmol 1.0 equiv) was added and the reaction mixture stirred at room temperature for 30 minutes and then refluxed overnight. The mixture was cooled to 0 °C, diluted with EtOAc and a saturated aqueous solution of potassium sodium tartrate tetrahydrate was added. The suspension was stirred for 1 hour and then poured into a 2 M aqueous solution of Na_2_CO_3_. The aqueous phase was extracted with EtOAc (3x). The combined organic phases were washed with brine and dried (MgSO_4_). The product was purified by column chromatography on silica gel (CH2Cl2:MeOH:NH3, 8:2:0.05)) to afford compound as a colourless oil (27.0 mg, 110 μmol, 54%).

**R*_f_*** = 0.23 [CH_2_Cl_2_:NH_3_ (8:2:0.05)]; **δ_H_** (500 MHz, CD_3_OD): 7.59 (1H, dd, *J =* 7.0 and 1.5 Hz), 7.51 (1H, d, *J =* 1.5 Hz), 7.42 (1H, td, *J =* 7.7 and 1.2 Hz), 7.35-7.29 (2H, m, H5), 7.24 (1H, d, *J =* 7.7 Hz), 7.09 (1H, dd, *J =* 7.7 and 1.2 Hz), 4.42 (1H, d, *J =* 13.6 Hz), 4.30 (1H, d, *J* = 13.6 Hz), 3.8 (2H, s); **δ_C_** (125 MHz, CD_3_OD): 143.1, 139.1, 138.0, 137.6, 133.0, 131.1, 129.26, 128.1, 127.7, 126.7, 126.5, 125.5, 61.5, 44.7; **ν_max_:** 3380 (N-H, br), 2977 (O-H, br); **HRMS** (ESI+) m/z found [M+H]+ 248.0834, C_14_H_15_NOCl+ required 248.0837 (Δ -1.0 ppm).

(2'-((1H-pyrrol-1-yl)methyl)-2-chloro-[1,1'-biphenyl]-4-yl)methanamine

To a solution of the appropriate 2'-((1*H*-pyrrol-1-yl)methyl)-2-chloro-[1,1'-biphenyl]-4-carbonitrile (30.0 mg, 100 μmol, 1 eq) in NH_3_ (8% in MeOH, 0.15 M) was added a spatula of Raney Nickel (slurry solution). A H_2_ atmosphere was applied and the reaction mixture was vigorously stirred at room temperature overnight. Once the reaction was completed, as monitored by TLC, EtOAc was added and the suspension filtered under gravity. The filtrate was concentrated under vacuum and the product purified by column chromatography on silica gel (CH_2_Cl_2_:MeOH:NH_3_ from 95:5:0 to 95:4.5:0.5). Compound was obtained as a colourless viscous oil (8.50 mg, 29.0 μmol, 28%).

**R*_f_*** = 0.51 [CH_2_Cl_2_:MeOH:NH_3_ (80:18:2)]; **δ_H_** (400 MHz, CD_3_OD): 7.56 (1H, s,), 7.36-7.31 (3H, m), 7.16 (1H, d, *J =* 7.5 Hz), 7.14-7.11 (1H, m), 7.03-7.00 (1H, m), 6.48 (2H, t, *J =* 2.0 Hz), 6.01 (2H, t, *J =* 2.0 Hz), 4.90 (1H, d, *J =* 17 Hz), 4.80 (1H, d, *J =* 17 Hz), 3.86 (2H, s); **δ_C_** (101 MHz, CD_3_OD): 144.1, 138.1, 137.5, 136.7, 132.9, 131.1, 129.5, 128.0, 127.9, 127.4, 127.1, 125.8, 120.6, 107.6, 50.4, 44.5; **ν_max_:** 2989 (N-H, br), 1264 (C-N, s), 732 and 703 (=C-H, s); **HRMS** (ESI+) m/z found [M+H]+ 297.1194, C_18_H_18_N_2_Cl+ required 297.1153 (Δ -1.2 ppm).

2-(4-(aminomethyl)-2-chlorophenoxy)ethan-1-amine

To a suspension of lithium aluminium hydride (290 mg, 7.64 mmol, 2.0 eq) in anhydrous diethyl ether (10 ml), under nitrogen, was added aluminium chloride (679 mg, 5.09 mmol, 2.0 equiv). The reaction mixture was cooled to 0 °C and stirred for 10 minutes. The reaction was allowed to warm to room temperature and 3-Chloro-4-(cyanomethoxy)benzonitrile (490 mg, 2.55 mmol, 1.0 eq) was added portionwise. The reaction was stirred at room temperature for 30 minutes and then heated to 40 °C for 18 hours. The resultant suspension was allowed to cool to room temperature and ethyl acetate and saturated aqueous potassium sodium tartrate tetrahydrate were added and the mixture stirred for 1 hour. The reaction mixture was poured into 2M aqueous sodium carbonate and extracted with ethyl acetate (3×). The combined organic extracts were dried (MgSO_4_) and concentrated under reduced pressure. The crude amine (336 mg, 1.68 mmol, 1.0 eq) was converted to the hydrochloride salt using hydrochloric acid (8.4 ml, 17 mmol, 2 M in diethyl ether) stirring for 1 hour then filtering and washing with cold diethyl ether. The precipitate was recrystallised from ethanol to yield the product as a cream crystalline solid (264 mg, 38%).

**Mp** > 300 °C. **δ_H_ /ppm** (500 MHz, *d*_6_-DMSO): 8.48 (3H, br s), 8.34 (3H, br s), 7.66 (1H, d, *J*=2.2 Hz), 7.46 (1H, dd, *J*=8.5 and 2.2 Hz), 7.25 (1H, d, *J*=8.5 Hz), 4.30 (2H, t, *J*=5.4 Hz), 3.96 (2H, s), 3.27-3.18 (2H, m). **δ_C_ /ppm** (125 MHz, *d*_6_-DMSO): 153.3, 130.9, 129.4, 128.2, 121.4, 114.3, 65.7, 41.0, 38.0. **ν_max_ /cm^-1^:** 3237 (m, N-H), 2960 (s, C-H), 1608 (m, C=C), 1505 (m, C=C), 1461 (s, C=C). **HRMS** (ESI+) *m/z* found [M-H-2Cl]+ 201.0782, C_9_H_14_N_2_OCl+ required 201.0789 (Δ -3.4 ppm).

(3-chloro-4-propoxyphenyl)methanamine

To a suspension of lithium aluminium hydride (117 mg, 3.07 mmol, 2.0 eq) in anhydrous diethyl ether (6 ml), under nitrogen, was added aluminium chloride (408 mg, 3.07 mmol, 2.0 eq). The reaction mixture was cooled to 0 °C and stirred for 10 minutes. The reaction was allowed to warm to room temperature and 3-chloro-4-propoxybenzonitrile (300 mg, 1.53 mmol, 1.0 equiv) was added portionwise. The reaction was stirred at room temperature for 30 minutes and then heated to 40 °C for 18 hours. The resultant suspension was allowed to cool to room temperature and ethyl acetate and saturated aqueous potassium sodium tartrate tetrahydrate were added and the mixture stirred for 1 hour. The reaction mixture was poured into 2M aqueous sodium carbonate and extracted with ethyl acetate (3×). The combined organic extracts were dried (MgSO4) and concentrated under reduced pressure The crude amine (285 mg, 1.42 mmol) was converted to the hydrochloride salt using hydrochloric acid (7.1 ml, 14 mmol, 2 M in diethyl ether) stirring for 1 hour then filtering and washing with cold diethyl ether to yield the product as an off-white crystalline solid (222 mg, 61%).

**Mp** = 246-249 °C. **δ_H_ /ppm** (500 MHz, *d*_6_-DMSO): 8.37 (3H, br s), 7.60 (1H, d, *J*=2.2 Hz), 7.41 (1H, dd, *J*=8.5 and 2.2 Hz), 7.17 (1H, d, *J*=8.5 Hz), 4.03 (2H, t, *J*=6.4 Hz), 3.95 (2H, s,), 1.81-1.68 (2H, m), 0.99 (3H, t, *J*=7.4 Hz). **δ_C_ /ppm** (125 MHz, *d*_6_-DMSO): 154.4, 131.2, 129.8, 127.5, 121.6, 114.1, 70.5, 41.6, 22.3, 10.8. **ν_max_ /cm^-1^:** 2963 (vs, C-H), 1610 (m, C=C), 1506 (s, C=C). **HRMS** (ESI+) *m/z* found [M-Cl]+ 200.0832, C_10_H_15_NOCl+ required 200.0837 (Δ -2.2 ppm).

(3-chloro-4-(cyclopentyloxy)phenyl)methanamine

To a suspension of lithium aluminium hydride (103 mg, 2.71 mmol, 2.0 equiv) in anhydrous diethyl ether (5 ml), under nitrogen, was added aluminium chloride (361 mg, 2.71 mmol, 2.0 equiv). The reaction mixture was cooled to 0 °C and stirred for 10 minutes. The reaction was allowed to warm to room temperature and 3-Chloro-4-(cyclopentyloxy)benzonitrile (300 mg, 1.35 mmol, 1.0 equiv) was added portionwise. The reaction was stirred at room temperature for 30 minutes and then heated to 40 °C for 18 hours. The resultant suspension was allowed to cool to room temperature and ethyl acetate and saturated aqueous potassium sodium tartrate tetrahydrate were added and the mixture stirred for 1 hour. The reaction mixture was poured into 2M aqueous sodium carbonate and extracted with ethyl acetate (3×). The combined organic extracts were dried (MgSO_4_) and concentrated under reduced pressure. The crude amine (230 mg, 1.02 mmol) was converted to the hydrochloride salt using hydrochloric acid (5.1 ml, 10 mmol, 2 M in diethyl ether) stirring for 1 hour then filtering and washing with cold diethyl ether. The precipitate was recrystallised from ethanol to yield the product as an off-white crystalline solid (38.8 mg, 11%).

**Mp** = 240-243 °C. **δ_H_ /ppm** (500 MHz, *d*_6_-DMSO): 8.32 (3H, br s), 7.58 (1H, d, *J*=2.2 Hz), 7.39 (1H, dd, *J*=8.6 and 2.2 Hz), 7.18 (1H, d, *J*=8.6 Hz), 4.96-4.90 (1H, m), 3.94 (2H, s), 1.99-1.82 (2H, m), 1.80-1.66 (4H, m), 1.66- 1.52 (2H, m). **δ_C_ /ppm** (125 MHz, *d*_6_-DMSO): 153.1, 130.8, 129.2, 126.9, 121.9, 115.1, 80.2, 41.2, 32.2, 23.5. **ν_max_ /cm^-1^:** 2913 (s, C-H), 1601 (m, C=C), 1504 (s, C=C). **HRMS** (ESI+) *m/z* found [M-Cl]+ 226.0984, C_12_H_17_NOCl+ required 226.0993 (Δ -4.2 ppm).

SI_3 List of fragments screened

| Compound | clogP | IC_50_ (mM) |
| --- | --- | --- |
|  | 3.04 | 0.90 |
|  | 2.41 | 0.98 |
|  | 2.26 | 3.00 |
|  | 2.84 | 5.00 |
|  | 2.82 | 5.00 |
|  | 3.39 | 5.00 |
|  | 2.25 | 5.00 |
|  | 2.81 | 5.00 |
|  | 2.43 | 5.00 |
|  | 3.64 | 5.00 |
|  | 2.89 | 5.00 |
|  | 4.09 | 5.00 |
|  | 2.01 | 0.27 |
|  | 2.74 | 0.75 |
|  | 3.15 | 0.83 |
|  | 3.15 | 0.90 |
|  | 1.70 | 1.01 |
|  | 2.68 | 1.02 |
|  | 2.82 | 1.48 |
|  | 3.62 | 1.80 |
|  | 2.71 | 1.90 |
|  | 2.11 | 2.30 |
|  | 2.63 | 2.39 |
|  | 3.61 | 2.80 |
|  | 4.49 | 3.00 |
|  | 2.39 | 3.00 |
|  | 3.98 | 5.00 |
|  | 3.12 | 5.00 |
|  | 3.21 | 5.00 |
|  | 3.07 | 5.00 |
|  | 1.54 | 5.00 |
|  | 1.48 | 5.00 |
|  | 1.65 | 5.00 |
|  | 2.90 | 5.00 |
|  | 2.00 | 5.00 |
|  | 1.94 | 5.00 |
|  | 2.94 | 5.00 |
|  | 3.02 | 5.00 |
|  | 3.49 | 0.150 |
|  | 4.31 | 0.160 |
|  | 4.60 | 0.205 |
|  | 3.86 | 0.208 |
|  | 3.19 | 0.493 |
|  | -0.58 | 0.500 |
|  | 4.34 | 0.500 |
|  | 3.13 | 0.500 |
|  | 2.58 | 0.500 |
|  | 2.48 | 0.500 |
|  | 4.38 | 0.500 |
|  | 2.35 | 3.00 |
|  | 2.32 | 3.00 |
|  | 0.75 | 3.00 |
|  | -1.7 | 3.00 |
|  | -1.33 | 5.00 |
|  | 4.39 | 0.300 |
|  | 4.39 | 0.500 |
|  | 4.17 | 0.500 |
|  | 3.33 | 0.500 |
|  | 2.09 | 3.00 |
|  | 2.78 | 3.00 |
|  | 9.11 | 3.00 |
|  | -1.29 | 3.00 |
|  | 3.45 | 0.044 |

SI_4 Selected NMR Spectra

(3-chloro-5-(1H-indol-4-yl)phenyl)methanamine, **7b**


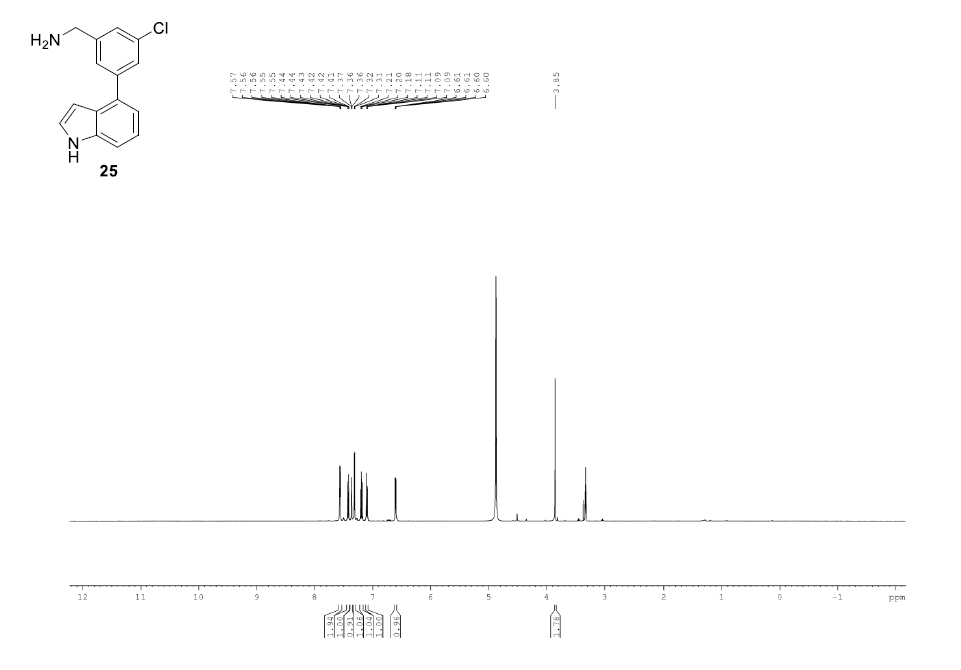


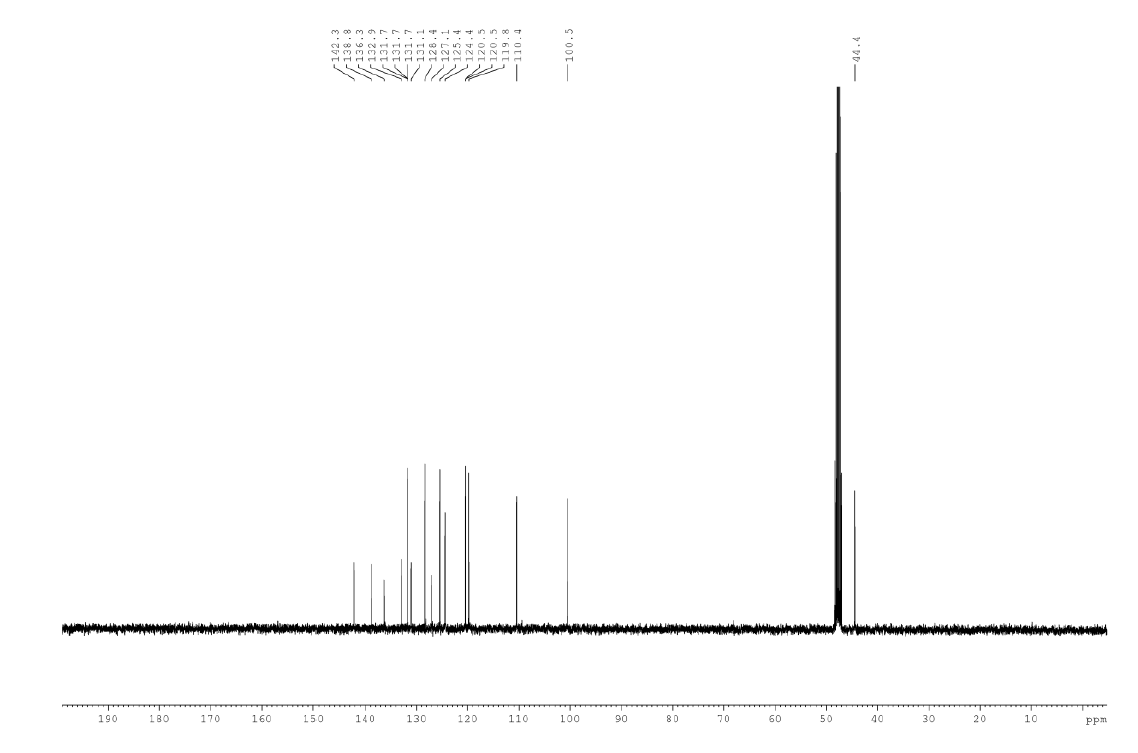


2-Chloro-2'-fluoro-[1,1'-biphenyl]-4-carbonitrile, 2b


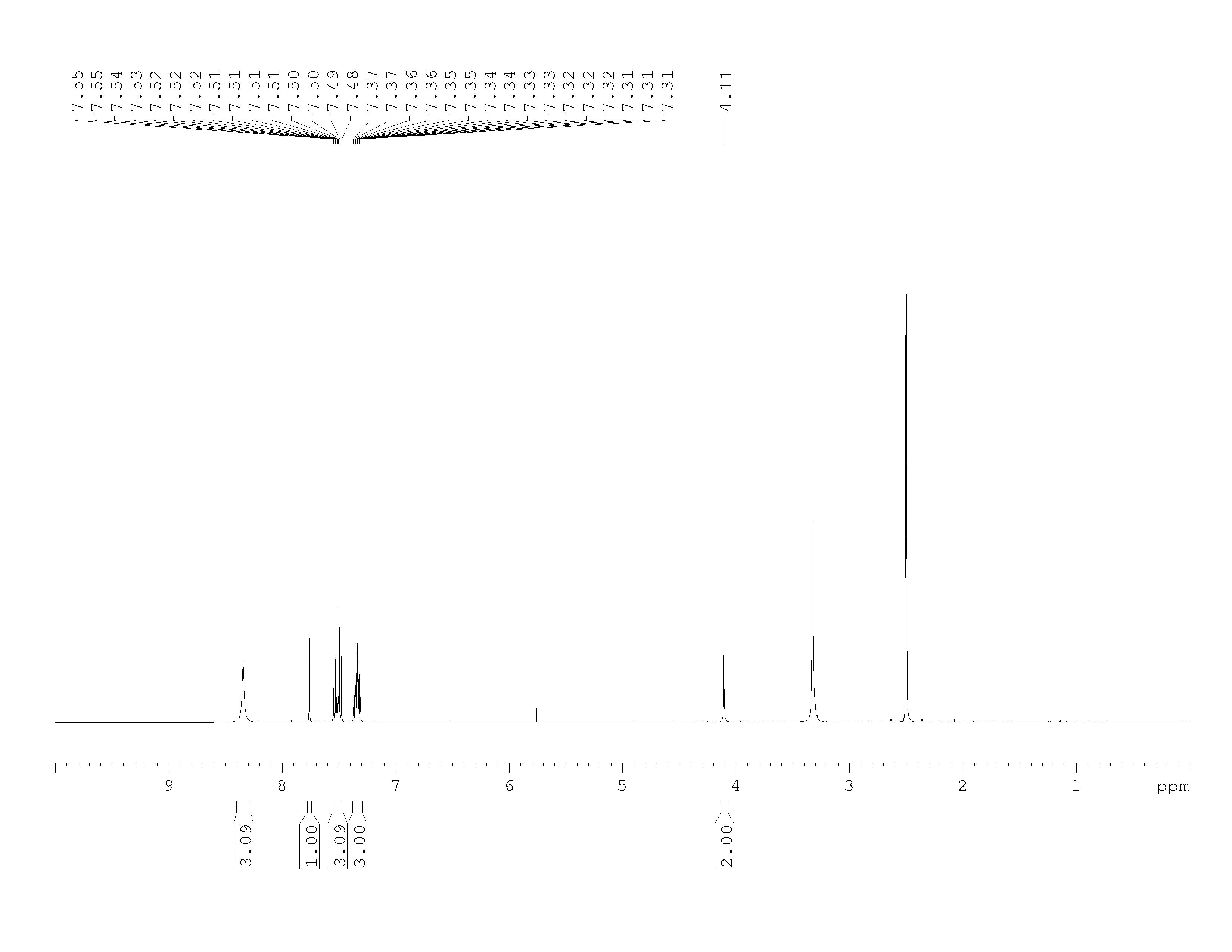

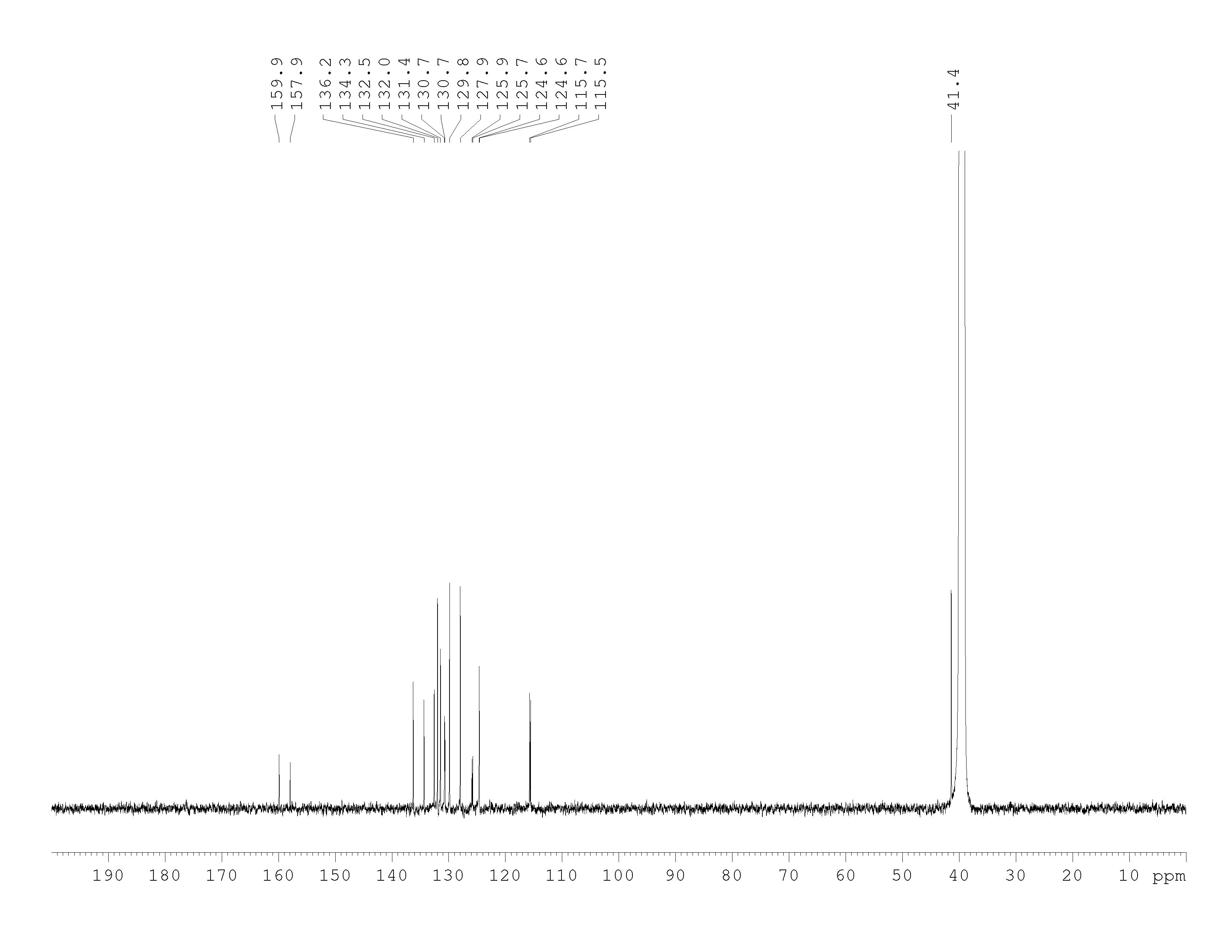


(2-chloro-2’-isopropyl-[1,1’-biphenyl]-4-yl)methanaminium chloride, 3b


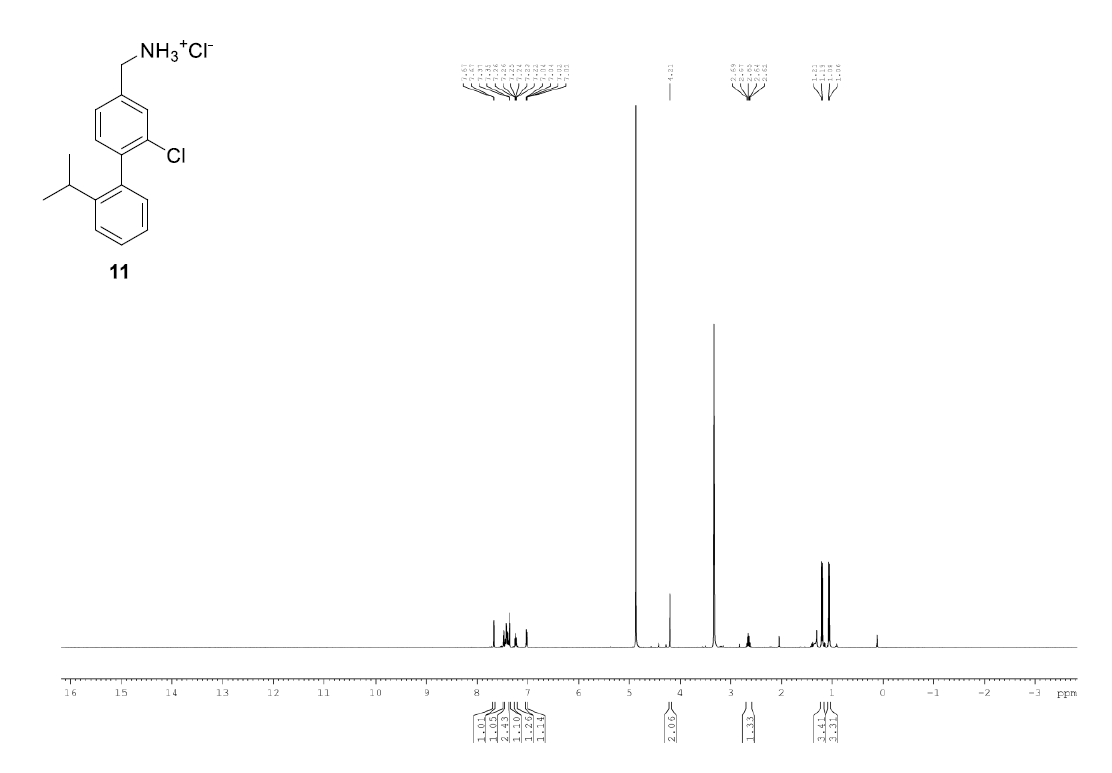

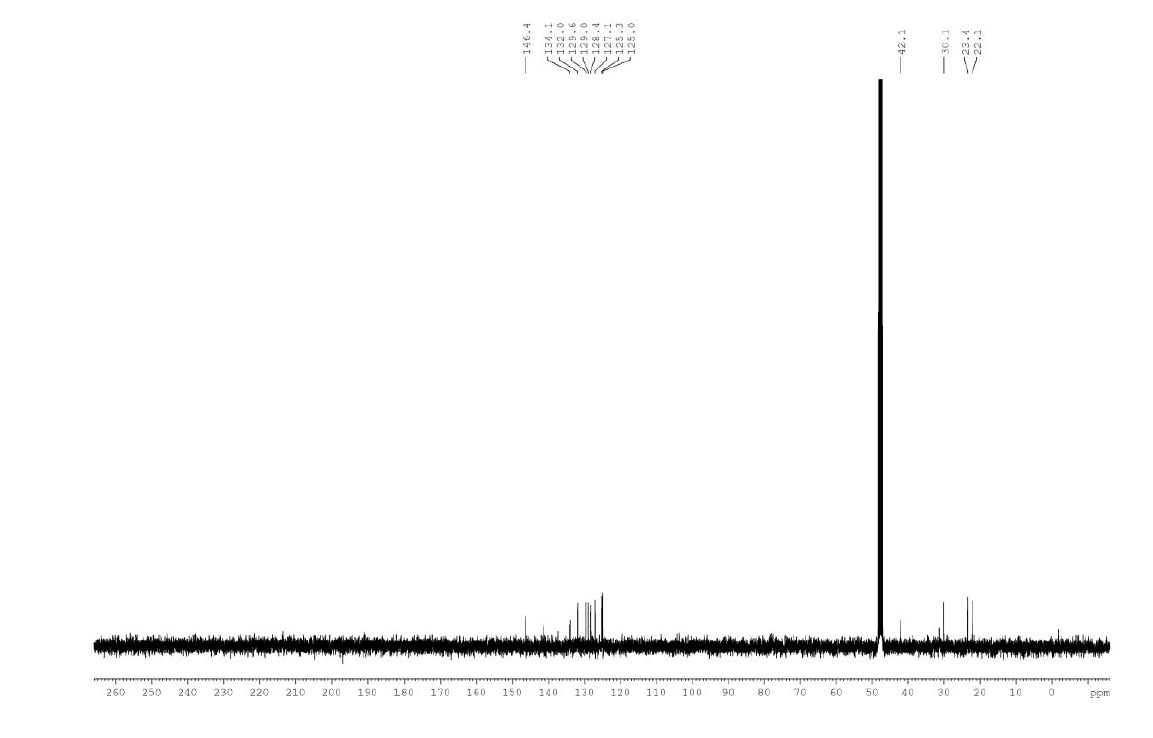


(2-Chloro-2'-methyl-[1,1'-biphenyl]-4-yl)methanamine HCl salt, 4b


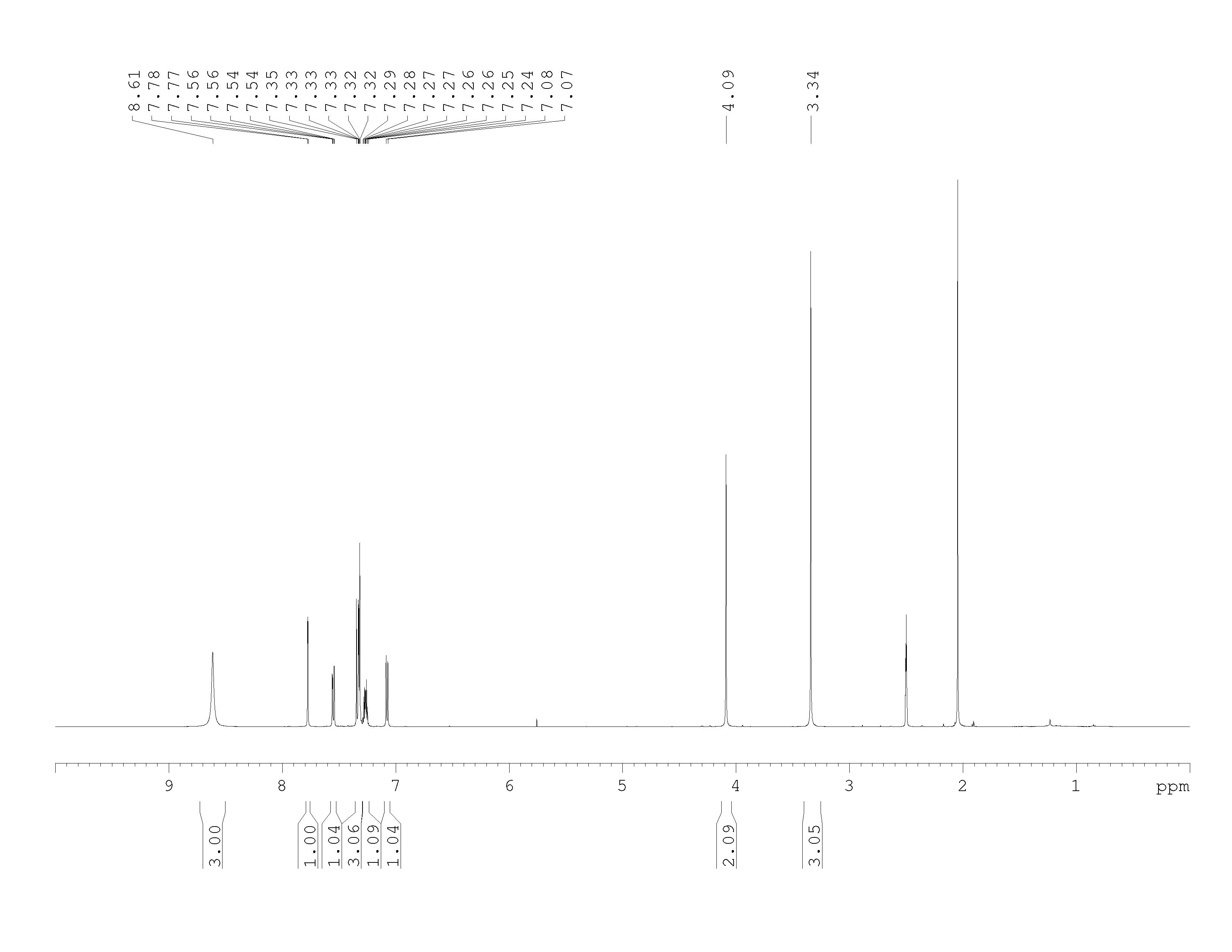

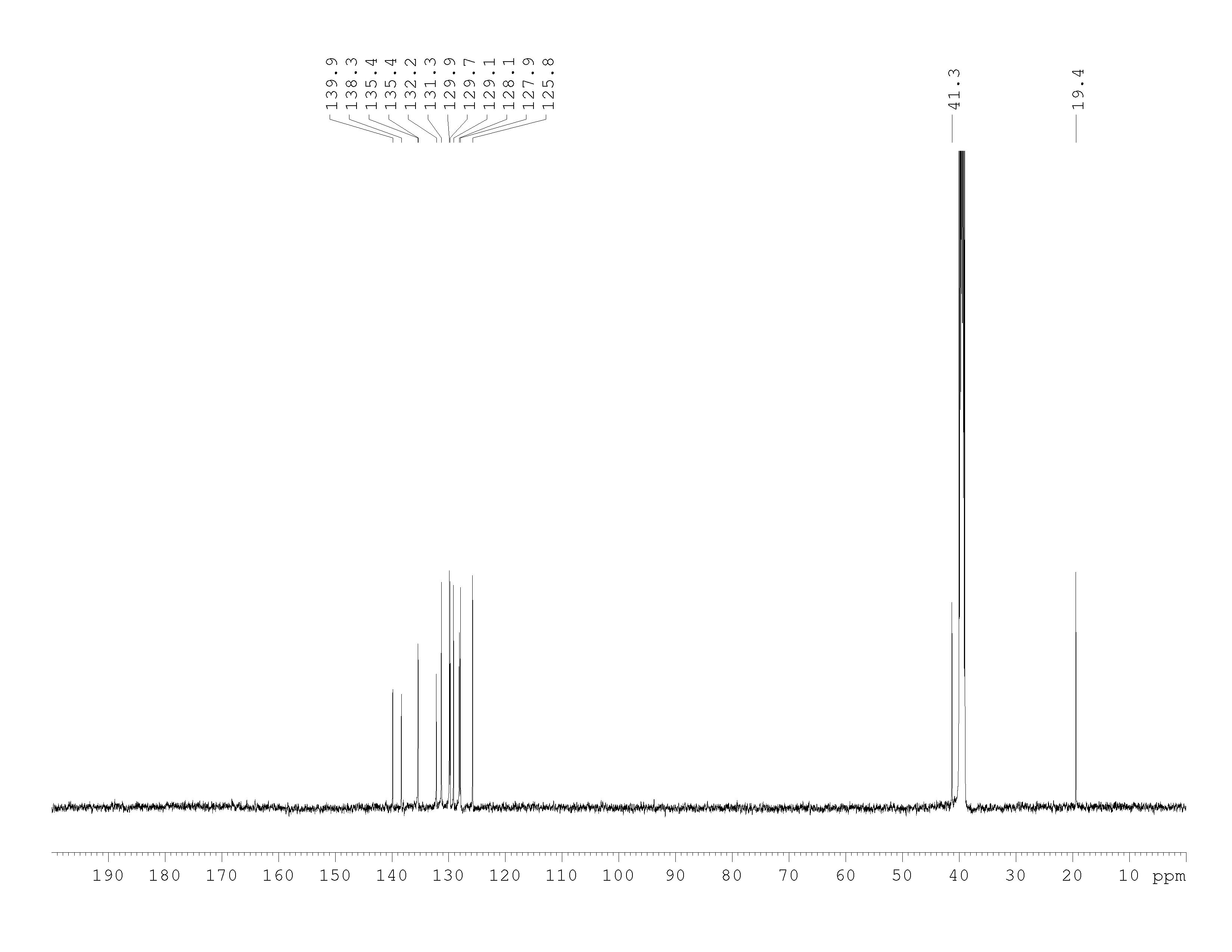


(2-Chloro-2'-methoxy-[1,1'-biphenyl]-4-yl)methanamine HCl salt, 5b


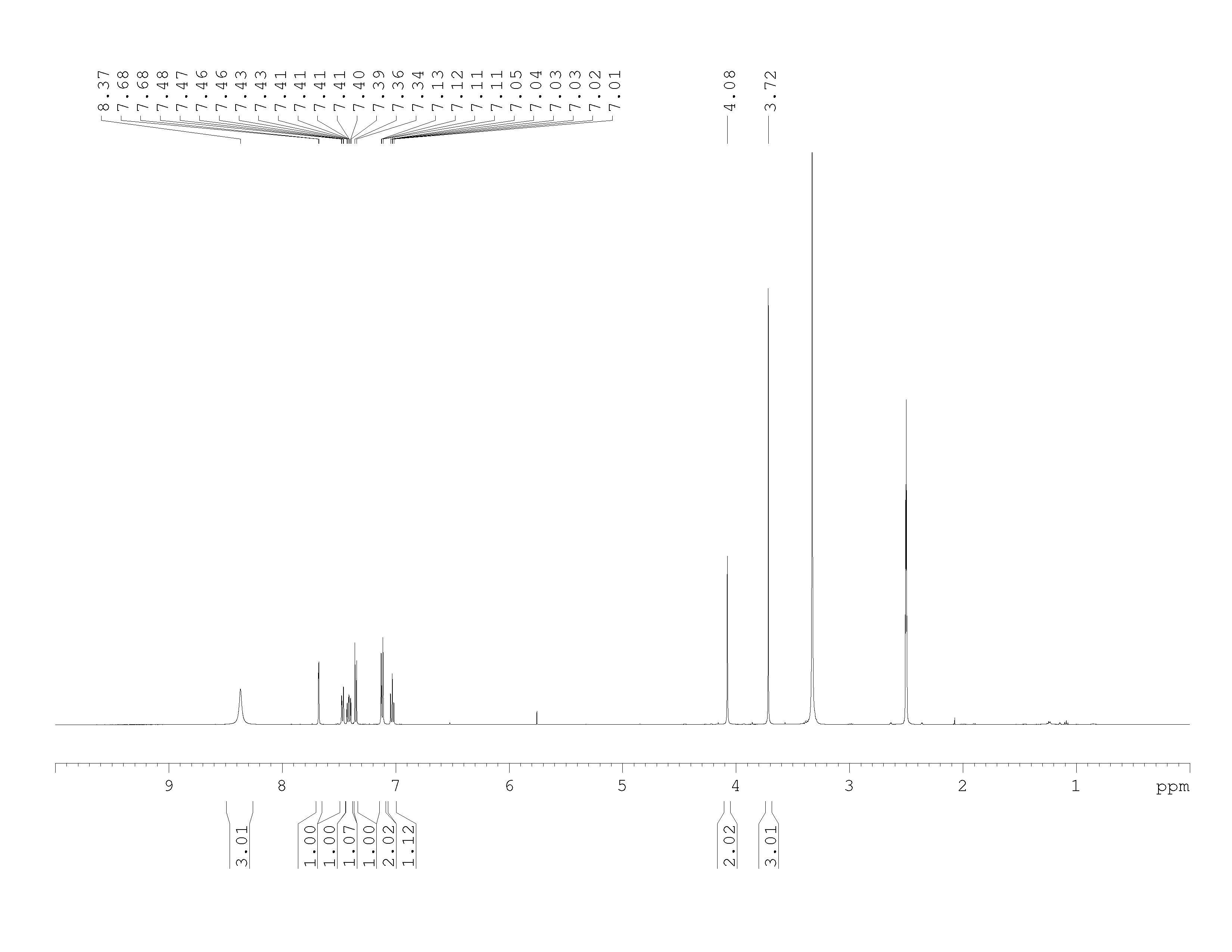

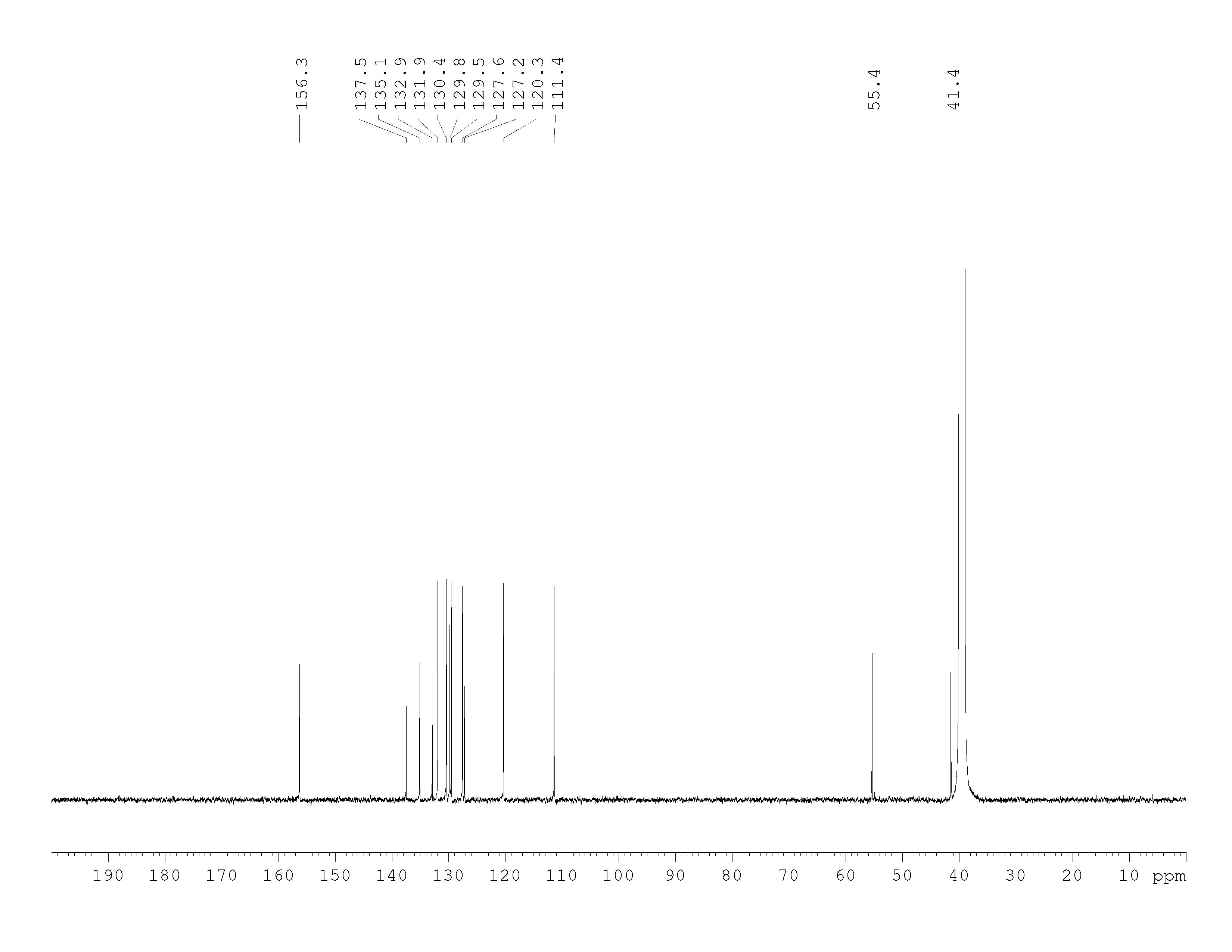


2-(1-((3,4-Dichlorobenzyl)amino)ethyl)butane-1,4-diol


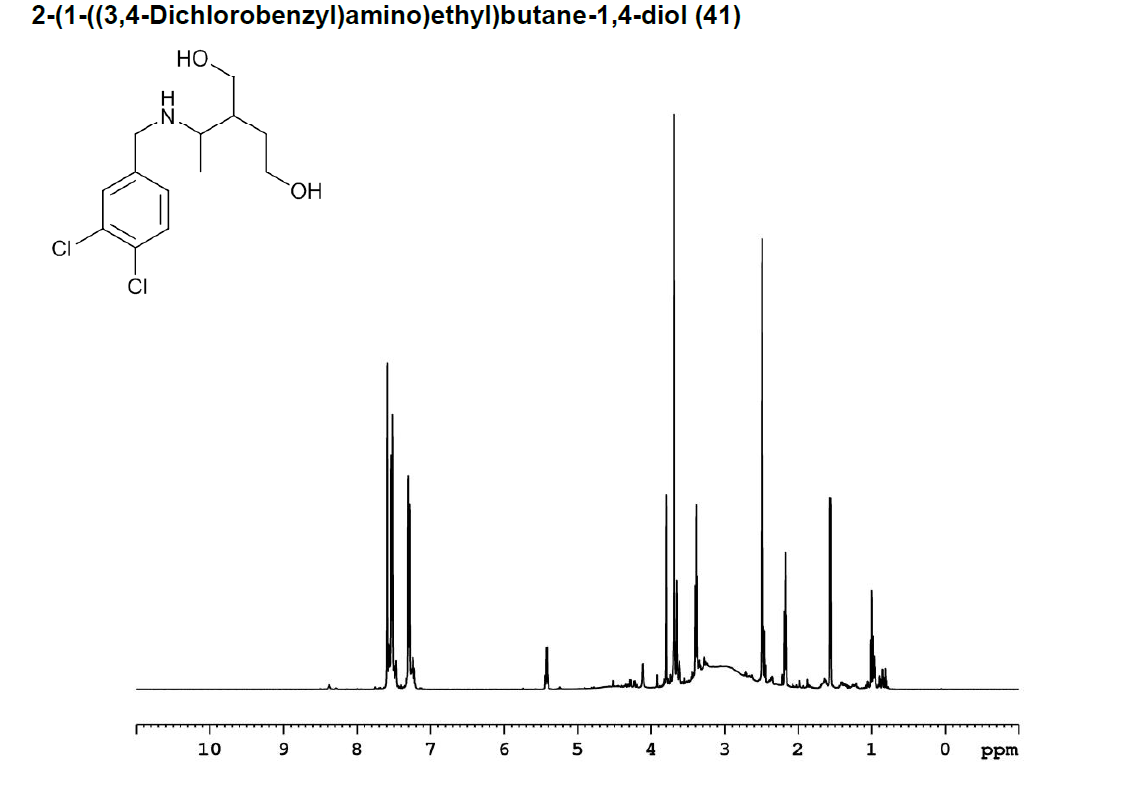

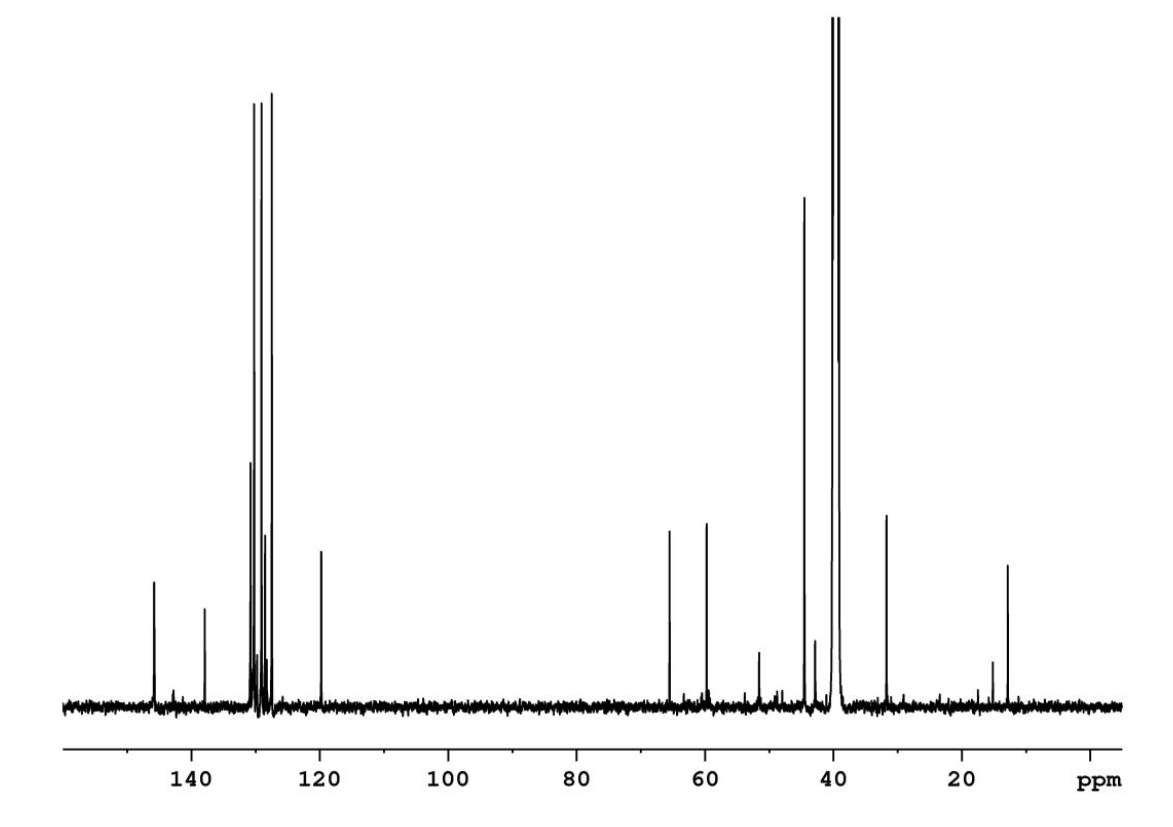


1-(3,4-dichlorophenyl)-N-methylmethanamine


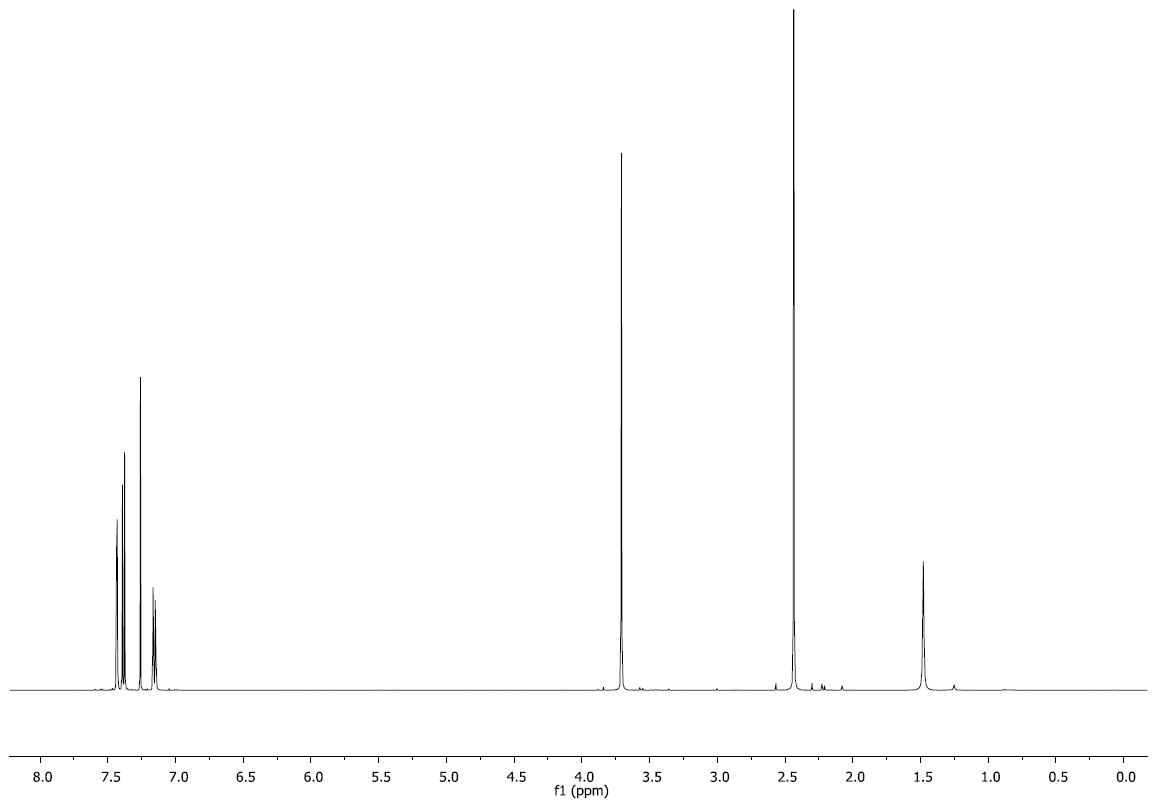


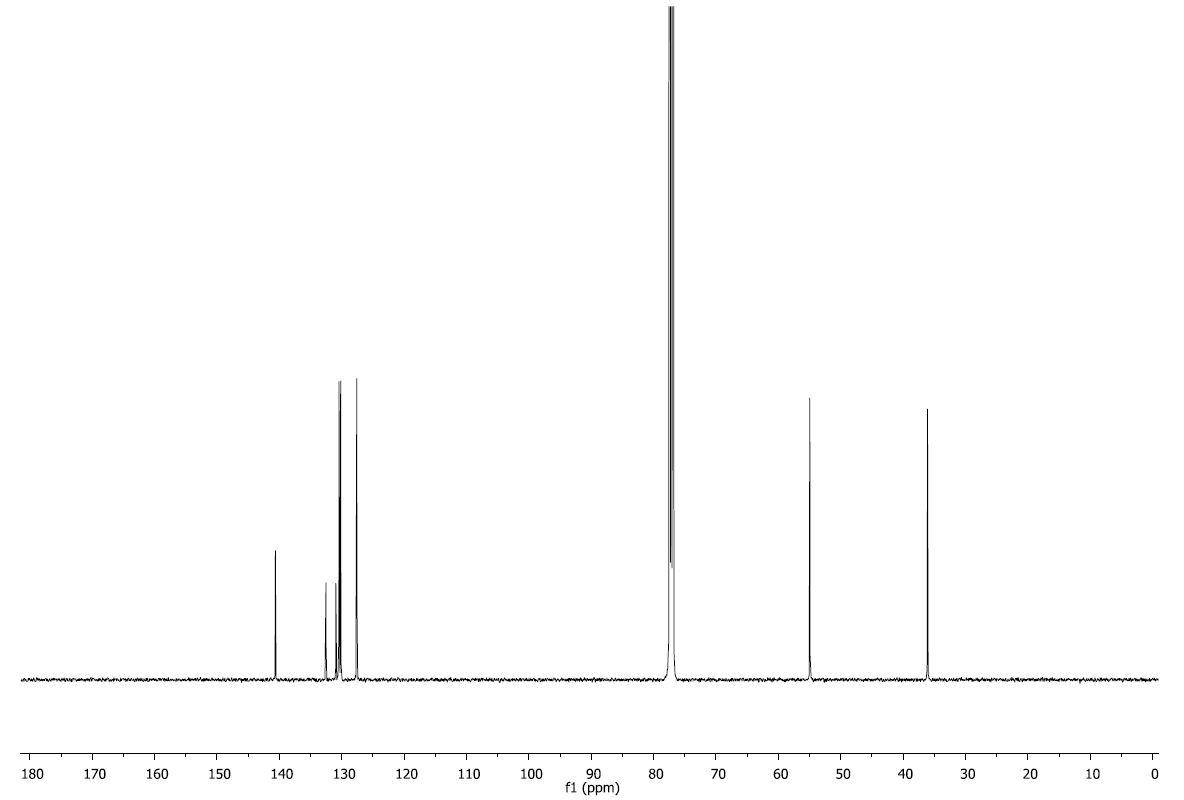


(1s,4s)-4-((3,4-dichlorobenzyl)amino)cyclohexan-1-ol


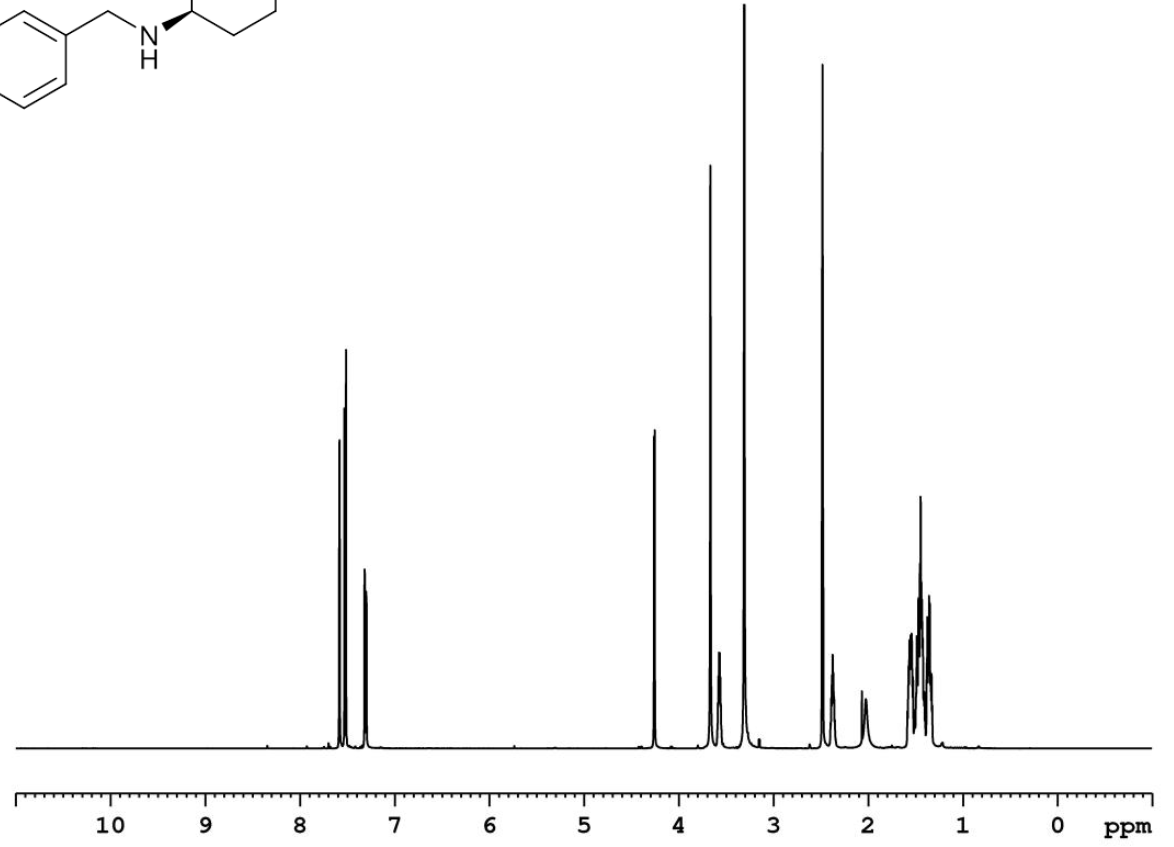


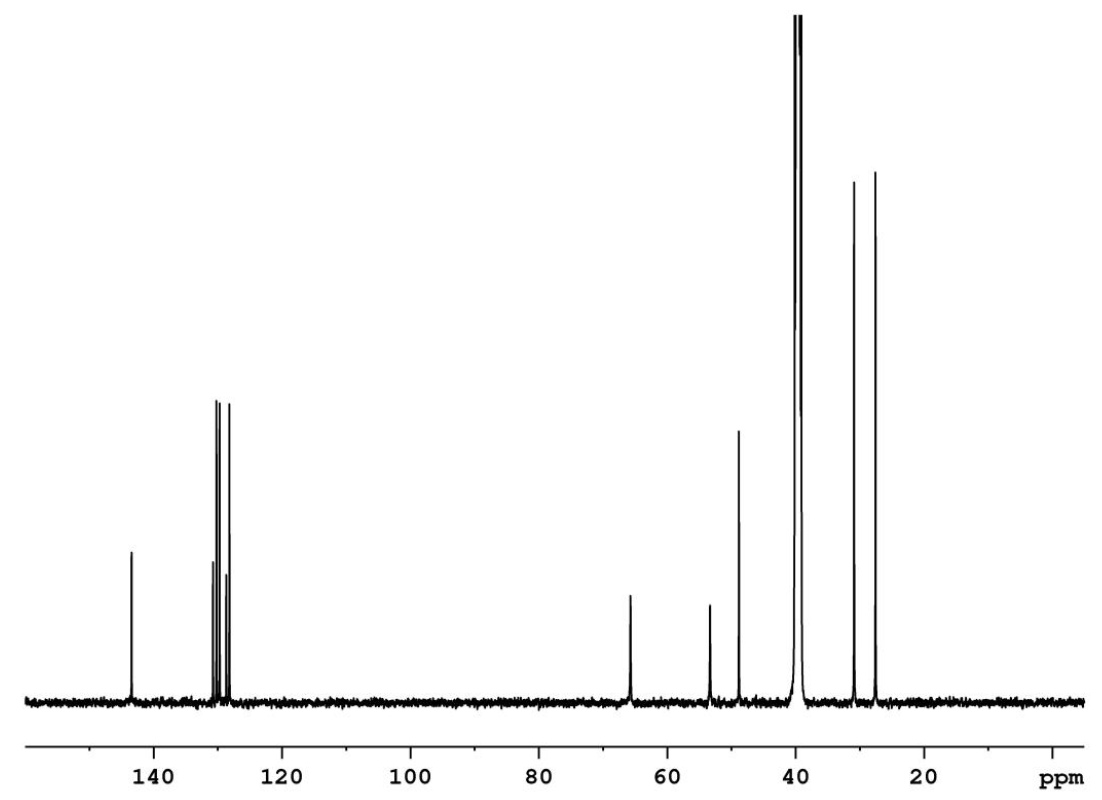


1-(2-((3,4-dichlorobenzyl)amino)ethyl)imidazolidin-2-one


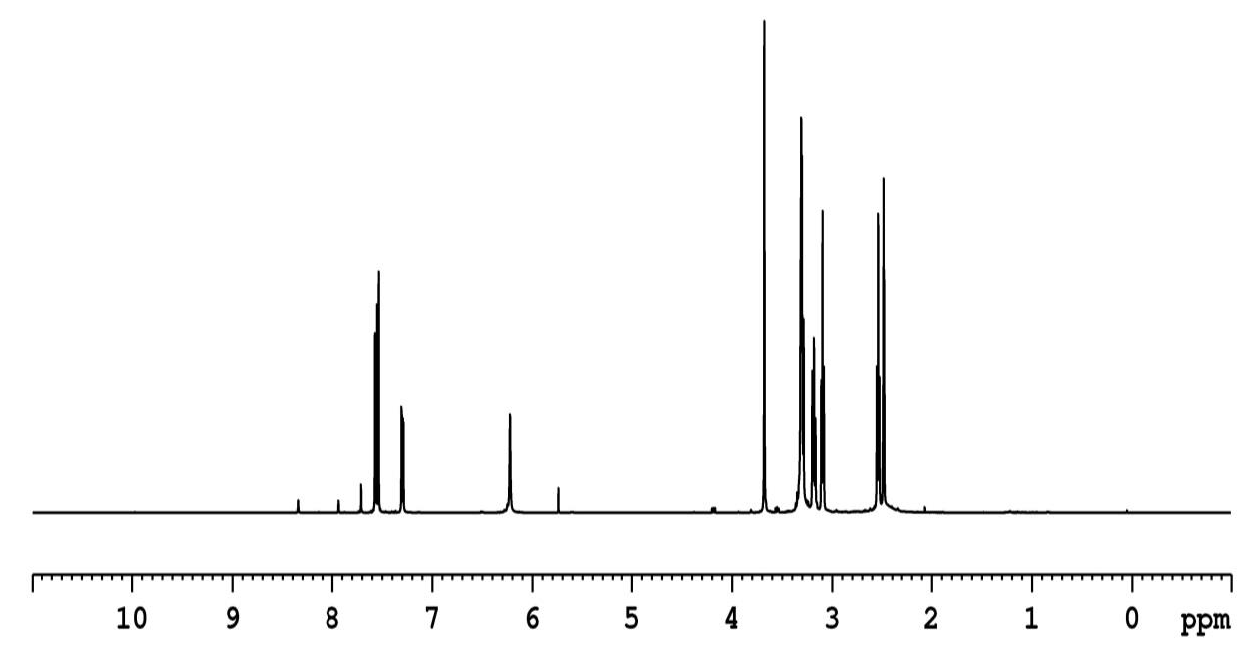

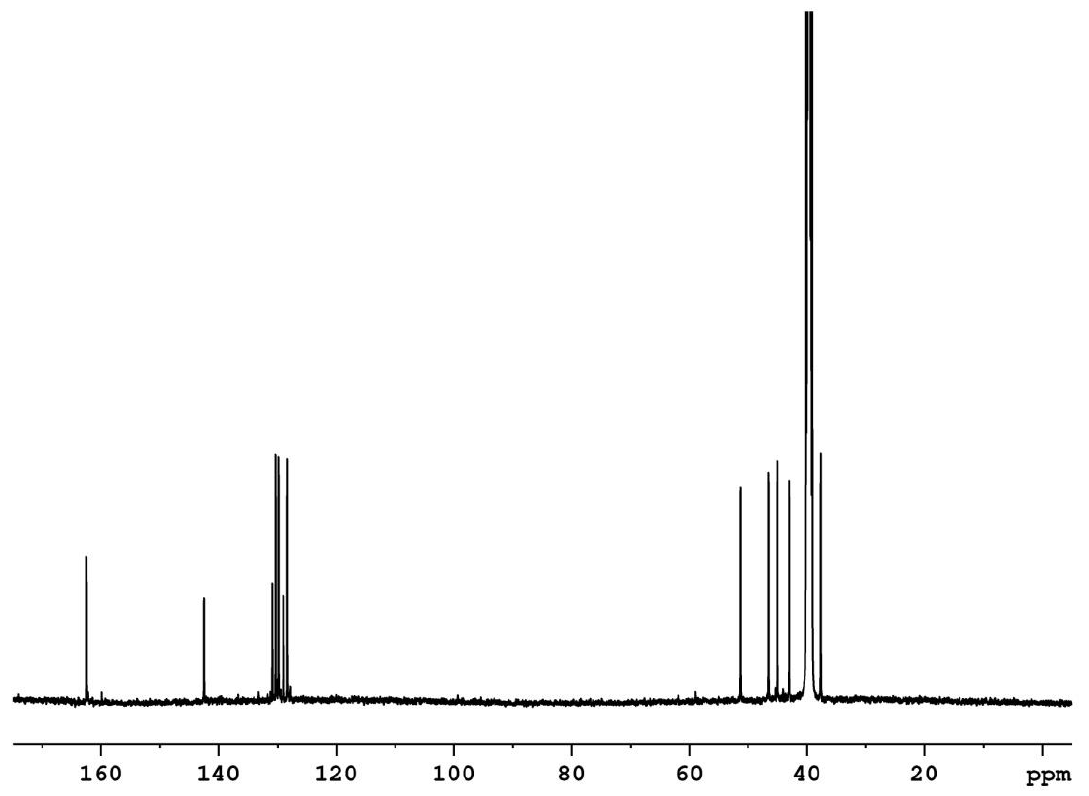


(5-(((3,4-dichlorobenzyl)amino)methyl)furan-2-yl)methanol


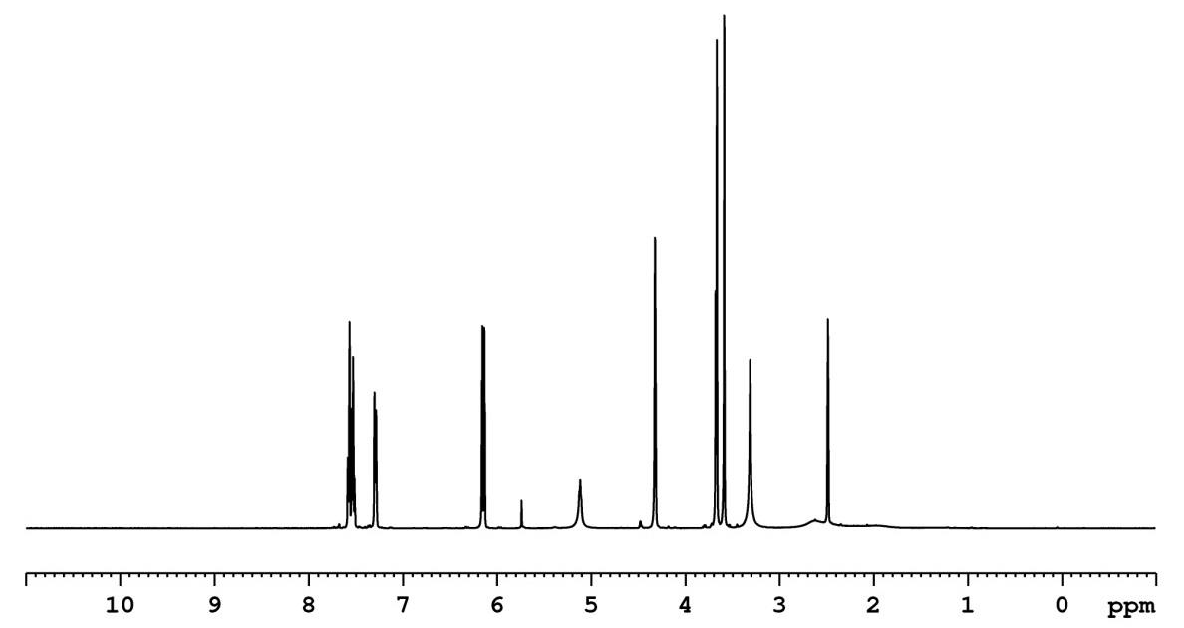


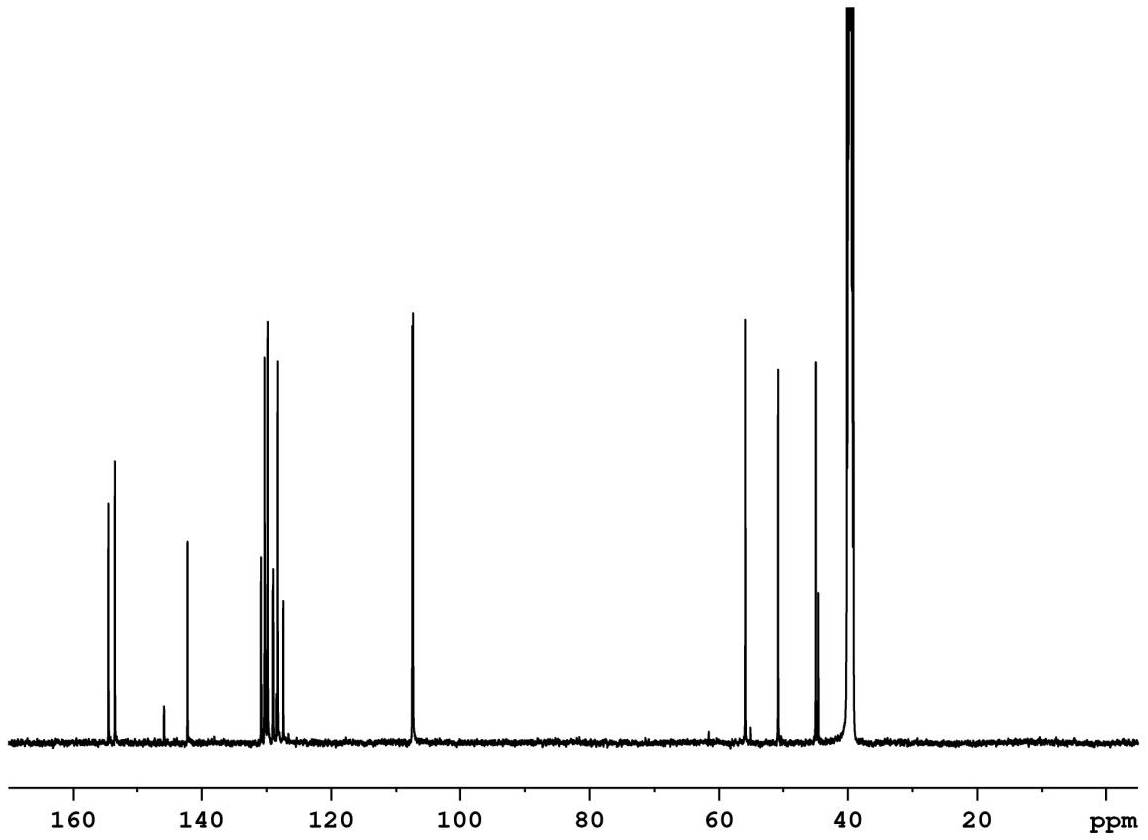


N^1^-(3,4-dichlorobenzyl)-N^3^,N^3^-dimethylpropane-1,3-diamine


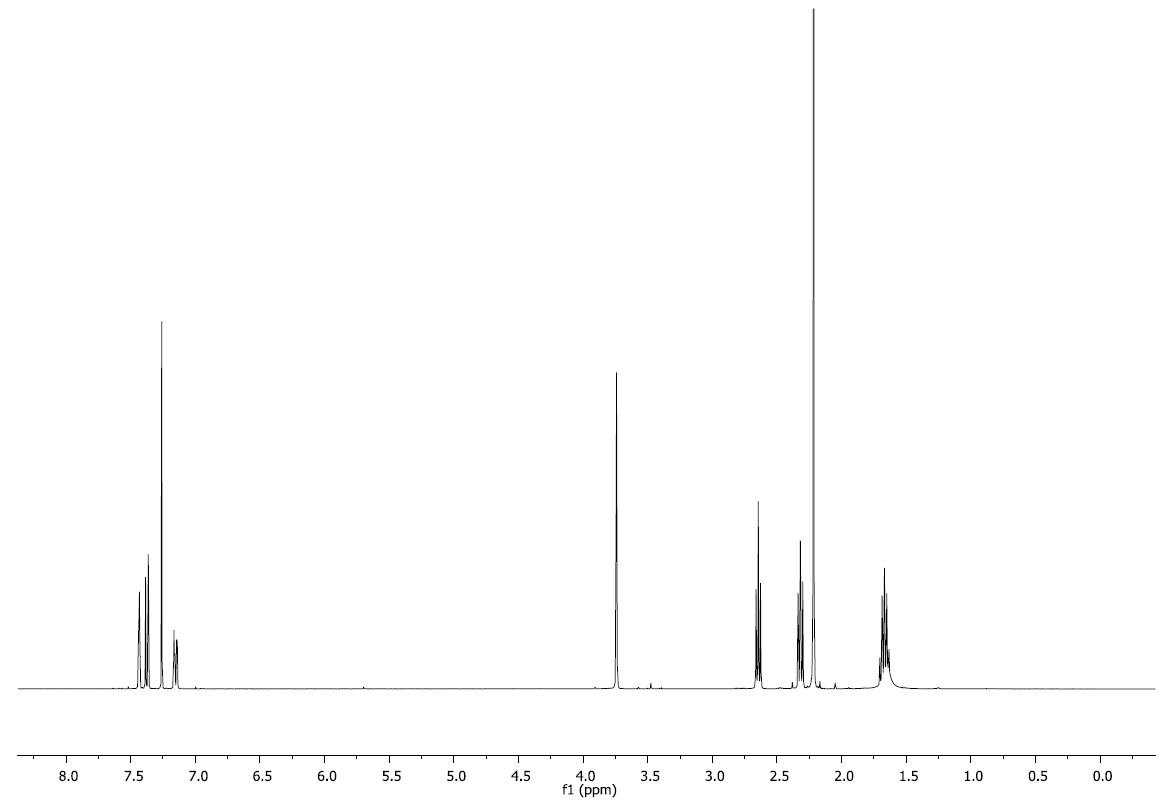


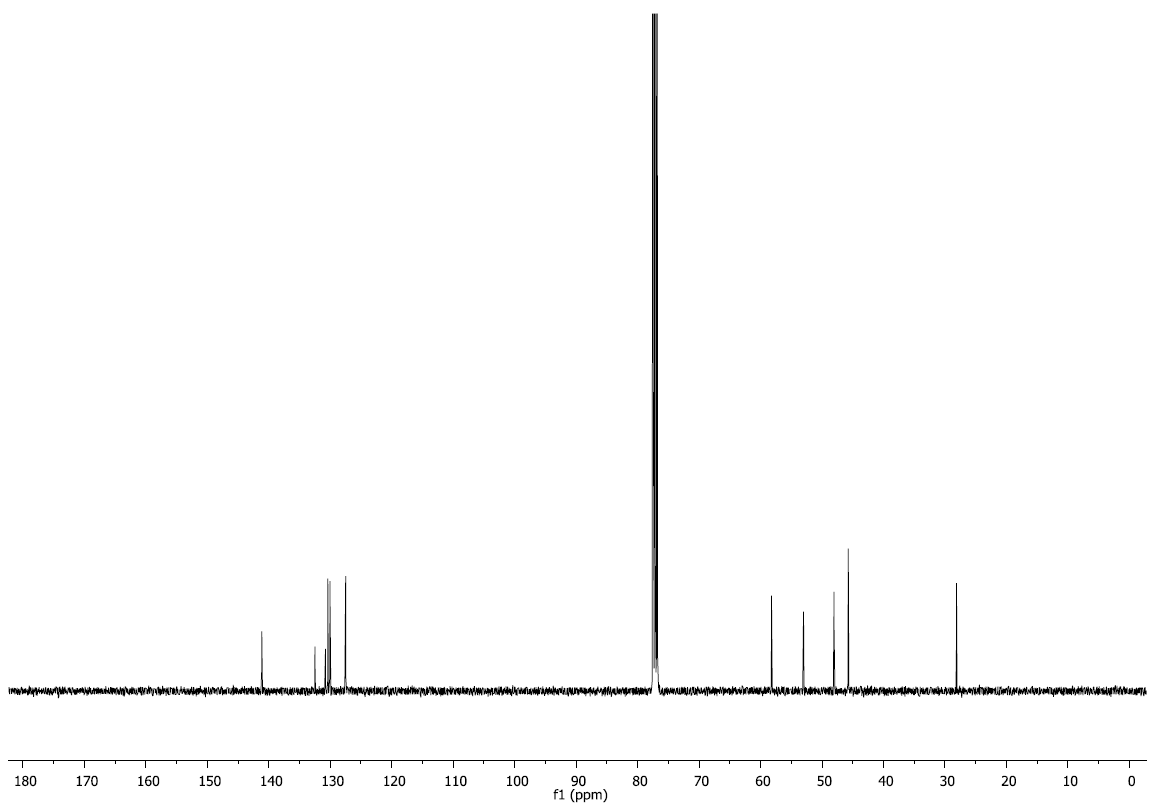


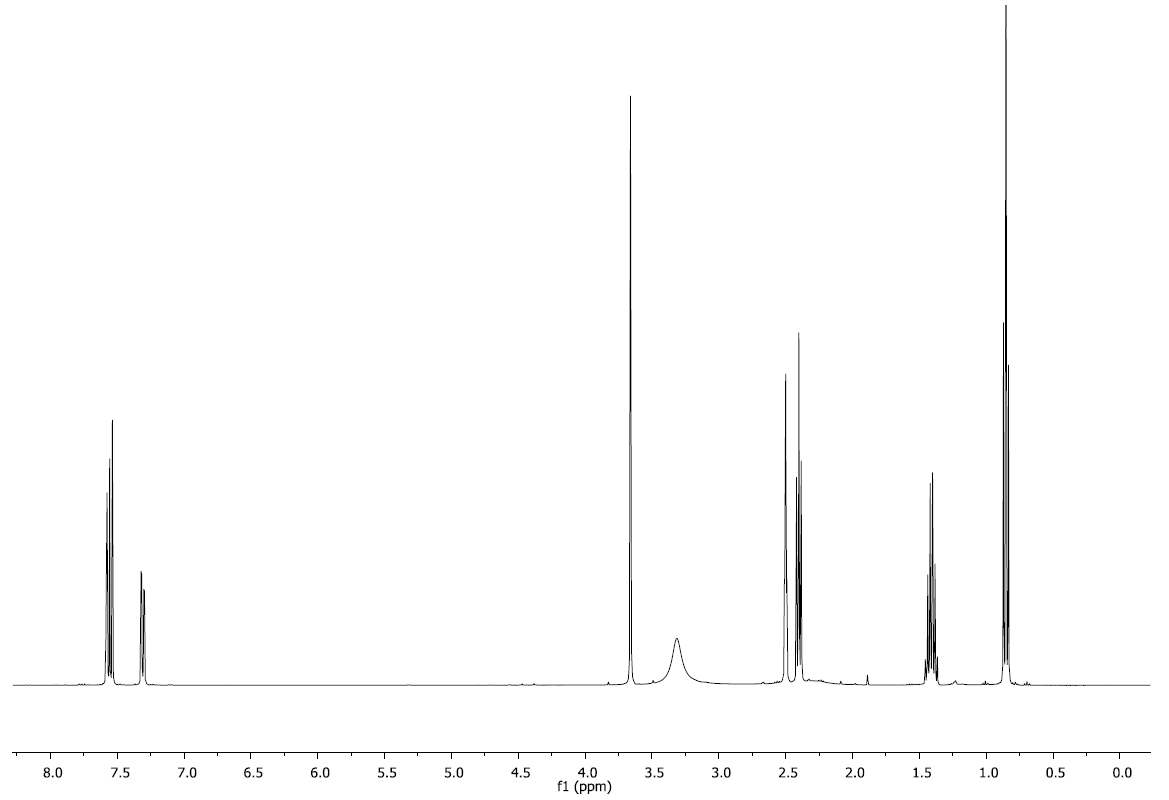
N-(3,4-dichlorobenzyl)propan-1-amine


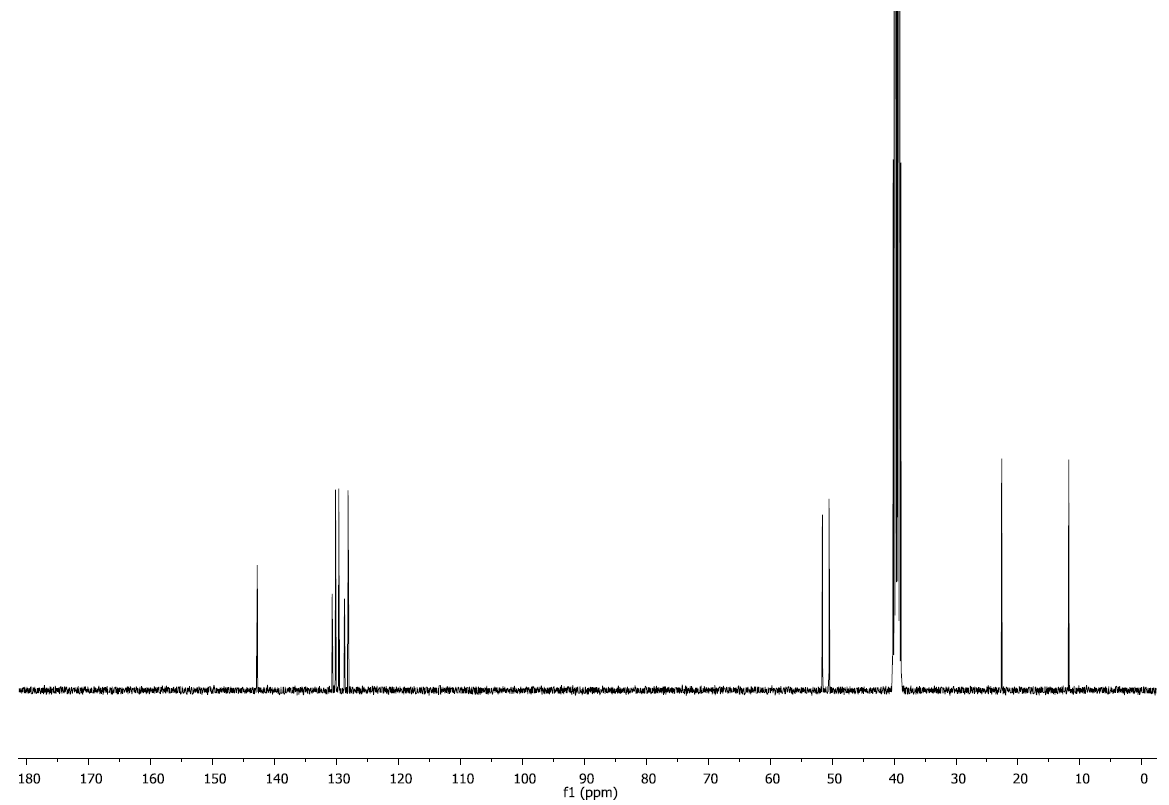


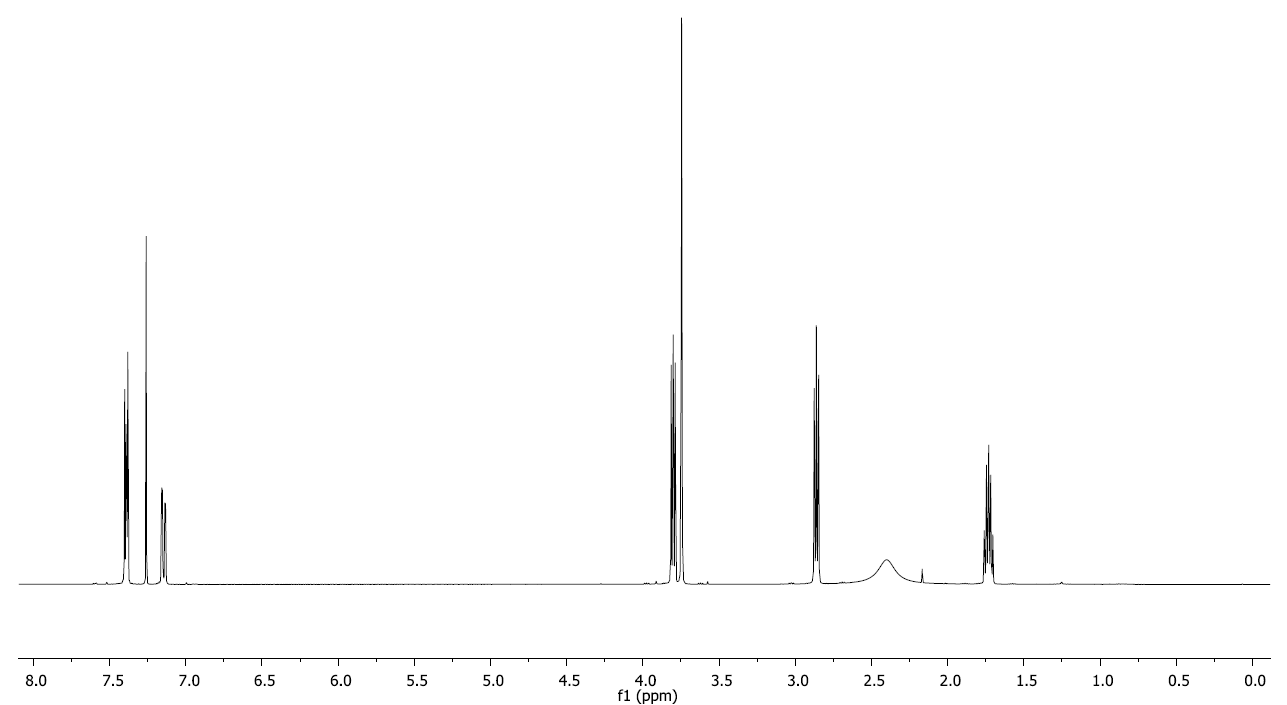
3-((3,4-dichlorobenzyl)amino)propan-1-ol


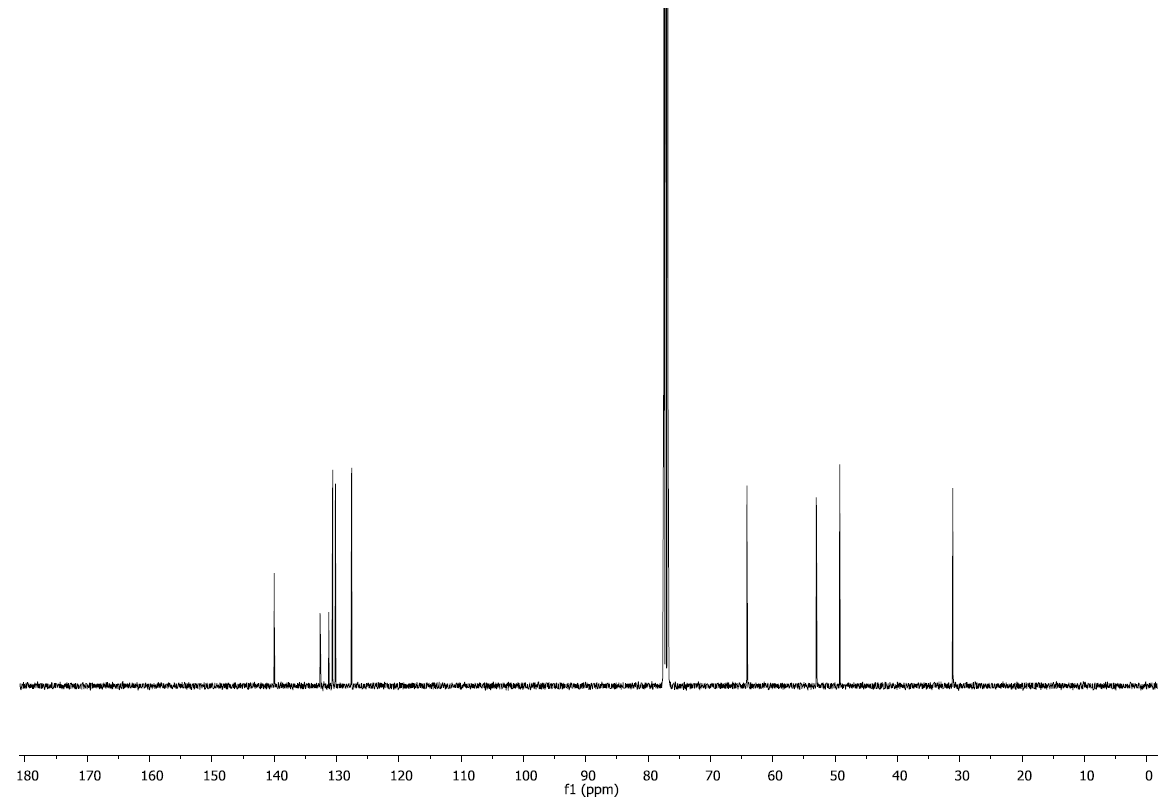


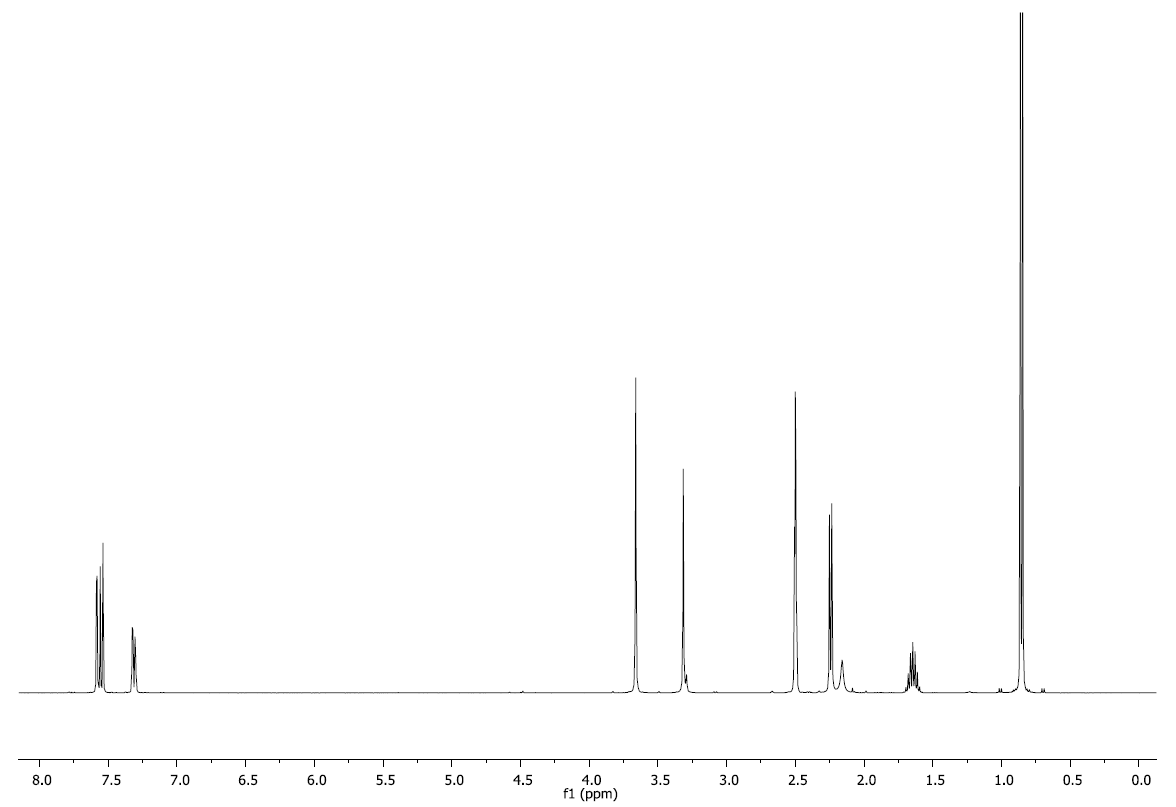
N-(3,4-dichlorobenzyl)-2-methylpropan-1-amine


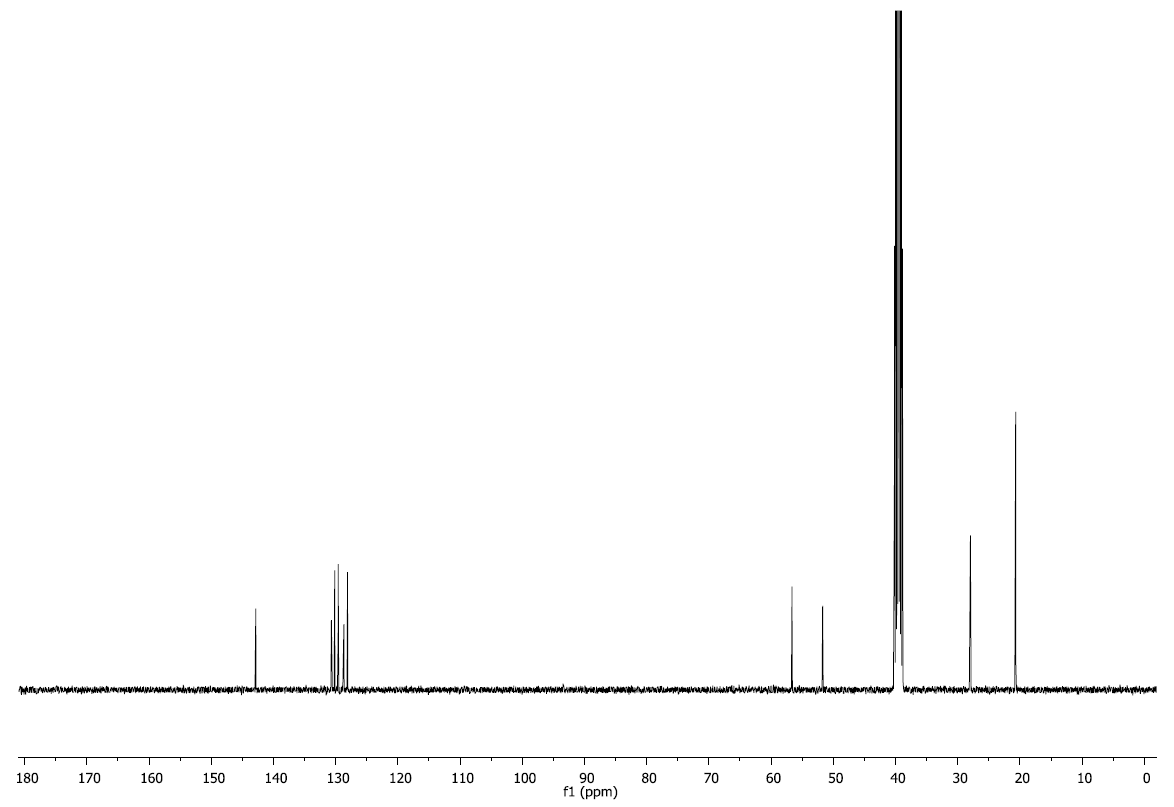


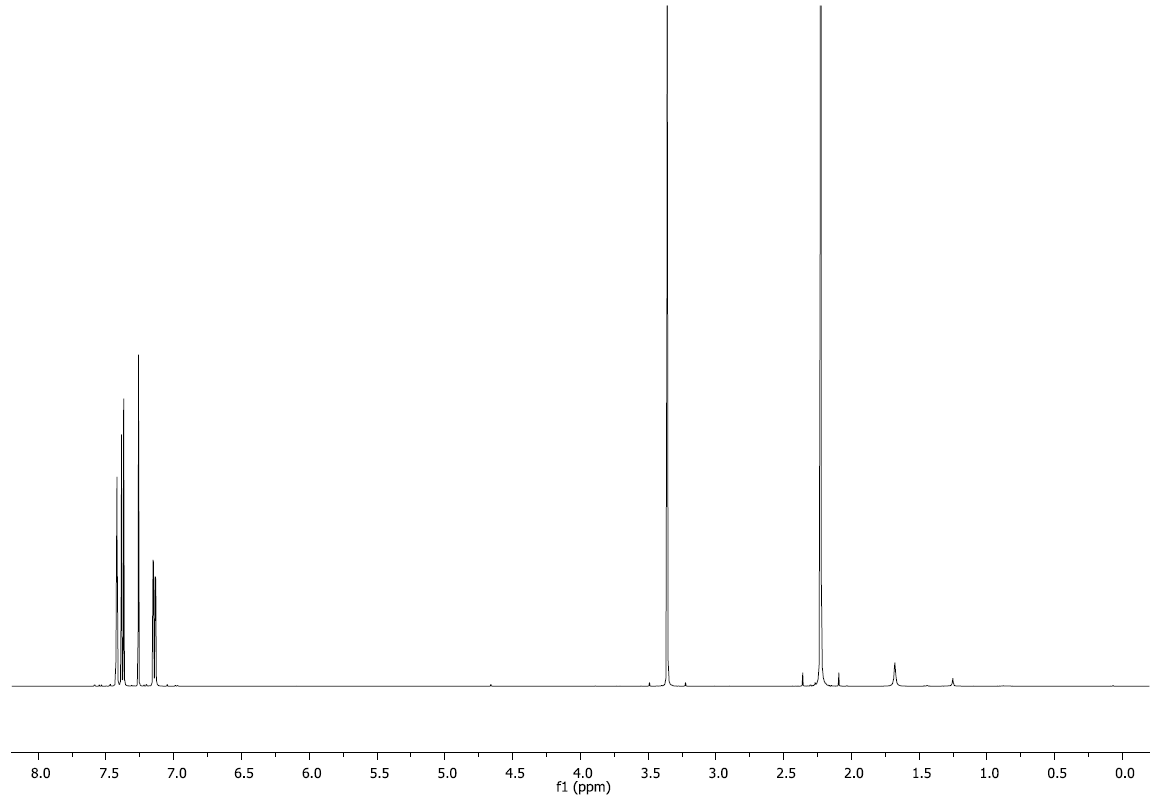
1-(3,4-dichlorophenyl)-N,N-dimethylmethanamine


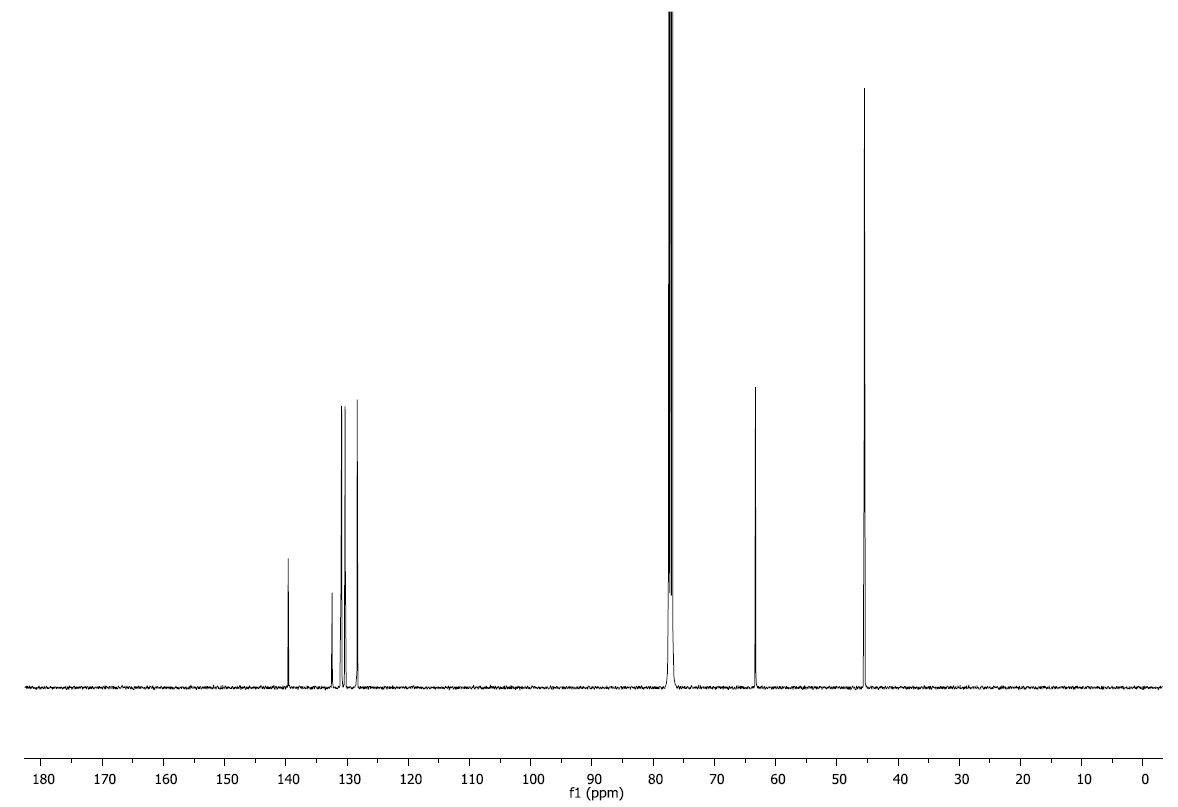


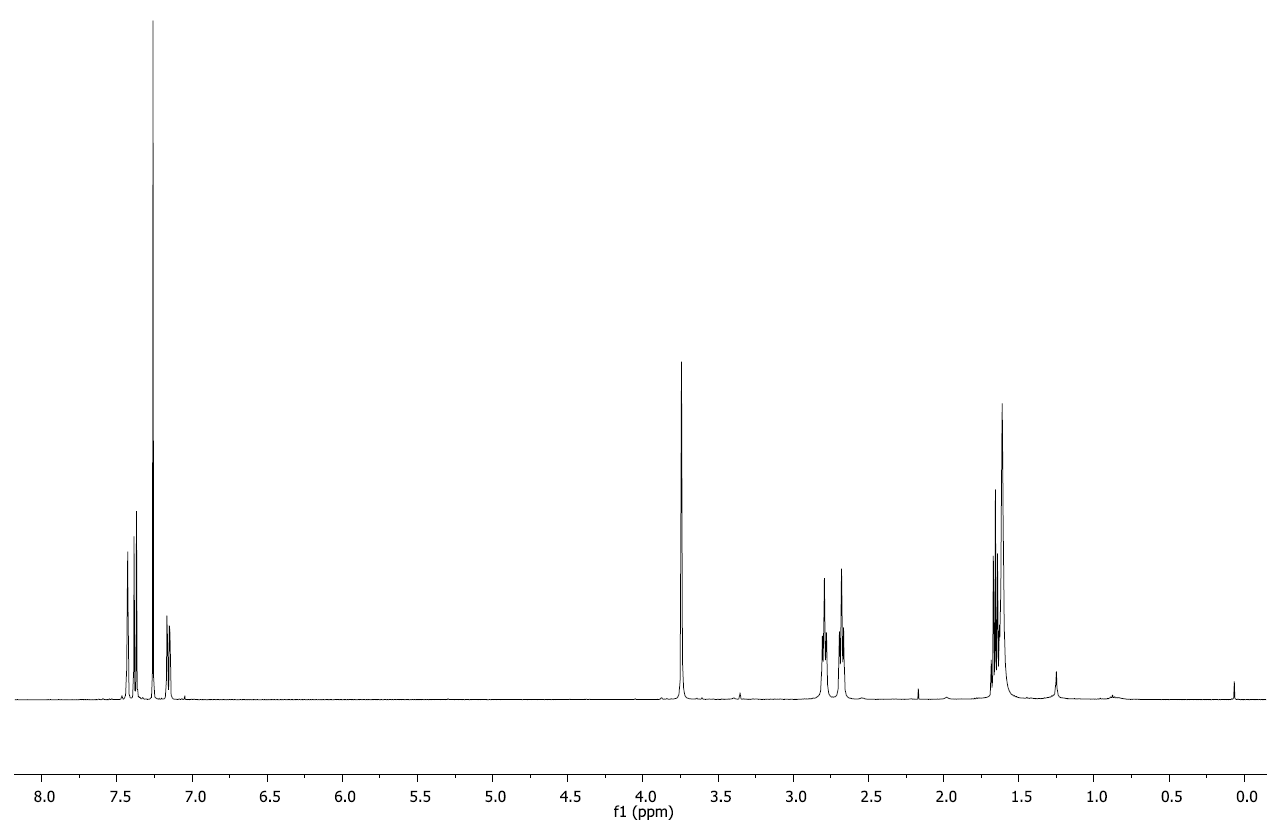
N^1^-(3,4-dichlorobenzyl)propane-1,3-diamine


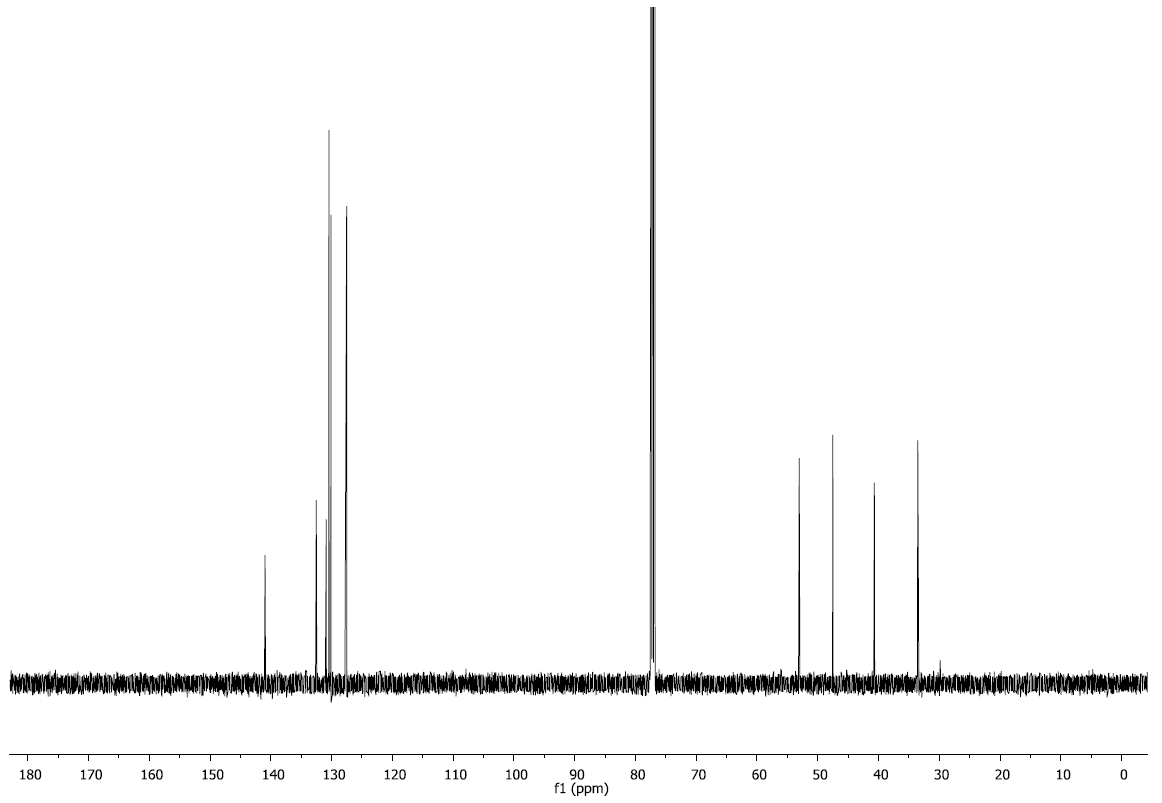


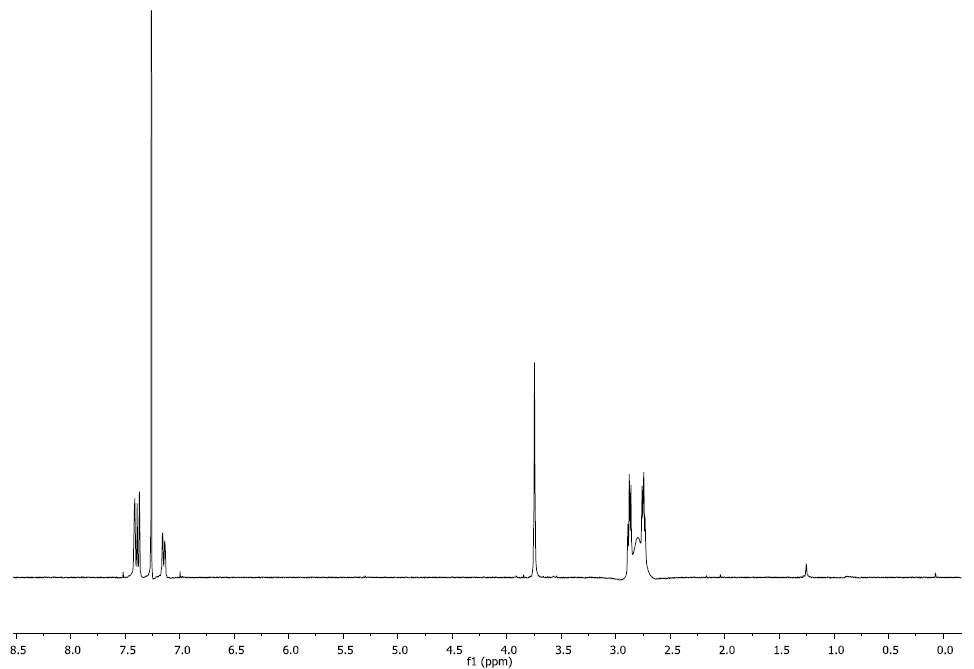
N^1^-(3,4-dichlorobenzyl)ethane-1,2-diamine


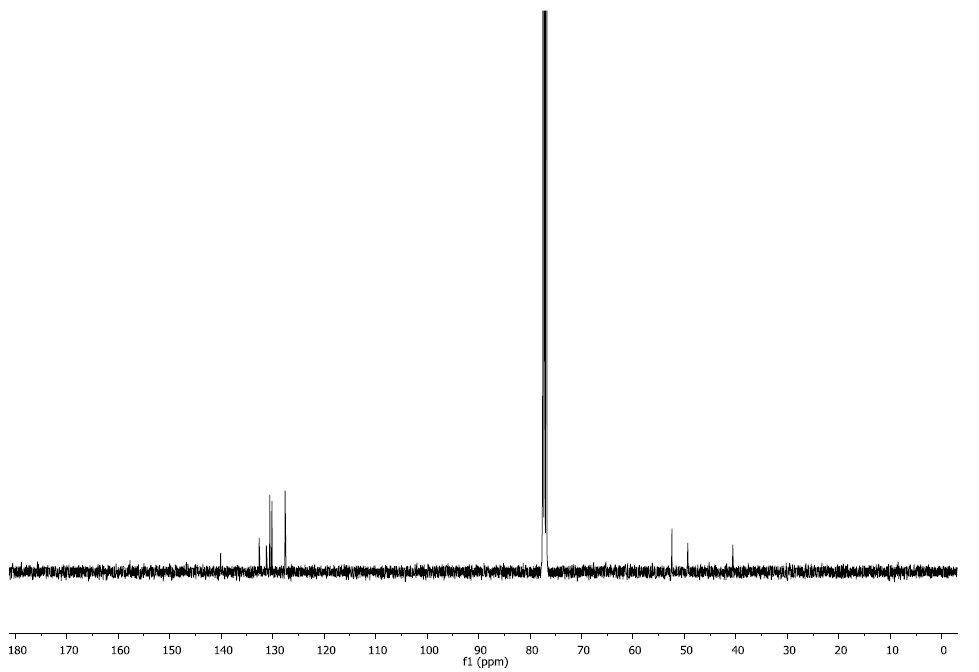


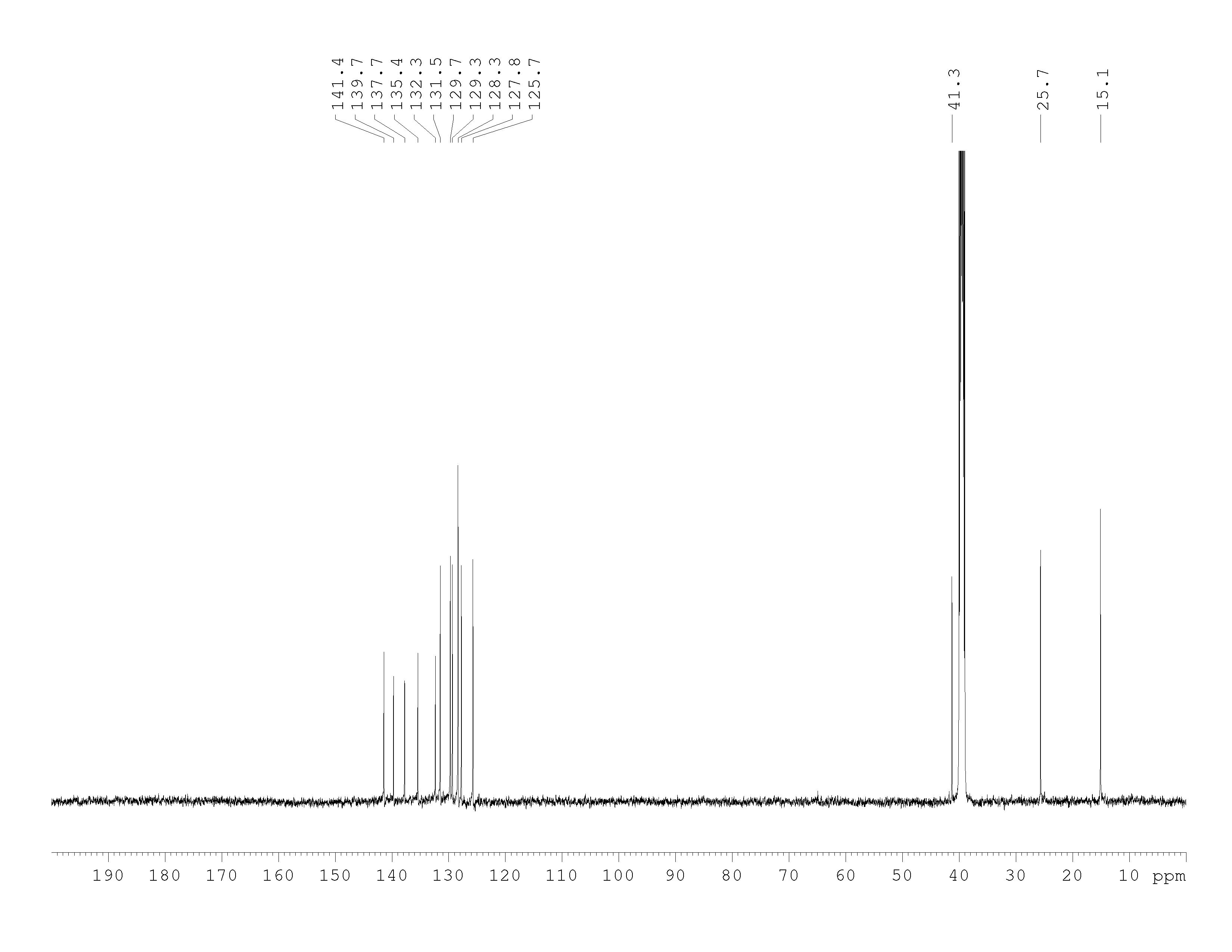

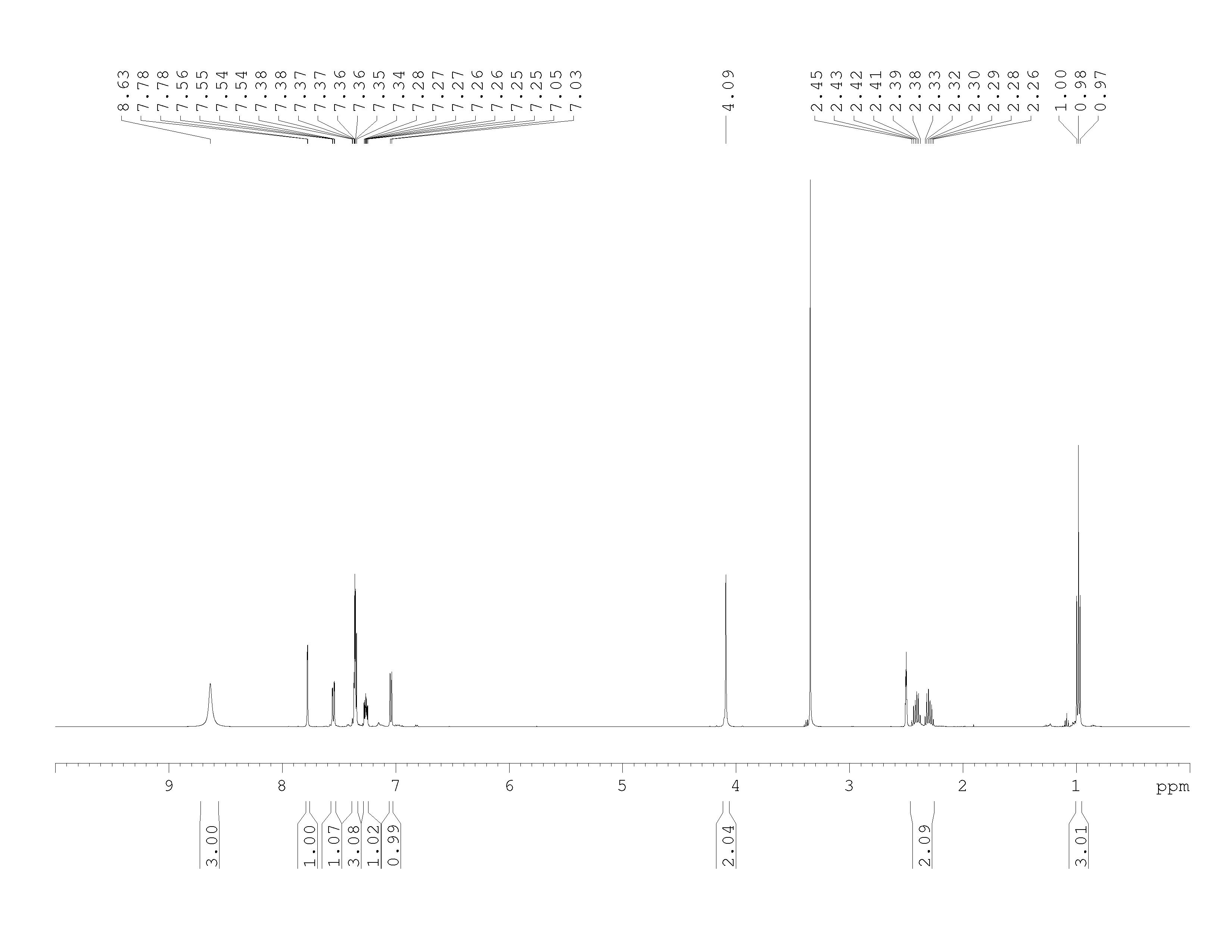
(2-chloro-2'-ethyl-[1,1'-biphenyl]-4-yl)methanamine


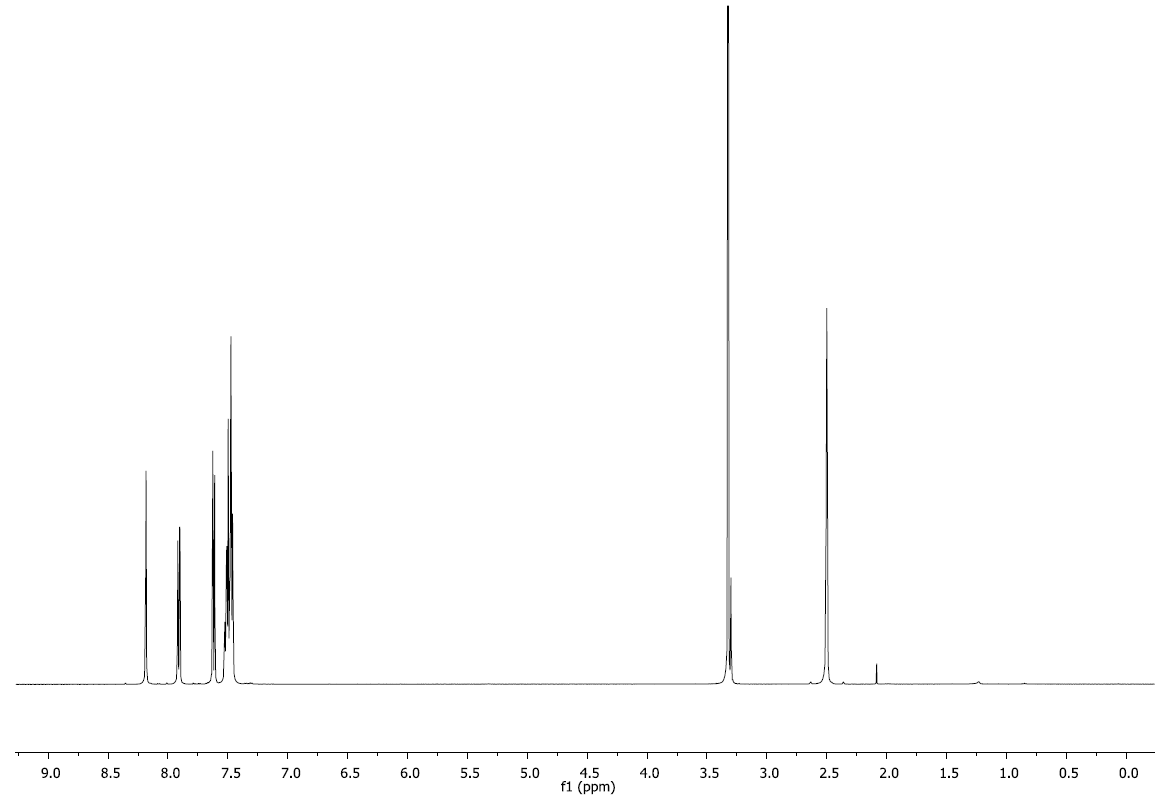
(2-chloro-[1,1'-biphenyl]-4-yl)methanamine


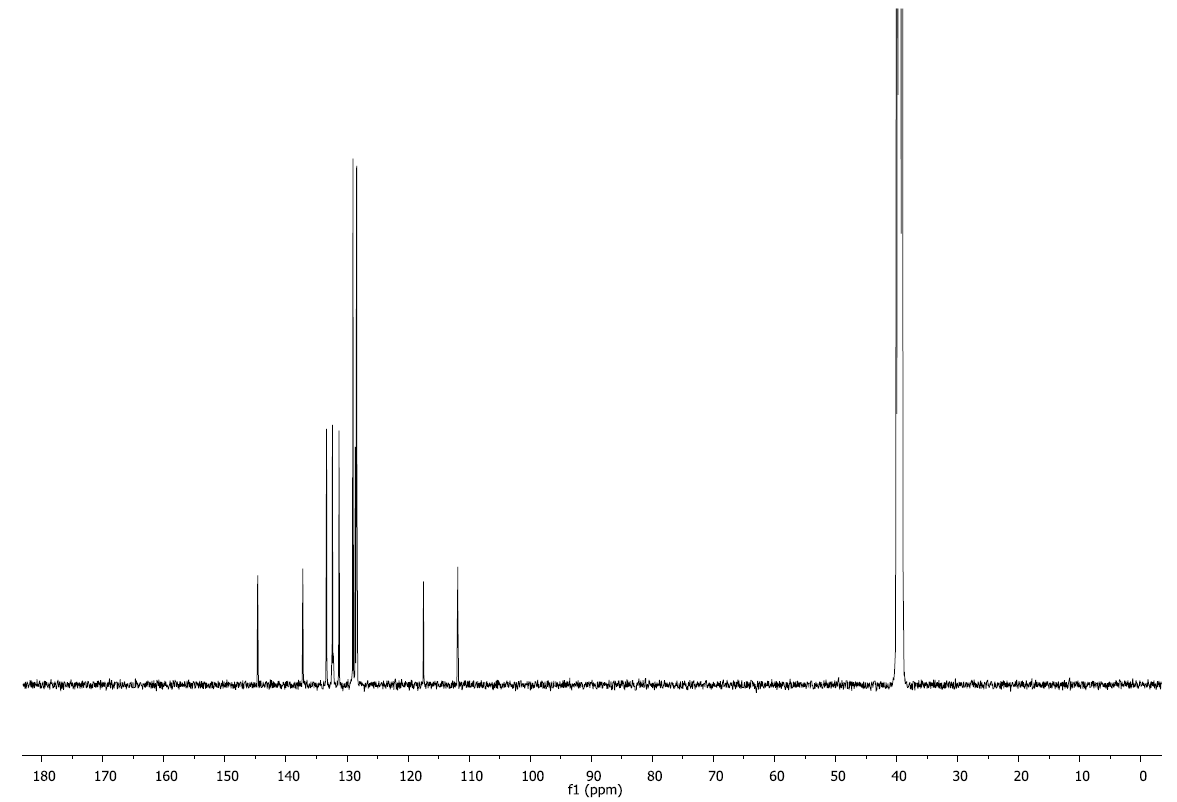


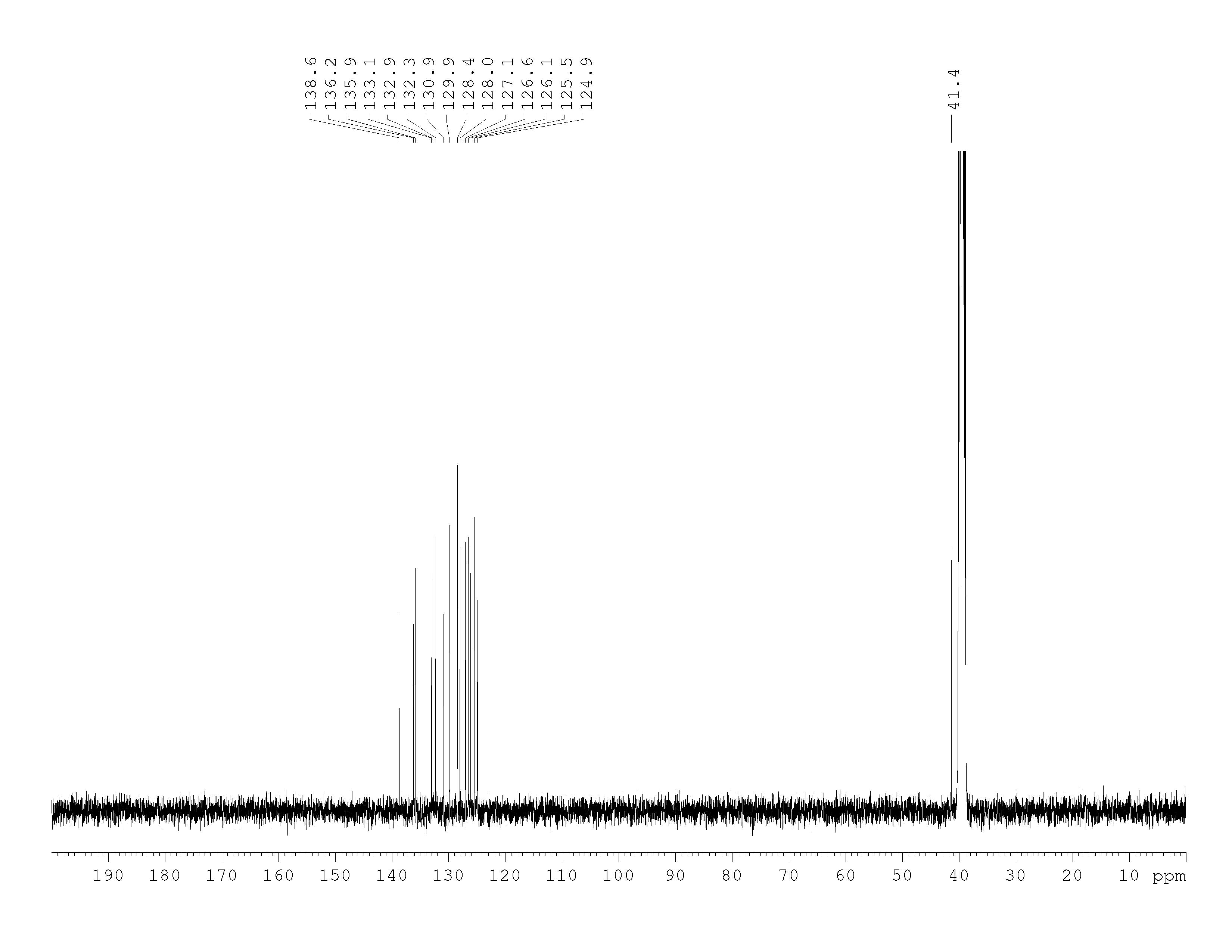

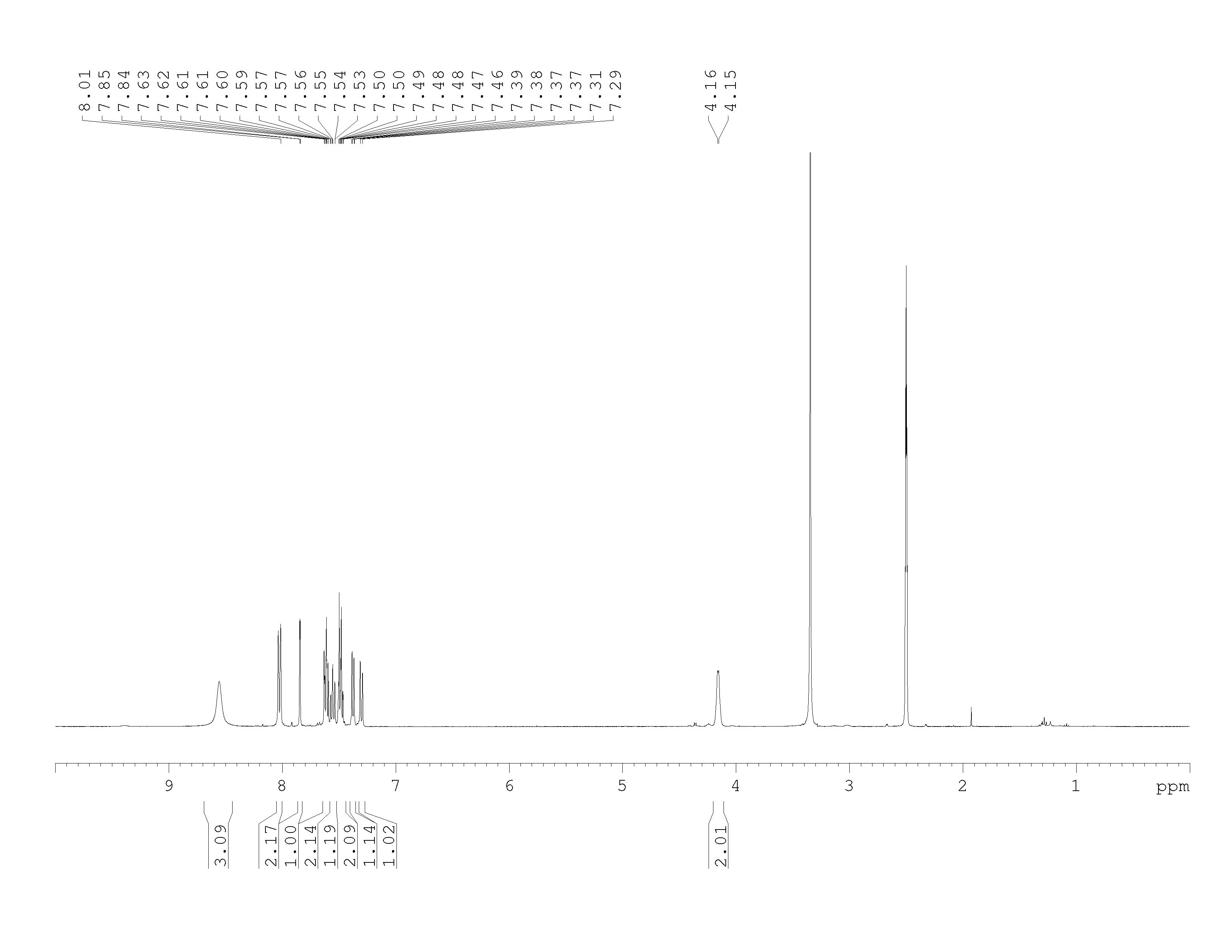
(3-chloro-4-(naphthalen-1-yl)phenyl)methanaminium

(4'-(aminomethyl)-2'-chloro-[1,1'-biphenyl]-2-yl)methanol
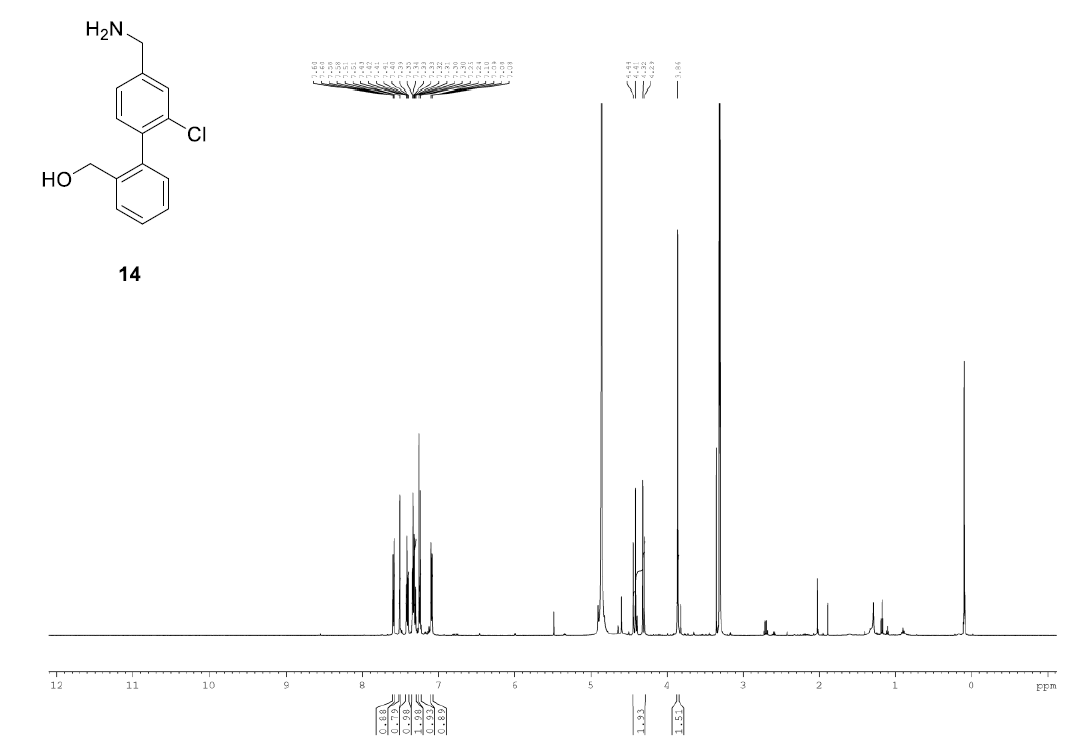


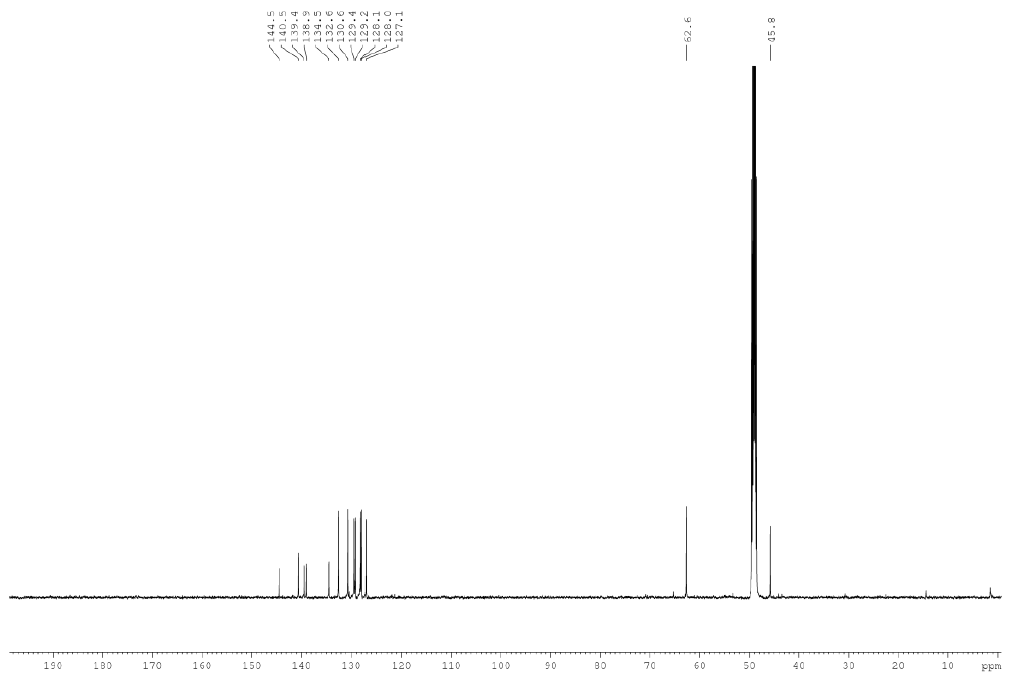


(2'-((1H-pyrrol-1-yl)methyl)-2-chloro-[1,1'-biphenyl]-4-yl)methanamine


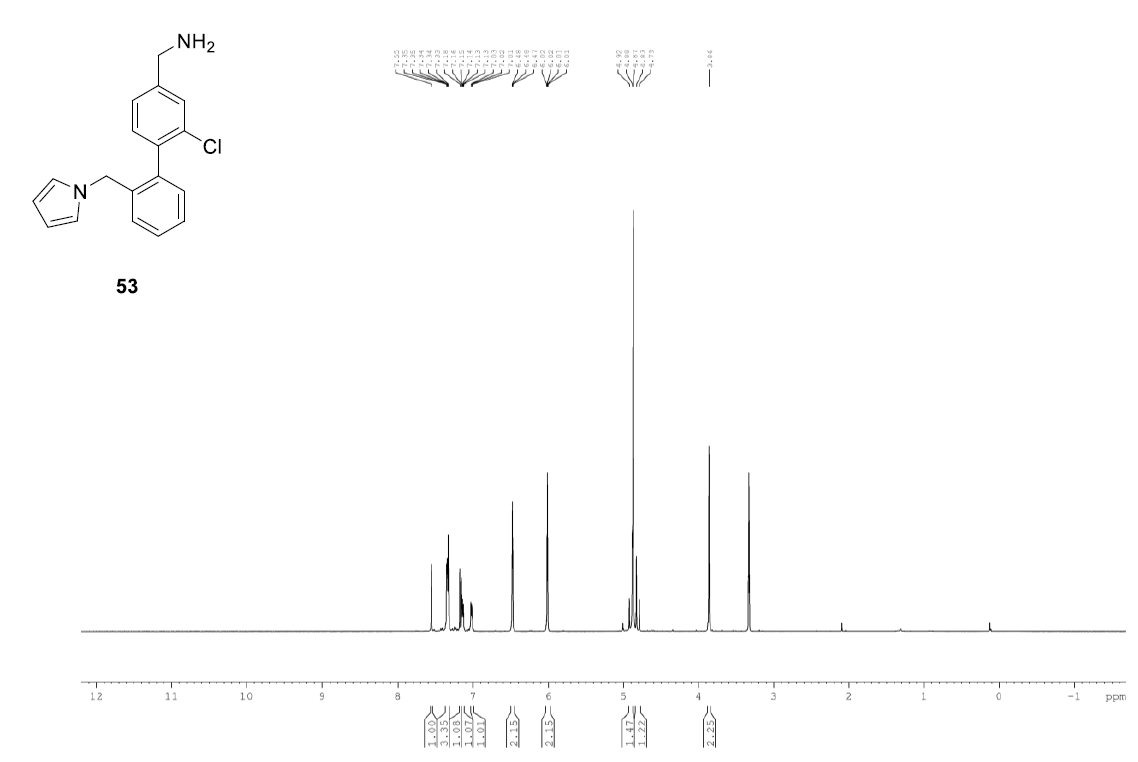


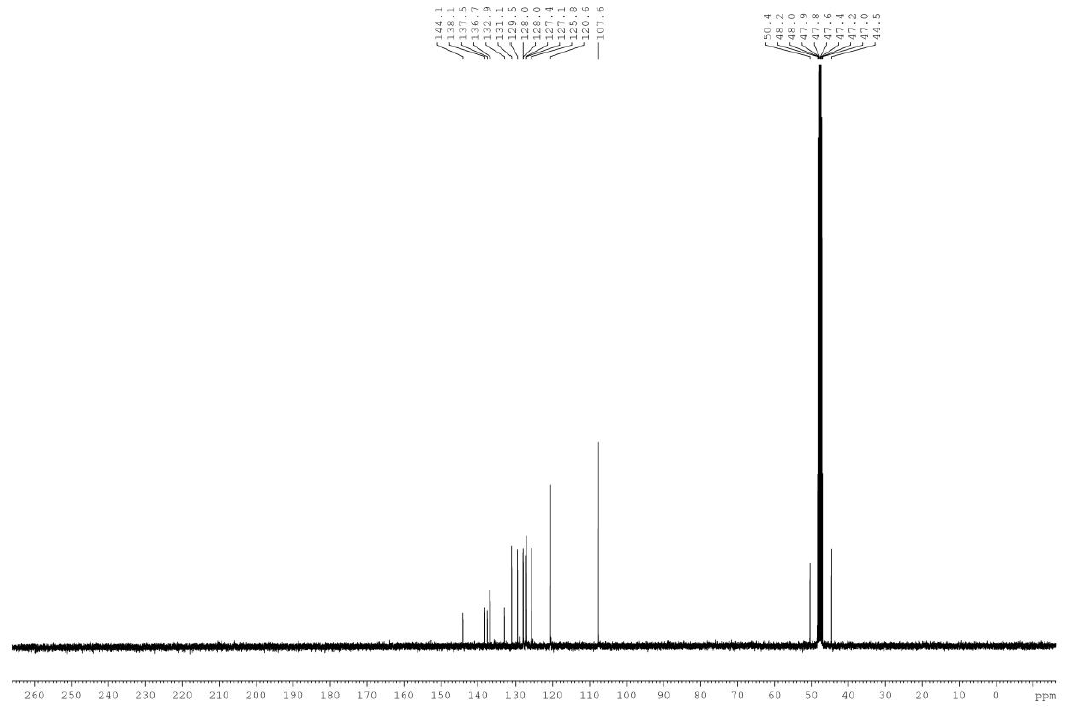


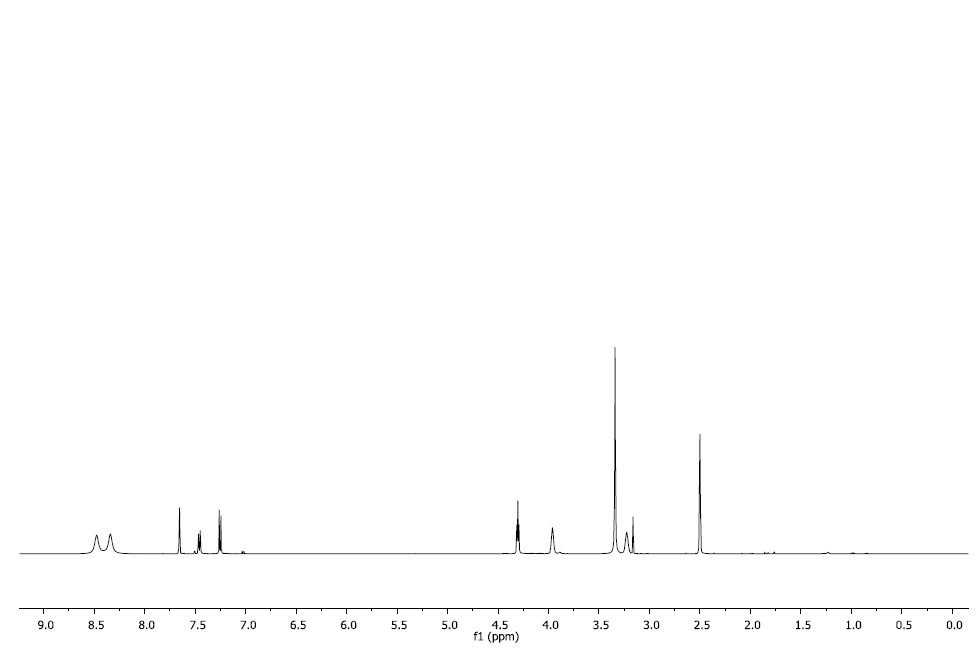
2-(4-(aminomethyl)-2-chlorophenoxy)ethan-1-amine


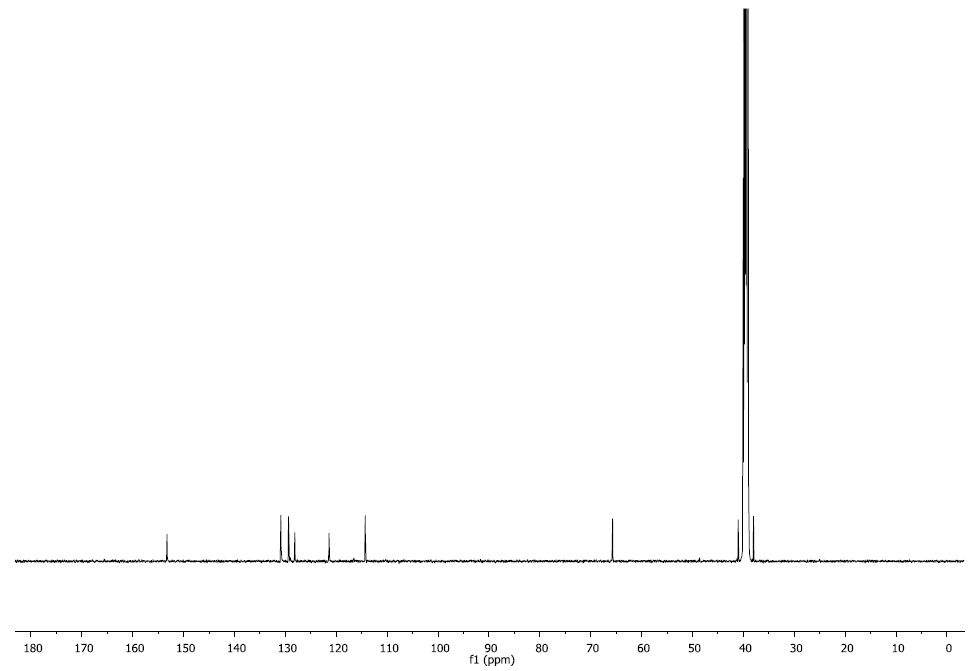


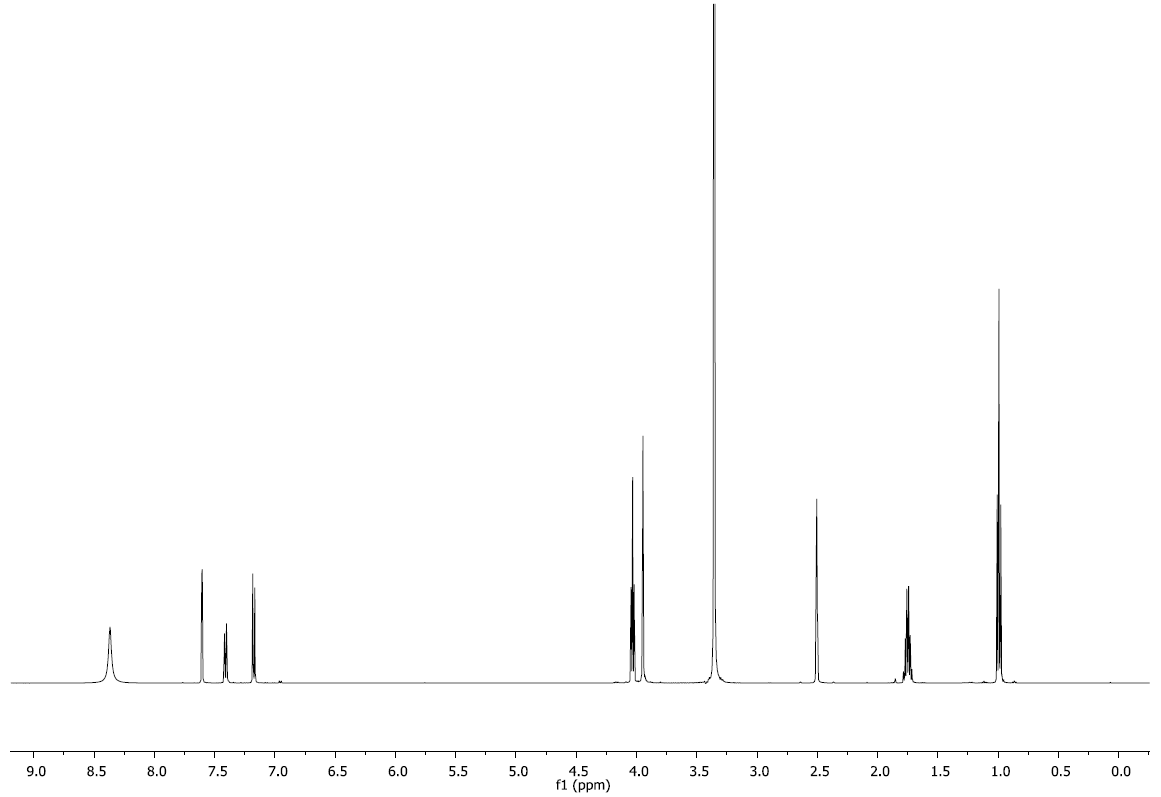
(3-chloro-4-propoxyphenyl)methanamine


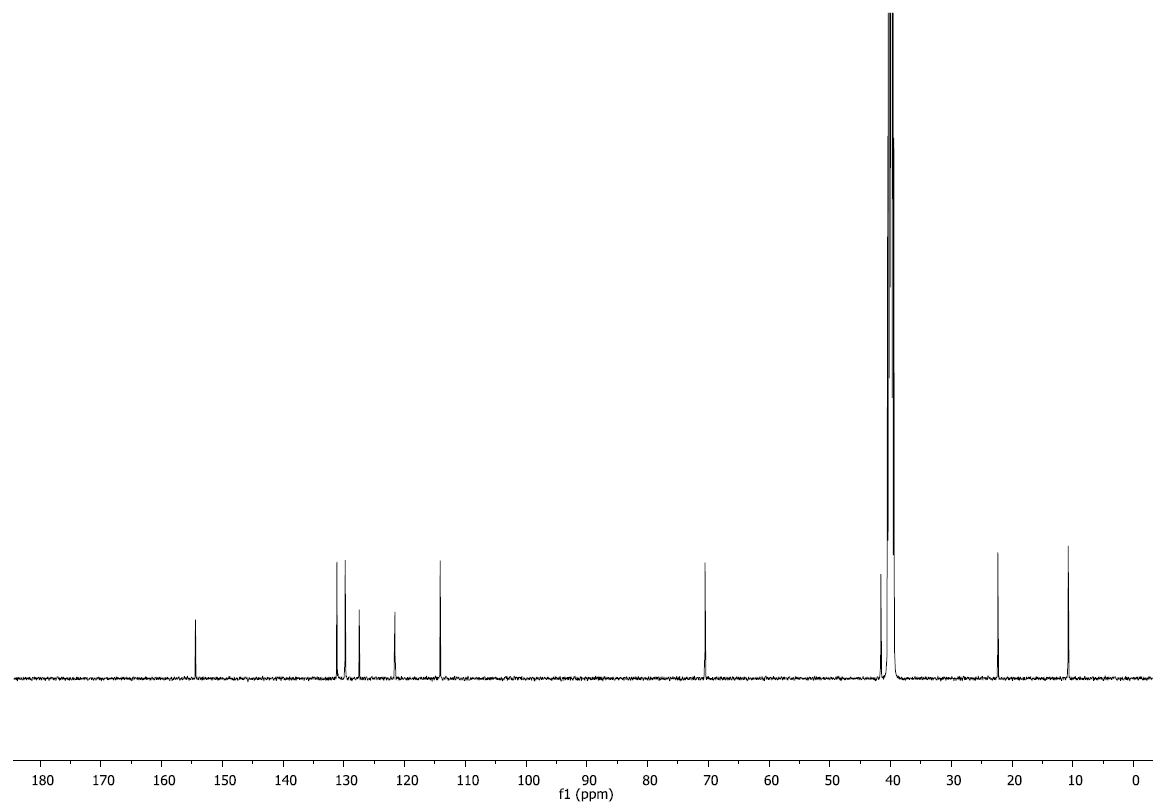


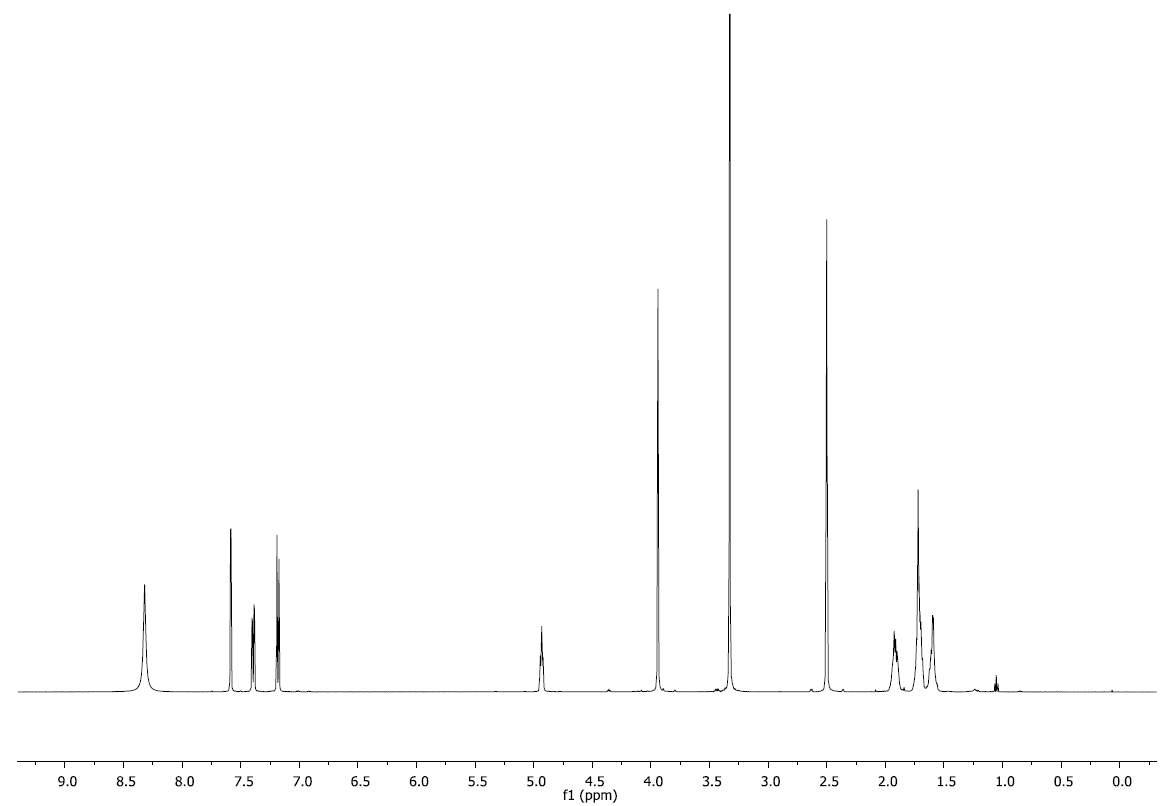
(3-chloro-4-(cyclopentyloxy)phenyl)methanamine


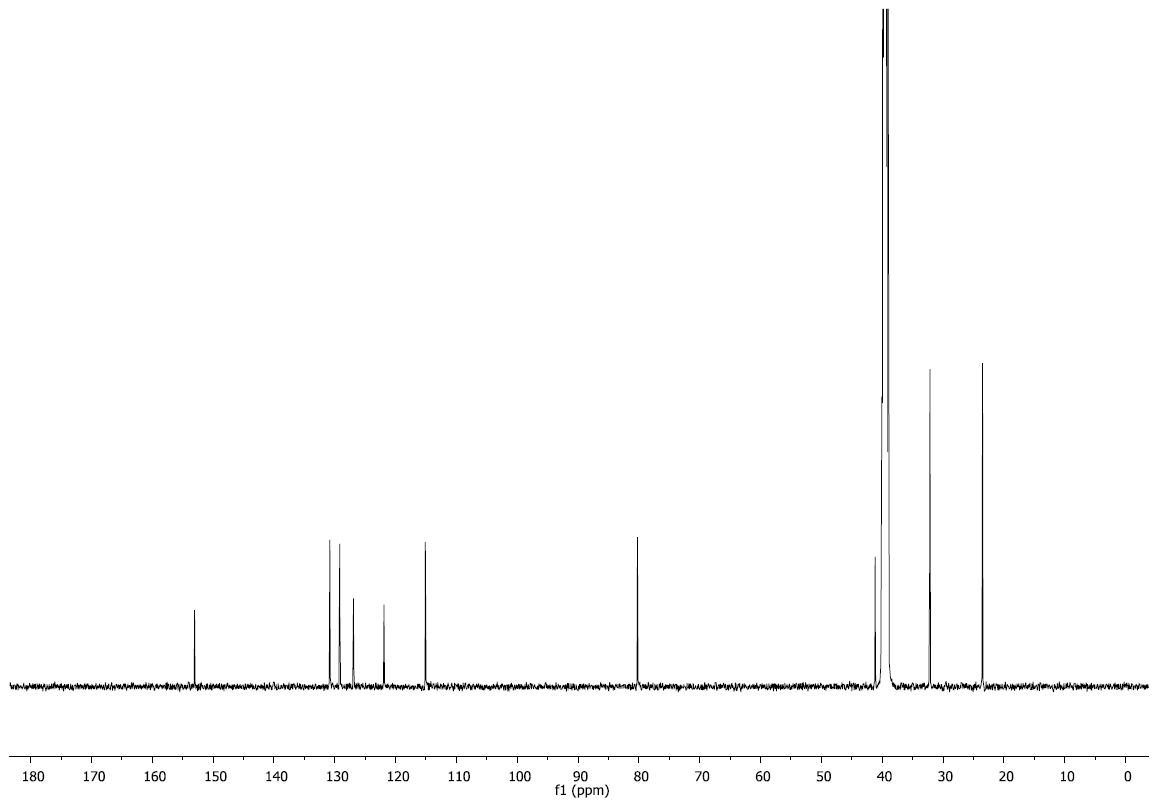


SI_5 Materials and Methods

**Protein expression and purification**

Four constructs of CK2α were used in this study. For kinase activity assays CK2α_WT was used (residues 2-329). For crystallization purposes two different constructs were used: CK2α_KA and CK2α_FP10. CK2α_KA (residues 2-329) contained four mutations designed to aid crystallization by reducing the overall charge of the protein; R21S, K74A, K75A and K76A. CK2α_FP10 contained one mutation (R21S) and an N-terminal extension GSMDIEFDDDADDDGSGSGSGSGS aimed at mimicking a substrate peptide for CK2α. CK2α_FP10 was cloned into pHAT4 vector and CK2α_KA was cloned into pHAT2 vector to give constructs with cleavable His6-tags. Recombinant plasmids containing one of the three constructs (CK2α_WT/ CK2α_KA/ CK2α_FP10/ CK2α_FP9) were introduced into *Escherichia coli* BL21 (DE3) for protein production. Single colonies of the cells were grown in 6x1 L of 2xTY with 100 μg/mL ampicillin at 37 °C. Isopropyl thio-β-Dgalactopyranoside (IPTG) was added to a final concentration of 0.4 mM to induce expression when the optical density at 600 nm reached 0.6. The cells were incubated overnight at 25 °C then harvested by centrifugation at 4,000 *g* for 20 minutes. The same extraction and purification procedure was used for all four constructs, with the exception that CK2α_KA used 350 mM NaCl in the buffer, whereas, CK2α_WT and CK2α_FP10 required 500 mM NaCl. The cell pellets were suspended in 20 mM Tris, 350/500 mM NaCl, pH 8.0) and lysed using a high pressure homogenizer.

Protease inhibitor cocktail tablets (one tablet per 50 mL extract; Roche Diagnostics) and DNase I were then added. The crude cell extract was then centrifuged at 10,000 *g* for 45 minutes, the supernatant was filtered with a 0.22 μm filter. The soluble supernatant was applied on a Ni Sepharose Fast Flow6 column at pH 8.0, washed and eluted in 20 mM Tris pH 8.0, 350/500 mM NaCl, 200 mM imidazole. After overnight dialysis into 20 mM Tris, pH 8.0, 350/500 mM NaCl the Nterminal His6-tag was cleaved overnight by TEV protease and passed through a second metal affinity column to remove uncleaved protein and the protease. The cleaved protein was further purified on a Sepharose Q HP anion-exchange column and the main peak fraction from this column was further purified by gel filtration on a Superdex 75 16/60 HiPrep column equilibrated with Tris 20 mM, pH 8.0, 350/500 mM NaCl. Pure protein was concentrated to 15 mg/mL and flash frozen in liquid nitrogen.

**FP assays**

All fragments were subjected to fluorescence polarisation (FP) binding assays. The three-component system contains a fluorescently labelled peptide, a larger protein and the inhibitor. The sample is illuminated with monochromatic light, corresponding to the wavelength absorbed by the labelled peptide. The excited peptide emits light with a degree of polarisation which is inversely proportional to the rate of rotation. When the labelled peptide is bound to the protein (before the addition of the inhibitor), the large complex tumbles slowly and emits light with a high degree of polarisation. When an inhibitor is added, the fluorescently labelled peptide is displaced and tumbles rapidly in solution, thereby emitting light with a low degree of polarisation (depolarised light). Thus, a decrease in polarised light upon addition of the macrocycle would provide evidence of binding.

Approximate IC50s were determined using a PHERAstar FS plate reader (BMG labtech). The fluorescein probe was measured using 485 nm excitation and 530 nm emission. The fluorescein probe was covalent linked to the N-terminal of the linear CK2beta based peptide RLYGFKIHPMAYQLQ (CK2beta_pep). IC50s were measured using a range of concentrations of the test compound at a constant concentration of DMSO. The experiments were performed in a 384 well plate with final concentrations of 450 nM CK2alpha, 7.4 uM CK2Beta, 350 mM NaCl, 20 mM Mes pH 6.5. The plates were read after a 10 minute incubation period.

**Kinase assays**

IC_50_s were determined using a PHERAstar FS plate reader (BMG labtech). The fluorescein probe was measured using 485 nm excitation and 530 nm emission. The fluorescein probe was covalently linked to the N-terminus of the linear CK2β-based peptide RLYGFKIHPMAYQLQ (CK2β_pep). IC_50_s were measured using 3 mM, 2 mM, 1 mM, 0.5 mM, 0.25 mM, 0.125 mM and 0 mM of the test compound at a constant concentration of DMSO. The experiments were performed in a 384 well plate with final concentrations of 450 nM CK2α, 7.4 μM CK2β_pep, 350 mM NaCl, 20 mM Mes pH 6.5. The plates were read after a 10 min incubation period.^[[1]](#footnote-1)^

**X-ray crystallography**

CK2α_KA at 5 mg/mL in 20 mM Tris, pH 8.0, 350 mM NaCl, 1 mM DTT, and 25 mM ATP was

crystallised with 112.5 mM MES pH 6.5, 35% glycerol ethoxylate and 180 mM ammonium acetate in a 1:1 ratio with a total volume of 2 μL by the hanging drop vapour-diffusion method. The fragments were soaked as singletons at 2-100 mM into these crystals for 15–20 h in 107 mM MES pH 6.5, 35% glycerol ethoxylate and 1.04 M ammonium acetate after which the crystals were cryo-cooled in liquid nitrogen for data collection.

CK2α_FP10 at 10 mg/mL in 20 mM Tris, pH 8.0, 500 mM NaCl, 4 mM DTT, 13 mM ATP, 2 mM phytic acid was crystallised with 107 mM MES, pH 6.5, 29% glycerol ethoxylate, 1.04 M ammonium acetate in a 1:1 ratio with a total volume of 2 μL by the hanging drop vapour-diffusion method. The fragments were soaked into the crystals of CK2α_FP10 for 15–20 h at 100 mM in 107 mM MES pH 6.5, 29% glycerol ethoxylate and 1.04 M ammonium acetate. The crystals were cryo-cooled in liquid nitrogen in the same solution for data collection. X-ray diffraction data was collected at ESRF and Diamond synchrotron radiation sources, then processed using the pipedream package by Global Phasing Ltd; structures were solved by using programs from the CCP4 package. Models were iteratively refined and rebuilt by using AutoBuster26 and Coot programs. Ligand coordinates and restraints were generated from their SMILES strings using the Grade27 software package.

Table 1S – Crystallographic data collection statistics

| **Pdb** | **6GIH** | **6GMD** |
| --- | --- | --- |
| Ligand | CAM187 | Compound 3 |
|  |  |  |
| **Data Collection:** |  |  |
| Beamline | In house | Diamond I24 |
| Wavelength | 1.54 | 0.9686 |
| Resolution range | 35.75-1.96 (2.00-1.96) | 66.65-1.66 (1.70-1.66) |
| Space group | P 1 21 1 | C 2 2 21 |
| Cell (a b c) | 59.49 45.12 62.89 | 64.74 68.84 333.26 |
| Cell (alpha beta gamma) | 90.00 111.23 90.00 | 90.00 90.00 90.00 |
| Total reflections | 194010 (1184) | 687678 (44116) |
| Unique reflections | 21348 (969) | 88560 (6484) |
| Multiplicity | 8.6 (3.5) | 7.8 (6.8) |
| Completeness (%) | 94.9 (81.8) | 100.0 (100.0) |
| Mean I/sigma (I) | 17.7 (2.7) | 8.1 (1.5) |
| R-merge | 0.086 (0.40) | 0.130 (1.88) |
| R-pim | - | 0.050 (0.77) |
| CC-half | - | 0.994 (0.52) |
|  |  |  |
|  |  |  |
| **Refinement:** |  |  |
| R-factor / Rfree | 0.18/0.22 | 0.22/0.25 |
| Number of total atoms | 2995 | 6033 |
| atoms for ligands | 26 | 107 |
| atoms for waters | 197 | 385 |
| Number of polymer residues | 324 | 651 |
| Average/Wilson B-factor | 23.4/20.7 | 25.5/18.1 |
| B-factor for ligands | 25.5 | 33.6 |
| B-factor for solvent | 27.5 | 29.1 |
| RMS(bonds) | 0.01 | 0.0143 |
| RMS(bond angles) | 0.96 | 1.66 |
| RMS(dihedral angles) | 3.2 | 3.5 |
| Crystallisation conditions | 112.5mM Mes pH 6.5, 35% glycerol ethoxylate, 180 mM ammonium acetate | 112.5mM Mes pH 6.5, 35% glycerol ethoxylate, 180 mM ammonium acetate |
| Ligand SMILES | NCC1=CC(=CC(Cl)  =C1)C1=CC  =CC2=C1C=CN2 | [CC(C)C1=C(C=CC=C1)C1  =CC=C(C[NH3+])C=C1Cl |

1. In recent work by Hochscherf and co-workers, FP was also used to identify inhibitors of the α-β PPI in a high-throughput manner: J. Hochscherf, D. Lindenblatt, M. Steinkruger, E. Yoo, O. Ulucan, S. Herzig, O.-G. Issinger, V. Helms, C. Gotz, I. Neundorf, K. Niefind and M. Pietsch, *Anal. Biochem.*, 2015, **468**, 4–14. [↑](#footnote-ref-1)
